# The German National Registry of Primary Immunodeficiencies (2012–2017)

**DOI:** 10.3389/fimmu.2019.01272

**Published:** 2019-07-19

**Authors:** Sabine M. El-Helou, Anika-Kerstin Biegner, Sebastian Bode, Stephan R. Ehl, Maximilian Heeg, Maria E. Maccari, Henrike Ritterbusch, Carsten Speckmann, Stephan Rusch, Raphael Scheible, Klaus Warnatz, Faranaz Atschekzei, Renata Beider, Diana Ernst, Stev Gerschmann, Alexandra Jablonka, Gudrun Mielke, Reinhold E. Schmidt, Gesine Schürmann, Georgios Sogkas, Ulrich H. Baumann, Christian Klemann, Dorothee Viemann, Horst von Bernuth, Renate Krüger, Leif G. Hanitsch, Carmen M. Scheibenbogen, Kirsten Wittke, Michael H. Albert, Anna Eichinger, Fabian Hauck, Christoph Klein, Anita Rack-Hoch, Franz M. Sollinger, Anne Avila, Michael Borte, Stephan Borte, Maria Fasshauer, Anja Hauenherm, Nils Kellner, Anna H. Müller, Anett Ülzen, Peter Bader, Shahrzad Bakhtiar, Jae-Yun Lee, Ursula Heß, Ralf Schubert, Sandra Wölke, Stefan Zielen, Sujal Ghosh, Hans-Juergen Laws, Jennifer Neubert, Prasad T. Oommen, Manfred Hönig, Ansgar Schulz, Sandra Steinmann, Klaus Schwarz, Gregor Dückers, Beate Lamers, Vanessa Langemeyer, Tim Niehues, Sonu Shai, Dagmar Graf, Carmen Müglich, Marc T. Schmalzing, Eva C. Schwaneck, Hans-Peter Tony, Johannes Dirks, Gabriele Haase, Johannes G. Liese, Henner Morbach, Dirk Foell, Antje Hellige, Helmut Wittkowski, Katja Masjosthusmann, Michael Mohr, Linda Geberzahn, Christian M. Hedrich, Christiane Müller, Angela Rösen-Wolff, Joachim Roesler, Antje Zimmermann, Uta Behrends, Nikolaus Rieber, Uwe Schauer, Rupert Handgretinger, Ursula Holzer, Jörg Henes, Lothar Kanz, Christoph Boesecke, Jürgen K. Rockstroh, Carolynne Schwarze-Zander, Jan-Christian Wasmuth, Dagmar Dilloo, Brigitte Hülsmann, Stefan Schönberger, Stefan Schreiber, Rainald Zeuner, Tobias Ankermann, Philipp von Bismarck, Hans-Iko Huppertz, Petra Kaiser-Labusch, Johann Greil, Donate Jakoby, Andreas E. Kulozik, Markus Metzler, Nora Naumann-Bartsch, Bettina Sobik, Norbert Graf, Sabine Heine, Robin Kobbe, Kai Lehmberg, Ingo Müller, Friedrich Herrmann, Gerd Horneff, Ariane Klein, Joachim Peitz, Nadine Schmidt, Stefan Bielack, Ute Groß-Wieltsch, Carl F. Classen, Jessica Klasen, Peter Deutz, Dirk Kamitz, Lisa Lassay, Klaus Tenbrock, Norbert Wagner, Benedikt Bernbeck, Bastian Brummel, Eusebia Lara-Villacanas, Esther Münstermann, Dominik T. Schneider, Nadine Tietsch, Marco Westkemper, Michael Weiß, Christof Kramm, Ingrid Kühnle, Silke Kullmann, Hermann Girschick, Christof Specker, Elisabeth Vinnemeier-Laubenthal, Henriette Haenicke, Claudia Schulz, Lothar Schweigerer, Thomas G. Müller, Martina Stiefel, Bernd H. Belohradsky, Veronika Soetedjo, Gerhard Kindle, Bodo Grimbacher

**Affiliations:** ^1^Institute for Immunodeficiency, Center for Chronic Immunodeficiency (CCI), Medical Center - University of Freiburg, Faculty of Medicine, University of Freiburg, Freiburg, Germany; ^2^RESIST - Cluster of Excellence 2155 to Hanover Medical School, Satellite Center Freiburg, Freiburg, Germany; ^3^Center for Pediatrics and Adolescent Medicine, Medical Center, Faculty of Medicine, University of Freiburg, Freiburg, Germany; ^4^Central Facility Biobanking, Medical Center and Faculty of Medicine, University of Freiburg, Freiburg, Germany; ^5^Institute for Medical Biometry and Statistics, Faculty of Medicine and Medical Center, University of Freiburg, Freiburg, Germany; ^6^Department of Rheumatology and Clinical Immunology, Medical Center - University of Freiburg, Faculty of Medicine, University of Freiburg, Freiburg, Germany; ^7^Center for Chronic Immunodeficiency, Medical Center - University of Freiburg, Faculty of Medicine, University of Freiburg, Freiburg, Germany; ^8^Department of Clinical Immunology and Rheumatology, Hannover Medical School, Hannover, Germany; ^9^Department of Paediatric Pulmonology, Allergy and Neonatology, Hannover Medical School, Hannover, Germany; ^10^Department of Pediatric Pneumology, Immunology and Intensive Care, Charité – Universitätsmedizin Berlin, Berlin, Germany; ^11^Outpatient Clinic for Immunodeficiencies, Institute Medical Immunology, Charité – Universitätsmedizin Berlin, Berlin, Germany; ^12^Department of Pediatrics, Dr. von Hauner Children's Hospital, University Hospital, Ludwig-Maximilians-University Munich, Munich, Germany; ^13^ImmunoDeficiencyCenter Leipzig (IDCL), Hospital St. Georg gGmbH Leipzig, Academic Teaching Hospital of the University of Leipzig, Leipzig, Germany; ^14^Division for Stem Cell Transplantation and Immunology, Department for Children and Adolescents, Frankfurt University Hospital, Frankfurt, Germany; ^15^Department for Children and Adolescents, Division for Allergology, Pneumology and Cystic Fibrosis, University Hospital Goethe University, Frankfurt am Main, Germany; ^16^Department of Pediatric Oncology, Hematology and Clinical Immunology, Medical Faculty, University Children's Hospital, Heinrich-Heine-University, Düsseldorf, Germany; ^17^Department of Pediatrics, University Medical Center Ulm, Ulm, Germany; ^18^Institute for Clinical Transfusion Medicine and Immunogenetics Ulm, German Red Cross Blood Service Baden-Württemberg - Hessen and Institute for Transfusion Medicine, University Ulm, Ulm, Germany; ^19^Centre for Child and Adolescenct Health, Helios Klinikum Krefeld, Krefeld, Germany; ^20^MVZ Dr. Reising-Ackermann und Kollegen, Leipzig, Germany; ^21^Rheumatology/Clinical Immunology, Department of Internal Medicine II, University Hospital Würzburg, Würzburg, Germany; ^22^Pediatric Immunology, Department of Pediatrics, University Hospital Würzburg, Würzburg, Germany; ^23^Department of Pediatric Rheumatology and Immunology, University Children's Hospital, Muenster, Germany; ^24^Department of General Pediatrics, University Children's Hospital Muenster, Muenster, Germany; ^25^Department of Hematology, Oncology and Respiratory Medicine, University Hospital Muenster, Muenster, Germany; ^26^Department of Pediatrics, University Hospital Carl Gustav Carus, TU Dresden, Dresden, Germany; ^27^Department of Women's and Children's Health, Institute of Translational Medicine, University of Liverpool, Liverpool, United Kingdom; ^28^Department of Pediatrics, Kinderklinik München Schwabing, StKM GmbH und Klinikum Rechts der Isar, Technische Universität München, Munich, Germany; ^29^Department of Oncology/Haematology, University Children's Hospital Tübingen, Tuebingen, Germany; ^30^University Children's Hospital, Ruhr University Bochum, Bochum, Germany; ^31^Department of Internal Medicine II (Oncology, Hematology, Rheumatology, Immunology), University Hospital Tübingen, Tuebingen, Germany; ^32^Department of Internal Medicine I, Bonn University Hospital, Bonn, Germany; ^33^Department of Paediatric Haematology and Oncology, Bonn University Hospital, Bonn, Germany; ^34^Department of General Internal Medicine, University Hospital Schleswig-Holstein, Kiel, Germany; ^35^Klinik für Kinder und Jugendmedizin I, University Medical Center Schleswig-Holstein, Campus Kiel, Kiel, Germany; ^36^Prof.-Hess Childrens Hospital, Klinikum Bremen-Mitte, Bremen, Germany; ^37^Department of Pediatric Oncology, Hematology and Immunology and Hopp Children's Tumor Center, University of Heidelberg, Heidelberg, Germany; ^38^Division of Pediatric Hematology and Oncology, Department of Pediatrics and Adolescent Medicine, University Hospital Erlangen, Erlangen, Germany; ^39^Department of Paediatric Haematology and Oncology, Saarland University Homburg, Homburg, Germany; ^40^Division for Pediatric Stem Cell Transplantation and Immunology, University Medical Center Hamburg-Eppendorf, Hamburg, Germany; ^41^Department of Pediatrics, Asklepios Clinic Sankt Augustin, Sankt Augustin, Germany; ^42^Department of Pediatric and Adolescents Medicine, Medical Faculty, University Hospital of Cologne, Cologne, Germany; ^43^Pediatrics 5 (Oncology, Hematology, Immunology), Center for Pediatric, Adolescent and Women's Medicine, Klinikum Stuttgart - Olgahospital, Stuttgart, Germany; ^44^Oncology Hematology Division, Department for Children and Adolescents, University Medicine Rostock, Rostock, Germany; ^45^RWTH University Hospital, Aachen, Germany; ^46^Clinic of Pediatrics, Klinikum Dortmund, Dortmund, Germany; ^47^Department of Pediatrics, Children's Hospital Amsterdamer Strasse, Cologne, Germany; ^48^Division of Pediatric Hematology and Oncology, University Medical Center Göttingen, Göttingen, Germany; ^49^Department of Pediatrics, Vivantes Clinic, Berlin, Germany; ^50^Clinic of Rheumatology and Clinical Immunology, Hospitals Essen-Mitte, Essen, Germany; ^51^Department of Pediatric and Adolescents Medicine, Helios Hospital Berlin-Buch, Berlin, Germany; ^52^Department for Pediatrics I, Martin Luther University Hospital, Halle, Germany; ^53^dsai - Deutsche Selbsthilfe Angeborene Immundefekte e.V. (Patient Organization) e.V., Schnaitsee, Germany; ^54^DZIF – German Center for Infection Research, Satellite Center Freiburg, Freiburg, Germany; ^55^CIBSS – Centre for Integrative Biological Signalling Studies, University of Freiburg, Freiburg, Germany

**Keywords:** registry for primary immunodeficiency, primary immunodeficiency (PID), German PID-NET registry, PID prevalence, European Society for Immunodeficiencies (ESID), IgG substitution therapy, CVID

## Abstract

**Introduction:** The German PID-NET registry was founded in 2009, serving as the first national registry of patients with primary immunodeficiencies (PID) in Germany. It is part of the European Society for Immunodeficiencies (ESID) registry. The primary purpose of the registry is to gather data on the epidemiology, diagnostic delay, diagnosis, and treatment of PIDs.

**Methods:** Clinical and laboratory data was collected from 2,453 patients from 36 German PID centres in an online registry. Data was analysed with the software Stata® and Excel.

**Results:** The minimum prevalence of PID in Germany is 2.72 per 100,000 inhabitants. Among patients aged 1–25, there was a clear predominance of males. The median age of living patients ranged between 7 and 40 years, depending on the respective PID. Predominantly antibody disorders were the most prevalent group with 57% of all 2,453 PID patients (including 728 CVID patients). A gene defect was identified in 36% of patients. Familial cases were observed in 21% of patients. The age of onset for presenting symptoms ranged from birth to late adulthood (range 0–88 years). Presenting symptoms comprised infections (74%) and immune dysregulation (22%). Ninety-three patients were diagnosed without prior clinical symptoms. Regarding the general and clinical diagnostic delay, no PID had undergone a slight decrease within the last decade. However, both, SCID and hyper IgE- syndrome showed a substantial improvement in shortening the time between onset of symptoms and genetic diagnosis. Regarding treatment, 49% of all patients received immunoglobulin G (IgG) substitution (70%—subcutaneous; 29%—intravenous; 1%—unknown). Three-hundred patients underwent at least one hematopoietic stem cell transplantation (HSCT). Five patients had gene therapy.

**Conclusion:** The German PID-NET registry is a precious tool for physicians, researchers, the pharmaceutical industry, politicians, and ultimately the patients, for whom the outcomes will eventually lead to a more timely diagnosis and better treatment.

## Introduction

Primary immunodeficiency disorders (PIDs) represent a group of more than 350 monogenetic, distinct rare diseases. Due to the heterogeneity of the different PID, the exact prevalence is unknown and approximated to be around 1 in 10.000, (1). With the aim of closing this knowledge gap, the PID-NET registry (http://www.pid-net.org/) was founded in 2009 by a consortium of researchers from the Arbeitsgemeinschaft Pädiatrische Immunologie (API, http://www.api-ev.eu/), with funding support from the German Federal Ministry of Education and Research (BMBF).

The primary purpose of the registry is to gather data on the epidemiology, diagnostic delay, diagnosis, and treatment of PIDs. The secondary aim was to establish a support network amongst the physicians who treat PID patients in Germany and other countries across Europe; to enable this, the German PID-NET registry was placed within the framework of the European Society for Immunodeficiencies Registry (ESID registry), an online registry that was created in 2004 and redesigned in June 2014. The objectives of the redesigned version were to make the documentation process more secure, to provide a more concise user interface, and to include the definitions of new research questions on PID. The redesign aimed at reducing the burden of the documentation process to increase patient capture with a minimal defined dataset. Investigator-driven more extensive datasets were encouraged, resulting in 3 levels of registration.

“Level 1” dataset: comprises a minimal set of data (patient's background, way to diagnosis (date and type of presenting symptoms), PID diagnosis, therapy [Immunoglobulin G (IgG) substitution, hematopoietic stem cell transplantation (HSCT), gene therapy], and death report, if applicable with the aim of documenting a complete dataset for each patient once a year.

“Level 2” dataset: for additional information such as laboratory values, imaging or biopsy results, additional clinical features, and further treatment details; aims for more details of the natural history of diseases or disease groups.

“Level 3” dataset: for prospective (clinical) studies on specific genetic diseases for a defined time-span with a comprehensive, study-specific dataset.

While each institution participating in the registry agreed to perform Level 1 registration, Level 2 and 3 registration was voluntary depending on availability of data for the given disease, documentation capacity, and research interests. The registry also serves both as a platform for publications and a study portal. German centres participate in national and international studies such as the unPAD-, CGD-, and APDS-studies (2) (https://esid.org/Working-Parties/Registry/Studies).

## Materials and Methods

The German PID-NET registry contains data from patients (with no age restrictions) in whom a primary immunodeficiency has been diagnosed according to either ESID registry diagnostic criteria or genetic diagnosis^1^. Patients can only be registered if: (i) the documenting centre has obtained a positive vote from the local ethics committee; (ii) an agreement between the treating centre and the ESID has been signed; and (iii) the patient or his/her guardian has signed the PID-NET/ESID patient consent form.

### Centre Characteristics

There were 34 participating centres in Germany as of July 2017. A centre is defined as one or more departments within one hospital, or a healthcare centre for which the ethical approval for PID-NET documentation and an agreement between ESID and the centre have been signed. Seven of the participating centres consisted of 2 different departments within 1 hospital (1 for paediatrics and 1 for adults), with locations in Freiburg, Hannover, Berlin (Charité), Würzburg, Tübingen, Bonn, and Kiel. Additional centres were located in Munich (2 hospitals), Leipzig (2 hospitals and 1 healthcare centre), Frankfurt, Düsseldorf, Ulm, Krefeld, Münster, Dresden, Bochum, Bremen, Heidelberg, Erlangen, Homburg, Hamburg-Eppendorf, Sankt Augustin, Stuttgart, Aachen, Dortmund, Rostock, Cologne, Göttingen, Berlin (2 hospitals), Essen, Halle, and Magdeburg. The children's departments in Düsseldorf, Frankfurt, Leipzig (St. Georg hospital), and Munich (Dr. von Hauner Children's Hospital) also treat adult patients, since there is no corresponding adult department that specializes in the treatment of PID. In Berlin at the Charité, the children's department continues to care for their patients when they reach adulthood, while the other adult PID patients are treated in the adult department (Supplementary Table 1).

### Ethics Committee Vote

In the Federal Republic of Germany, one of the prerequisites that can particularly cause delay in the participation of each centre or hospital department is the requirement for approval from the local ethics committee. This is in contrast to other countries such as France, where only one ethics committee vote is needed for participation of centres/departments across the entire country. German hospitals without an ethics committee must apply to their State Chamber of Medicine (Landesärztekammer) for approval. Once an ethics committee vote is obtained in Germany, it usually does not expire, except in Erlangen after 5 years and in Tübingen after 3 years.

### Technology Platform

The German PID-NET consortium agreed to use the online database platform provided by ESID for the documentation of PID patients. In contrast to the UK, PID-NET Germany does not run a dedicated server for its registry, but directly enters the data from German PID patients into the ESID registry. Therefore, the German PID-NET registry forms the subset of the PID patients entered by German documenting centres into the ESID registry. Although this renders the German PID-NET registry dependent on changes performed on the European level, it also ensures immediate and full implementation of any changes conducted at the European level. This process is further facilitated by the fact that coordination of the ESID online registry is also based at the Centre for Chronic Immunodeficiency (CCI) in Freiburg, Germany.

A redesigned version of the ESID registry went online in June 2014 and contained a number of improvements leading to better data quality: (i) introduction of new datasets, (ii) implementation of novel categories, (iii) automated quality and plausibility checks, (iv) separation of the former complex forms into three levels to achieve a more uniform and complete documentation, and (v) definitions of clinical criteria in cases where a genetic defect is yet to be identified^2^. As an additional quality check, physicians needed to review the data imported from the previous version of the ESID registry and add missing “Level 1” information. The technology platform is described by Scheible et al. (3).

This process entailed screening the 1,997 PID patients entered between the year 2004 and 2014, and then transferring only those marked as “living” and matching the criteria of the new registry over to the new platform. Each imported dataset had to be manually checked and ultimately verified. This process was completed by September 2016 for all 1,404 imported patients. The introduction of new clinical criteria for PID led to the reclassification of some patients; for example from “common variable immunodeficiency (CVID)” to “unclassified antibody deficiency.”

### IT/Data Security

As a yearly update of the registry data is desired, automated reminders sent by email to users at the documenting centres have been introduced. Patient data can be entered via a standard web browser. For secure data transmission, an SSL-protected internet connection with a security certificate is used. All medical data (MDAT) are saved on a server within the Secure Server Net of the computing centre at the University Hospital Freiburg. To separate identification data from medical data for security reasons, a second separate server containing the personal identification data (IDAT) has been established and is operated by an independent trustee. The data from these two sources is only combined for matching names with medical information by eligible users who have logged into the system with their personal username and password. A team at the University of Mainz (https://www.unimedizin-mainz.de/imbei/informatik/ag-verbundforschung/mainzelliste.html) has previously developed a tool for pseudonymisation and record linkage (the so-called Mainzelliste), which was implemented into the ESID registry in 2014. The TMF e.V. (Technologie- und Methodenplattform für die vernetzte medizinische Forschung e.V., www.tmf-ev.de) was consulted for questions relating to programming and data security for the Mainzelliste. This tool automatically identifies duplicate entries in the personalized version, as soon as the entry mask is saved. In such cases, the user receives a message with the notification specifying the centre in which this patient was registered before.

### Coded vs. Personalized Version

German documenting centres can decide whether they wish to register their patient's name, full date of birth, postal code, and city to the Mainzelliste (personalized version), or whether they want to omit such details and keep their own patient-list (coded version). The advantages of using the personalized version include a reduction in documentation errors such as double registration or mixing-up of patients, as the user also can see the name of the patient in the user interface, both in the overview list and at the top of the screen while checking or entering data. However, some centres have opted to use the coded version, mostly due to internal data security policies.

### PID Categories and PID Genes

In the PID-NET registry, 153 different PID diagnoses can be registered, including 11 autoinflammatory disorders. The different PIDs are classified into 9 main categories and 89 subcategories. Currently, there are 273 PID-causing genes available for documentation. However, mutations in some of these genes may cause (up to 6) different PID phenotypes. Therefore, 104 genes are listed twice or more in the PID overview list (Supplementary Table 2)^3^. There is no known monogenetic cause for 24 of the PIDs; 8 of these are different types of “unclassified” PIDs.

### Medical Documentation Specialist

A key contributor to the coordination and support of all participating centres in Germany was the medical documentation specialist, who was funded by the BMBF between November 2009 and March 2018. This post served to fulfil a number of tasks: (i) contacting and recruiting all German centres, (ii) providing support to each centre for gaining local ethics approval, (iii) creating user accounts and dispensing passwords, (iv) designing and creating questionnaires for the registry^4^, (v) making visits to centres, training doctors and study nurses, entering data, (vi) analysis of data with statistical evaluation, (vii) writing abstracts, reports, publications, and articles, such as for newsletters for the German support group for primary immunodeficiency dsai e.V., and (viii) giving presentations and presenting posters at national and international meetings.

Since physicians across different German hospitals have varying degrees of organisation, manpower, time, and motivation to register patients, the PID-NET data is either entered by the treating physician or study nurse, or by the PID-NET documentation specialist who travels to the centres to support their efforts.

### Center Coverage in Germany

Only a few hospitals in Germany have special departments in which physicians focus on adult PID patients. There are large centres for adult PID patients in Freiburg, Hannover, and Berlin. In addition to hospital care, PID patients often receive treatment from local physicians, who usually collaborate with a hospital specialized in treatment of PID. These patients receive their IgG replacement either at their physician's private practice or the local hospital, or they are treated for the symptoms they present with at the time. In order to join the registry, local physicians also have to apply for an ethics committee vote from the State Chamber of Medicine (Landesärztekammer). Besides the administrative paper work associated with this application, further obstacles that deter local physicians from joining the PID-NET registry include the fee for the ethics vote and, in particular, the additional workload required to lodge an application and enter the data.

### Data Set

#### Patients Background

For our analysis, we used the Level 1 dataset which has to be completed for every patient in the PID-NET registry and should be updated yearly. The patient's background profile contains basic information about the month (only for children <12 years) and year of birth (the exact date of birth is not documented for data protection reasons), country of birth and current residence, gender, information on twin status, information on additional familial cases, and potential consanguinity of the parents. To determine the pathway to diagnosis, we retrieved the date of the very first clinical diagnosis of PID, based on laboratory values and/or clinical criteria. The date on which the patient first presented with PID-related symptoms was also recorded. Here, it should be taken into account that onset data are “soft” data, as they depend on the retrospective recollection of patients, relatives and physicians and are therefore often subjective.

#### Way of Diagnosis

To acquire more consistent data on the PID diagnosis, we only documented patients in whom the clinical diagnostic criteria from the ESID registry were fulfilled. These criteria are regularly updated and displayed on the ESID website^5^. Furthermore, if applicable, any affected gene(s) as well as the date of the genetic diagnosis were documented, along with the name of the genetic laboratory, the sequencing method [gene sequencing (Sanger sequencing and gene panels), whole exome/genome sequencing], and other definitive genetic test (such as 22q11 FISH for DiGeorge syndrome)], and the reason for genetic testing.

#### Presenting Symptoms

In contrast to the old ESID online registry, the new version (since June 2014) contains five pre-defined categories for the first presenting symptoms of PID: (1) infections; (2) immune dysregulation such as lymphoproliferation (splenomegaly, hepatomegaly, lymphadenopathy), granuloma formation, autoimmunity (e.g., cytopenia, thyroid disease, joint disease, hepatitis, vitiligo, alopecia, diabetes), inflammatory bowel disease, celiac disease, vasculitis, eczema, and autoinflammatory disease; (3) malignancy; (4) syndromal manifestations: dysmorphic features (such as short stature, facial abnormalities, microcephaly, skeletal abnormalities) and other organ manifestations (such as albinism, hair or tooth abnormalities, heart or kidney defects, hearing abnormalities, primary neurodevelopmental delay, seizures), and (5) other symptoms. The last category allowed the entry of free text. For the analysis, we only included patient data where information was given.

#### Age Distribution

To determine the distribution of the patients by age, we used data from patients marked as living (2,242 patients). In order to obtain the patient's age, we calculated the year of “last news from patient” minus the year of birth; the “date of last news” is defined as the date of the patient's last visit, or the date where information was received, e.g., by telephone. This information was only available for 2,239 patients. In 3 of the patients, data entry had not yet been completed during the verification process. For patients aged 1 year or less, we also incorporated the month into the age calculation. This process then allowed us to refine the age in 25 out of 53 such patients.

#### Diagnostic Delay

To determine the general diagnostic delay, we calculated the time that lapsed between the first presenting symptoms and the date of either the genetic or clinical diagnosis. If both of these dates were available, we used the earlier one. To determine the clinical and genetic diagnostic delays, we calculated the time that lapsed between the onset of the presenting symptoms and the date of clinical and genetic diagnosis, respectively. Patients, that have been “discharged after complete recovery” have been excluded from this analysis. Possible changes in the diagnostic delay were analysed using the Jonckheere-Terpstra trend test. A *p*-value of 0.05 and below was defined as statistically significant. When considering the significance of the diagnostic, clinical or genetic delays, we had to take into account the different number of patients across different PID categories; these numbers varied from 2 to up to 527 for a specific PID.

#### Cause of Death

In order to document the cause of death, we asked for: (i) date of death, (ii) the main causes leading to death (septic shock, heart failure, respiratory failure, liver failure, renal failure, multiple organ failure, haemorrhage, thrombosis, neurological complications, surgical complications, drug toxicity, relapse of malignancy, veno-occlusive disorder, graft vs. host disease, rejection/poor graft function, post-transplant lymphoproliferative disorder, others), and (iii) underlying morbid settings associated with mortality (infection, malignancy, immune dysregulation, transplantation related cause, others). Multiple answers were possible. For the underlying setting leading to death, an additional free text description could be entered optionally. Furthermore, ICD-10 codes could be entered optionally.

#### Treatment

IgG substitution, hematopoietic stem cell transplantation (HSCT), and gene therapy were documented (where applicable). For IgG substitution, data on the IgG product currently in use (brand name), dosage, route and place of administration were requested by the database, along with the patient‘s weight and side effects at the date of last news or current documentation date. Side effects such as anaphylaxis, aseptic meningitis, fever, headache, local side effects (e.g., rash, swelling), renal failure, arterial thrombosis, and venous thrombosis were provided as options for selection. For the option “other side effects” (e.g., rash, swelling), a free text field was available. Regarding HSCT, the date of the procedure, the type of donor, source of CD34 stem cells, the SCETIDE ID (Registry for stem cell transplants for PIDs in Europe—www.scetide.org, in collaboration with ESID), and the EBMT ID (Registry of the European Society for Blood and Marrow Transplantation—www.ebmt.org) were requested by the database. For gene therapy, the date of the gene therapy had to be entered.

### Data Analysis

To generate box plots (**Figure 6** and Supplementary Figures 3 and 4) and to calculate *p*-values, we used Stata® (StataCorp LP, USA). MS Excel (Microsoft, USA) software was used for the remaining statistical analyses.

## Results

### Registration Progress Since Start of PID-NET Registry

**Figure 1:**
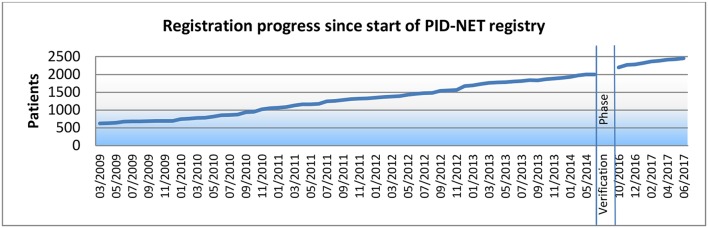
Registration progress since start of PID-NET registry.

When the PID-NET registry was founded in March 2009, 630 patients had already been registered by German centres into the ESID registry. In June 2014, 1,997 patients had been registered into the old version, 1,404 of which were transferred to the new registry system. By July 4, 2017 (data lock), 2,453 patients had been registered. The development of PID patient registration over the last 9 years is depicted in Figure 1.

Of the 2,453 patients, 2,242 were marked as living, 11 as discharged after complete recovery, 51 as deceased, and 149 as lost to follow-up. The 11 discharged patients were diagnosed with: deficiency of specific IgG (SPAD) (1 patient), IgA with IgG subclass deficiency (1 patient), isolated IgG subclass deficiency (3 patients), and transient hypogammaglobulinemia of infancy (6 patients).

In relation to the 82,576,900 inhabitants of Germany (2017)^6^, the 2,242 living PID patients equated to a minimum PID prevalence of 2.72 per 100,000 inhabitants in Germany. According to our previous report in March 2012, the minimum prevalence was calculated as 1.51 per 100,000 inhabitants (4).

There were 2,184 patients (89%) with complete Level 1 data, i.e., with all fields filled-out for these patients. There were 371 patients (15%) registered at centres that did not enter the names of their patients (coded version). Centres that used the Mainzelliste for pseudonymization (personalized version) had a total of 2,082 (85%) registered patients.

### Geographical Distribution

**Figure 2:**
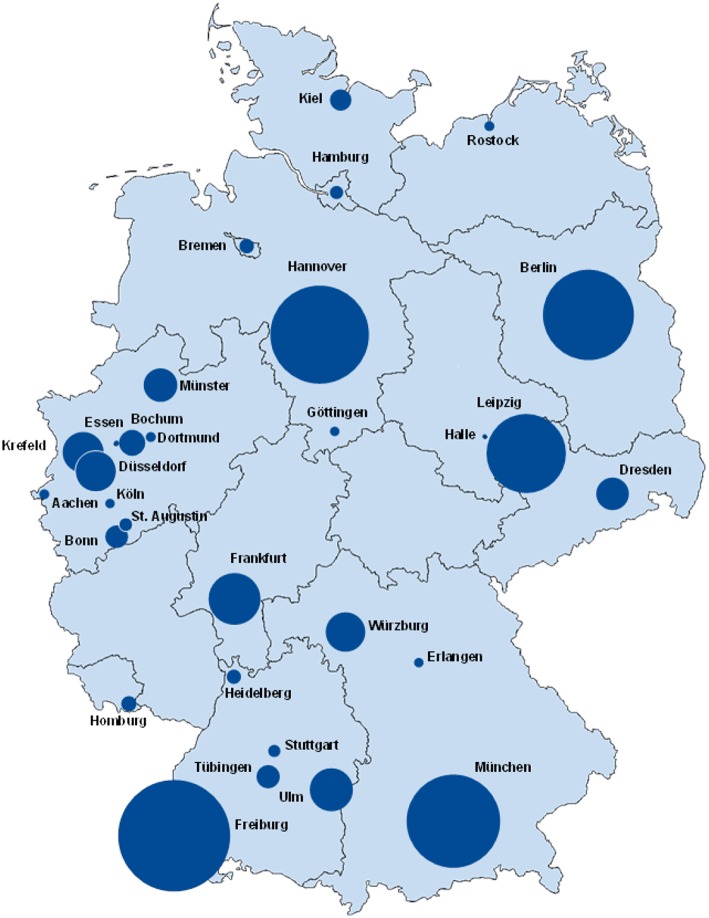
Registered PID-distribution in Germany, July 2017. The size of the circles is proportional to the numbers of patients. In one city there can be one or multiple centres participating in registering patient data. The base map was adapted with the permission of d-maps.com (https://d-maps.com).

The geographical distribution and participation of PID centres in Germany in July 2017 is shown on a map in Figure 2, while the same information from March 2012 can be viewed in Supplementary Figure 5 (4). Supplementary Table 1 displays the geographical distribution of PID patients and the growth in the number of registered patients since our last publication in 2012 (4). The number and distribution of participating centres within the federal states differed immensely: North Rhine-Westphalia has 10 documenting centres, while other federal states only have 1–5 centres. Rhineland-Palatinate and Thuringia do not have any participating centres to date. Small centres may have documented only 1 patient, whereas the largest centre (Freiburg) has 488 documented patients. Of the 2,453 PID patients, 48 (2%) were born outside Germany.

### Most Frequent PIDs and Main PID Categories

Of the 156 PIDs covered by the registry, only 70 different PIDs (45%) were documented for patients in Germany. The 12 most frequent PIDs (each with more than 50 registered patients) accounted for 74% of all PID patients (*n* = 1,825). Specifically, CVID was diagnosed in 728 patients (30%), unclassified antibody deficiency in 267 patients (11%), chronic granulomatous disease (CGD) in 129 patients (5%), agammaglobulinemia in 112 patients (5%), isolated IgG subclass deficiency in 101 patients (4%), severe combined immunodeficiency (SCID) in 83 patients (3%), unclassified immunodeficiency in 76 patients (3%), combined immunodeficiency in 74 patients (3%), selective IgA deficiency in 69 patients (3%), ataxia telangiectasia (A-T) in 66 patients (3%), DiGeorge syndrome (DGS) in 65 patients (3%), and hyper IgE syndrome (HIES) in 55 patients (2%) (Supplementary Table 6).

**Figure 3:**
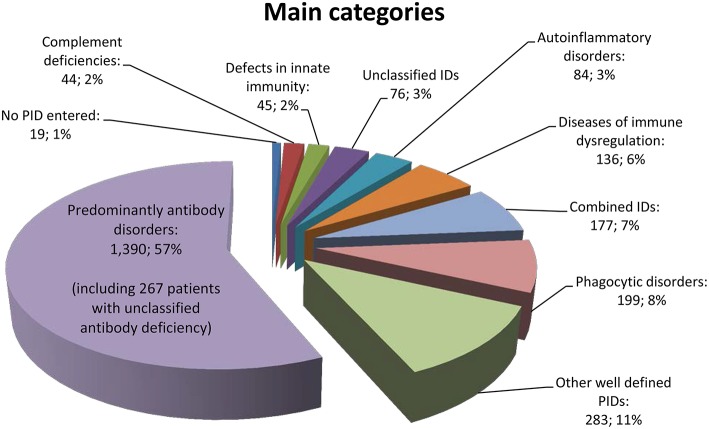
Main categories of PID based on the IUIS classification of 2,453 patients.

The cohort distribution according to the main PID category is shown in Figure 3, and a comparison between patients registered until March 2012 vs. July 2017 can be seen in the Supplementary Table 7. We then questioned whether a shift in percentage had taken place within the main categories of the Gathmann et al. publication (4) vs. our dataset: The biggest difference was a decrease of 6% (from 63 to 57%) in the category of predominantly antibody disorders. Accordingly, combined immunodeficiencies increased from 4 to 7%, and autoimmune and immune dysregulation syndromes increased from 3 to 6%. This is possibly due to the fact that more centres had started documenting children; Another reason could be an increased knowledge about the genetic basis of PID, resulting in an increased classification as CID or autoimmune and immune dysregulation syndromes in patients who had previously been classified as predominantly antibody disorders (including CVID). Other groups remained relatively unchanged.

### Age and Gender Distribution at Date of Last News From Patient

**Figure 4:**
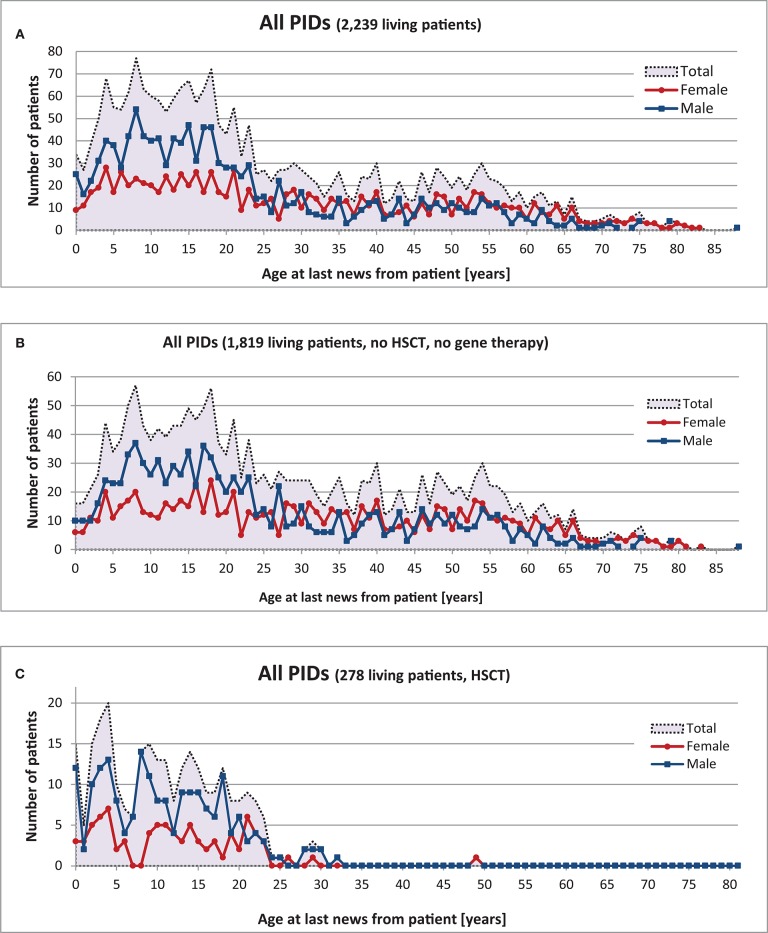
Age and gender distribution of 2,239 living PID patients at “date of last news”. **(A)** Living patients with date of last news, **(B)** Living patients without HSCT or gene therapy, **(C)** Living patients with HSCT.

The gender of 2,242 living patients was available from the registry; 997 (44%) of these were female and 1,245 (56%) male. There was a clear predominance of males amongst patients aged 0–25; however, no statistical differences in gender were found amongst PID patients >25 years old (Figure 4).

There were 1,010 patients (45%) under the age of 18 and 1,229 patients (55%) aged 18 or older. The oldest registered patient was an 88-year-old male with CVID. Comparative analysis of the 12 most prevalent PIDs and Wiskott-Aldrich syndrome (WAS), X-linked lymphoproliferative syndrome (XLP), and familial hemophagocytic lymphohistiocytosis syndromes (FHLH) led to the following results (see also Supplementary Figures 8 and 9): Age distribution varied within each of the different PIDs (Table 1): the oldest registered patients (between 68 and 88 years old) had CVID, unclassified antibody deficiency (80 years old), unclassified immunodeficiencies (79 years old), selective IgA deficiency (75 years old), and isolated IgG subclass deficiency (68 years old). In contrast, the maximum age (years) of patients who had neither HSCT nor gene therapy was 43 for HIES, 21 for SCID, 40 for DGS, 37 for A-T, 31 for Wiskott-Aldrich syndrome (WAS), and 13 for familial hemophagocytic lymphohistiocytosis syndromes (FHLH).

**Table 1:** Age range and median age at last news from patient in years of the 12 most prevalent PID diagnoses and WAS, XLP, and FHLH (patients with no HSCT or gene therapy), living patients.

**12 PIDs, WAS, XLP, FHLH**	**Male (*n*)**	**Female (*n*)**	**Total (no HSCT/no gene therapy)**		**Age range (years)**	**Median age (years)**	**Living patients with and withoutHSCT/gene therapy**
CVID	313	352	665	99.8%	3–88	40	666
Unclassified antibody deficiency	102	119	221	99.1%	0–80	28	223
CGD	46	12	58	51%	0–59	21	114
Agammaglobulinemia	90	7	97	98%	2–54	17	99
Isolated IgG subclass deficiency	40	42	82	100%	4–68	39	82
SCID	6	2	8	11%	0–21	15	76
Unclassified IDs	22	20	42	93%	3–79	20	45
Combined ID	21	17	38	60%	3–57	18	63
DGS	25	27	52	100%	0–40	8	52
Selective IgA deficiency	17	30	47	100%	3–75	23	47
A-T	30	25	55	96%	3–37	13	57
HIES	28	19	47	85%	0–43	16	55
WAS	7	0	7	25%	0–31	20	28
XLP	14	1	15	68%	7–52	19	22
FHLH	3	0	3	17%	2–13	7	18
Total	763	672	1,437	87%	0–88		1,647

The age range (years) of living patients who had undergone an HSCT but no gene therapy was: 8–32 in 7 patients transplanted for X-linked lymphoproliferative syndrome (XLP) (mean = 21); 0–29 in 51 patients with CGD (mean = 12); 0–28 in 64 HSCT-transplant patients with SCID (mean = 10); and 51 for CVID patients (*n* = 1). The oldest living HSCT-transplant PID patient is an XLP patient who underwent a transplant at the age of 32. The median current age of those who had received HSCT was between 5 (FHLH) and 21 (XLP) (Supplementary Table 10). The median current age was similar in male vs. female patients, except for FHLH patients, whose median current age after transplant was 5 years higher in females.

The age of patients (2 SCID patients, 1 CGD patient, and 2 WAS patients) who had undergone gene therapy was 8 months to 5 years at the time of the first gene therapy. One patient who received a gene therapy at the age of 8 month received a second gene therapy at the age of 8 years. Two of these 5 patients also underwent hematopoietic stem cell transplantation, the CGD patient 5 years and the WAS patient 7 years after gene therapy (Supplementary Table 11). For details on age distribution by year of birth, please refer to Supplementary Figures 53–56.

Gender distribution within the 12 most prevalent PIDs as well as XLP, WAS, and FHLH is shown in Supplementary Figure 8. As expected, there were significantly more male patients with X-linked PIDs: agammaglobulinemia (93% male), CGD (80%), XLP (93%), WAS (100%), and FHLH (100%) (Table 1). More detailed information can be seen in Supplementary Figures 12–33.

We then endeavoured to calculate in living patients with the 12 most prevalent PIDs the median age at which the last news from patient occurred, and whether this median age differed across genders.

The highest median age in years of PID patients without HSCT or gene therapy was 39 in isolated IgG subclass deficiency patients, and 40 in CVID patients. The median age of patients with unclassified antibody deficiency was 28. The lowest median age was found in patients with FHLH (median 7) and DGS (median 8) (Table 1). The median age of female patients was generally lower than that of male patients, except in the case of DGS patients (4 years higher).

### Genetics

Out of the 2,453 patients analysed, 893 (36%) had a defined gene defect, as determined by various genetic tests such as (i) Sanger sequencing and gene panels (641 patients, 72%), (ii) whole exome/genome sequencing (22 patients, 2%), and (iii) other definitive genetic test (65 patients, 7%), for 165 patients (18%), no information was available. In addition, 58 patients (2%) were waiting for their genetic test results. In 168 patients (7%), at least one genetic test was performed, but a suspected mutation was not identified. We did not asked for precise information on which genetic test was performed if no mutation had been found. No genetic testing was conducted in 1,005 patients and there was no information available in 329 patients. In other words, in the 1,061 patients in whom a genetic test was completed, a mutation was identified in 84%.

Amongst the 893 PID patients with a genetic defect, 2 (0.2%) were recorded to have been tested neonatally and 9 (1%) prenatally (6 DGS patients with *Del 22q11.2*, 1 SCID patient with *RAG1* and 1 ALPS patient with a *FAS* (*TNFRSF6*) mutation). Of note, in certain PIDs such as CVID, isolated IgG subclass deficiency and selective IgA deficiency, physicians rarely reported genetic testing: Of the 728 patients with CVID, 505 (69%) were marked as not having been genetically tested, while in 165 patients (23%), there was no information available about whether or not genetic testing had taken place. However, amongst the 70 CVID patients for whom genetic testing had been documented, 21 (30%) were registered with a mutation. The results of a genetic test were still pending for 9 CVID patients (11%), while in 49 patients (62%), genetic testing was carried out, but no disease-causing mutation was identified (Supplementary Table 34). Mutations were registered in DGS in 98%, A-T in 95%, CGD in 87%, agammaglobulinemia in 86%, HIES in 84%, and SCID in 76% of patients, respectively (Supplementary Table 35).

We then aimed to determine which genetic diagnosis predominates among the various categories and PIDs. We analysed the main categories and PIDs in which each had at least 20 patients with a genetic defect. It is important to note here that one mutated gene can cause different PIDs, while one PID can be caused by mutations in several genes. Most genetically-diagnosed patients had a gene defect in *BTK* (XLA, 91 patients), which belongs to the main category of predominantly antibody disorders, followed by 80 CGD patients with *GP91-phox (CYBB)* mutation, which belongs to the phagocytic disorders. Sixty-four patients had a *Del 22q11.2* defect (DiGeorge syndrome), 63 had an ataxia telangiectasia mutated gene (*ATM*) (A-T), 36 had a *STAT3* mutation (Hyper IgE syndromes), 24 had a *WASP* mutation [Wiskott-Aldrich syndrome (WAS)]. These four PIDs belong to the main category other well-defined PIDs. Twenty-four patients had a *MEFV* mutation [(familial Mediterranean fever (FMF)], which belongs to the main category autoinflammatory disorders, and 20 patients had a defect in *C1 Inhibitor* (complement deficiency), which belongs to the main category of complement deficiencies (Table 2).

**Table 2:** Most registered gene defects with corresponding main category, subclass and PID with minimum 20 patients affected.

**Gene**	**Main category**	**Subcategory**	**PID**	**Number of patients**	**Living**	**Deceased**	**Lost**	**Female**	**Male**
BTK^*^	Predominantly antibody disorders	Agammaglobulinemia	Agammaglobulinaemias	91	86		5	1	90
gp91-phox (CYBB)^*^	Phagocytic disorders	Chronic granulomatous disease (CGD)	CGD	80	73	4	3	3	77
Del 22q11.2	Other well defined PIDs	DiGeorge syndrome	DGS	64	60		4	33	31
ATM	Other well defined PIDs	DNA-breakage disorder	A-T	63	54	6	3	26	37
STAT3	Other well defined PIDs	Hyper IgE syndromes	HIES	36	36			13	23
WASP^*^^§^	Other well defined PIDs	Wiskott-Aldrich syndrome (WAS)	WAS	24	23		1		24
MEFV	Autoinflammatory disorders	Familial Mediterranean fever (FMF)	FMF	24	20		4	9	15
P47-phox (NCF1)	Phagocytic disorders	Chronic granulomatous disease (CGD)	CGD	24	22	1	1	10	14
C1 Inhibitor	Complement deficiencies	Complement deficiency	HAE (C1Inh)	20	20			15	5

* *Encoded on the X-chromosome*.

§ *Mutations in WAPS were also identified in 7 patients with X-linked thrombocytopenia with mutations in WASP (XLT) and in 2 patients with congenital neutropenia*.

Two-thirds of the 893 patients with a proven genetic defect were male (594, 66.5%) and 299 (33.5%) were female. For some X-linked PIDs, all documented patients were male. This was true for 24 WAS patients with mutations in *WASP*, for 19 XLP patients with mutations in *BIRC4/XIAP*, for 18 patients with X-SCID due to mutations in *IL2RG*, and for 16 patients with a class switch recombination defect (HIGM syndrome) due to mutations in *CD40L/CD154*. In addition, all 7 patients with X-linked thrombocytopenia due to mutations in *WASP* were male.

For X-linked gene defects it is normally expected that only male patients are affected, but there can be exceptions: 1 female out of 91 patients with agammaglobulinemia [with Turner syndrome (X,0)] had a monoallelic mutation in *BTK*, and 1 female out of 10 X-linked lymphoproliferative syndrome (XLP) patients had a mutation in *SH2D1A (XLP1)*. Furthermore, there were 77 male patients (96%) and 3 females amongst individuals with a mutation in *GP91-phox (CYBB)*. Some of the reasons for females being affected with X-linked traits include skewed lyonization and Turner Syndrome.

No gender predilection is expected for autosomal traits; however, we observed a skewed gender distribution for the following autosomal gene defects: *STAT3* (HIES)−64% male, *MEFV* (FMF)−63% male, and *ATM* (A-T)−59% male. The only genetic PID with female predominance was HAE (*C1 Inhibitor*), with 75% females (Supplementary Table 36).

### Familial Cases and Consanguinity

Overall, 521 out of 2,453 (21%) patients had a relative with PID (familial case). These 521 patients came from 403 unrelated families. Twenty-two of the 521 patients were twins (10 identical twins, 8 non-identical twins, 4 twins of unknown status). Amongst the familial cases, 209 (40%) were female and 312 (60%) male.

The highest percentage of these familial cases occurred in CVID (102 patients, 20%), CGD (40 patients; 8%), unclassified antibody deficiency (39 patients; 7%), agammaglobulinemia (33 patients; 6%), SCID (25 patients; 5%), HIES (21 patients; 4%) as well as ALPS and combined ID (each 19 patients, 4%).

We were particularly interested in the proportion of PID patients with consanguineous parents. We found 186 patients (8%) who had related parents. From these 186 patients, 74 had a family member with a PID; however, only 21 of these relatives were registered in the PID-NET registry. Eighteen of them were siblings and 1 was an uncle/aunt. In 9 patients, it was noted that the parents were “probably related”; one such patient had a family member with PID. The highest percentage of consanguinity occurred in the following main categories: 25% (44 patients) in combined immunodeficiencies, 16% (7 patients) in defects of innate immunity, 13% (38 patients) in other well-defined PIDs, and 13% (26 patients) in phagocytic disorders (Supplementary Table 37). Interestingly, a high percentage of familial cases was registered in the main categories with low consanguinities: e.g., in patients with complement deficiencies, the respective percentages of patients from non-consanguineous vs. consanguineous families was 57 vs. 5%; in diseases of immune dysregulation, 41 vs. 11%; and in autoinflammatory disorders, 31 vs. 8% (Supplementary Table 37 and Supplementary Table 38). These data suggest that autosomal-dominant traits may prevail in these particular disorders.

### Presenting Symptoms

For 2,153 of the 2,453 PID patients, information on presenting symptoms was recorded in the registry, including 93 patients with no presenting symptoms.

Amongst these patients, 1,598 (74%) had infections as presenting symptoms. The highest rate of infection was found in patients with CSR/HIGM (Hyper-IgM) syndrome (32 patients, 97%), CMC (15 patients, 94%), isolated IgG subclass deficiency (83 patients, 93%), CVID (577 patients, 92%), and agammaglobulinemia (91 patients, 92%); 547 patients (22%) presented with immune dysregulation. The highest rates of immune dysregulation were found both in 88% ALPS patients (30) and 80% patients with unclassified immune dysregulation (16). There were 190 patients (9%) with syndromal features, which were most frequent amongst patients with Nijmegen breakage syndrome (NBS1) (8 patients, 89%) and DiGeorge syndrome (DGS) (47 patients, 84%). Malignancy was registered as a presenting symptom in 19 patients (1%) (Supplementary Table 39): 10 patients had lymphoma only, 1 had lymphoma and seminoma, 2 had thymoma, 2 had breast cancer only, 1 had an adenocarcinoma, 1 had multiple carcinoma (squamous epithelial carcinoma, colon carcinoma, and carcinoma of the prostate), 1 had breast cancer and multiple carcinoma (adenocarcinoma, squamous epithelial carcinoma, and squamous epithelial carcinoma), and 1 had acute lymphoblastic leukaemia.

“Other presenting symptoms” were documented for 248 patients. Ataxia was mentioned 37 times in the 39 patients with A-T; fever was documented as presenting symptom in 34 patients, of whom 22 had autoinflammatory disorders. Swelling was entered as presenting symptom in 15 of the 23 patients with HAE (C1Inh). Based on the registered patient data, we found no significant gender differences in the kind of presenting symptoms amongst PID patients.

We then wanted to determine whether there were any distinctive features in patients for whom no symptoms were registered. Amongst the 93 asymptomatic patients, 10 (12% of all SCID patients) had SCID, 10 had unclassified antibody deficiency (4% of all patients), 8 had CVID (1% of all CVID patients), 6 had CGD (5% of all CGD patients), 5 had agammaglobulinemia (4% of all agammaglobulinemia patients), 5 had HAE (C1Inh) (22% of all HAE patients), and 5 had transient hypogammaglobulinemia (24% of all transient hypogammaglobulinemia).

Laboratory abnormalities were identified in 55 out of the 93 asymptomatic patients. Most of these had hypogammaglobulinemia (28) or neutropenia (7). In the same group of asymptomatic patients, 52 had a genetic defect in a PID-causing gene.

Genetic testing was performed in 29 patients for family screening and in 14 patients for clinical diagnostic purposes; 7 patients had received a prenatal diagnosis of which 5 patients were a familial case and 16 patients were tested for unknown reasons.

### Age of Onset for Various PIDs

**Figure 5:**
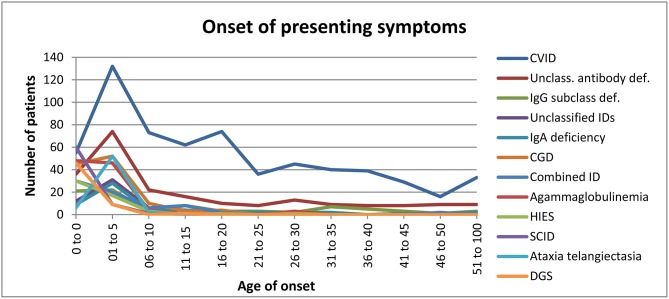
Onset of presenting symptoms.

Regarding the age of onset for the 12 most common PIDs [(Figure 5) and Supplementary Table 40], we observed that: (i) disease onset mainly occurred in the first year of life in patients with SCID (86%), DGS (82%), HIES (58%), agammaglobulinemia (47%), and combined ID (44%) (Supplementary Figure 41); (ii) symptom onset mainly occurred between the ages of 1–5 amongst patients with the following PIDs: isolated IgG subclass deficiency (27%), unclassified IDs (27%), selective IgA deficiency (48%), CGD (46%), and A-T (87%) (Supplementary Figure 42); (iii) in addition to the main onset age of 1–5 years, late disease onset was also often observed in patients with CVID (21%) and unclassified antibody deficiency (33%) (Supplementary Figure 43).

In two patients, the date of genetic diagnosis preceded the onset of symptoms, since both had already undergone prenatal genetic analysis. One ALPS patient with a *FAS (TNFRSF6)* mutation was tested prenatally because her brother was already diagnosed with a PID. Her first symptoms presented 13 months after birth. The second patient had a *Del 22q11.2* mutation (DiGeorge syndrome) with the first presenting symptoms occurring during the first year of life (Supplementary Table 44).

In patients with an inborn error of immunity and an identified gene defect, the first presenting symptoms can still occur even after the age of 20, e.g., in our cohort, one patient with agammaglobulinemia due to a *BTK* mutation first showed manifestation of the disease at the age of 30, one patient with CSR/HIGM (Hyper-IgM) due to a *PMS2* mutation showed manifestation at the age of 36, and one patient with CSR/HIGM (Hyper-IgM) due to a *CD40L* mutation showed manifestation at the age of 44. One HIES patient with a *STAT3* mutation showed manifestation of the disease at the age of 30 and was diagnosed in the following year. Two ALPS patients with mutations in *CD95 (germline - ALPS IA)* and *FAS (TNFRSF6)* developed their presenting symptoms at the ages of 22 and 25, respectively.

### Age at Diagnosis

We next calculated the percentage of PID patients who were diagnosed before vs. at or after the age of 18. Amongst the 12 most-diagnosed PIDs, the highest percentage (>75%) of patients diagnosed before the age of 18 were as follows: all 77 SCID and all 63 A-T patients were actually diagnosed before age of 13 years; 103 patients (95%) with agammaglobulinemia were diagnosed before age of 18 (no patients were diagnosed between the ages of 13 and 18) and 5% between the ages of 18 and 44; 60 patients (94%) with DGS and 113 patients (92%) with CGD were diagnosed before the age of 18. The highest age at which diagnosis occurred in a CGD patient was 58, in DGS patients 31; 47 patients (87%) with HIES and 61 patients (86%) with combined ID were under 18 years of age, the oldest diagnosed HIES patient was 34 and the oldest combined ID patient was 53.

CVID provides the best example for showing that PID can also be diagnosed in adult patients, with 457 patients (65%) diagnosed at or after the age of 18 years. One patient even received his diagnosis at 79 years of age. Patients who were diagnosed with PID at a rate between 45 and 49% at 18 and above had selective IgA deficiency (31 patients), isolated IgG subclass deficiency (45 patients), and unclassified antibody deficiency (111 patients). The oldest diagnosed patients in this particular group were 75 and 79 years of age (unclassified antibody deficiency) and the oldest was 81 (isolated IgG subclass deficiency).

Amongst all the registered PID patients, 4 different PIDs were prevalent in patients who were older than 18 at the time of diagnosis: 2 patients with properdin P factor complement deficiency (PFC) were each 19, 1 patient with mannose-binding lectin deficiency (MBL) was 41, 1 patient with Steinert myotonica dystrophia was 44 and the other two were each 59; 2 patients who had thymoma with immunodeficiency were 47 and 67.

The youngest patient diagnosed within the main category complement deficiency was 6 years old (a patient with unclassified complement ID). For more details see Supplementary Table 45.

### Diagnostic Delay

**Figure 6:**
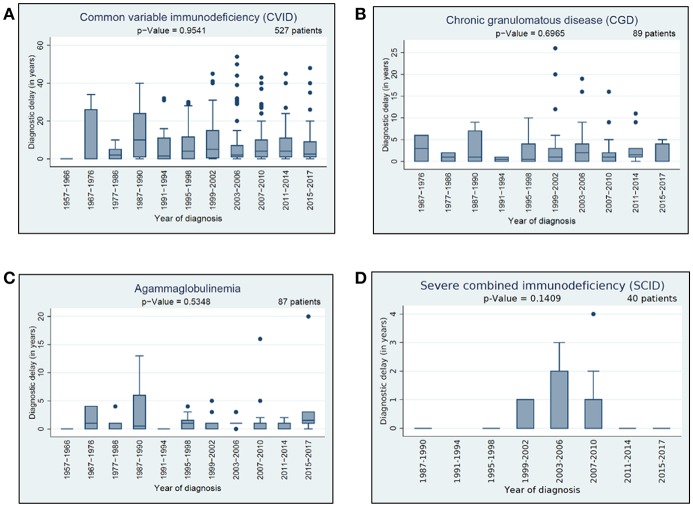
Diagnostic delay: **(A)** diagnostic delay of 527 CVID patients, **(B)** diagnostic delay of 89 CGD patients, **(C)** diagnostic delay of 87 agammaglobulinemia patients, **(D)** diagnostic delay of 40 SCID patients.

We had a closer look to three different types of delay: (i) general diagnostic delay, (ii) clinical delay, and (iii) genetic delay. For information related to general diagnostic delay, data from 1,643 patients were available; namely, the year of symptom onset and the date of diagnosis (genetic or clinical). We scrutinized these data in the context of the 12 most prevalent PIDs and speculated whether the diagnostic delay between the presenting symptoms and diagnosis had decreased over time. Detailed information about the general diagnostic delay in 4 of the 12 most prevalent PIDs is presented in Figure 6. Amongst the 12 most prevalent PIDs, we observed no significant decrease neither in general diagnostic delay nor in the clinical diagnostic delay over the last 5 decades.

Data on clinical diagnostic delay were available for 1,596 patients and analysed in the context of the most prevalent 12 PIDs. The longest clinical delay was in CVID, isolated IgG subclass deficiency, and selective IgA deficiency (average: around 7 years, median: 3 years), followed by HIES (average: 5.33 years, median 3 years). The lowest median delay was for DGS (average: 0.96 years) and SCID (average: 0.43 years), see Table 3.

**Table 3:** Average and median of clinical diagnostic delay.

**12 PIDs**	Number of patients^*^	**Patients with clinical diagnostic delay information**	**Average of clinical delay (years)**	**Median of clinical delay (years)**
CVID	527	527	7.35	3
Isolated IgG subclass deficiency	64	64	7.22	3
Selective IgA deficiency	37	37	6.78	3
Unclassified antibody deficiency	168	168	5.69	2
HIES	43	42	5.33	3
Combined ID	50	50	4.53	2
Unclassified IDs	49	49	3.96	2
CGD	89	85	3.01	1
A-T	49	47	1.96	1
Agammaglobulinemia	87	80	1.60	1
DGS	38	25	0.96	0
SCID	40	40	0.43	0
Total	1,243	1,216	5.52	2

* *Year of onset and year of clinical diagnosis and or genetic diagnosis*.

Data on genetic diagnostic delay were available for 554 patients (the genetic diagnosis was available before the clinical diagnosis in 14 cases of different PIDs, but none was made prenatally). Data relative to the 12 most prevalent PIDs are displayed in Supplementary Table 46. CVID patients had the highest genetic diagnostic delay (average: 11.08 years, median: 9 years), while DGS patients had the lowest (average: 0.48 years, median 0 years). The average genetic delay in SCID patients was 1.42 years and the median 0.5 years.

With regard to genetic diagnostic delay, a significant improvement was documented in SCID patients (*p* = 0.0074), as well as HIES patients (*p* = 0.037) (Supplementary Figures 3 and 4). Between 1997 and 2006, the average genetic diagnostic delay in SCID patients was 2.43 years and the median was 1 year, whereas between 2007 and 2017 the average delay was 1 year and the median was 0.

### Deceased Patients

Out of 2,453 patients, 51 patients (2%) were registered as deceased of whom 14 patients had received HSCT. In 2 of those 14 patients (aged 5 and 9 years, CGD), the graft vs. host disease was explicitly documented as cause of death. Main causes leading to death for the other patients were mostly respiratory failure (16 patients, 31%) and multiple organ failure (13 patients, 25%). As underlying cause, infections were mentioned for 20 patients (39%). The age of death ranged between 4 months (surgical complications, multiple organ failure, CGD) and 88 years (cause of death is unknown, CVID) (Supplementary Table 47).

### Treatment

Three types of treatment were documented in the registry's Level 1 dataset: (i) IgG substitution, (ii) hematopoietic stem cell transplantation (HSCT), and (iii) gene therapy.

Of the 2,453 registered patients, 1,203 (49%) received IgG replacement at the date of last news, 850 (35%) did not receive Ig-replacement and for 400 patients (16%), no information was available. The main categories with the highest proportion of patients receiving IgG substitution were predominantly antibody disorders (71% of 981 patients), followed by defects in innate immunity (42% of 19 patients), combined immunodeficiencies (32% of 57 patients), unclassified immunodeficiencies (32% of 24 patients), and other well-defined PIDs (30% of 86 patients) (Table 4).

**Table 4:** IgG substitution referring to main categories and foremost PIDs.

**Main categories/PID**	**Total patient number**	**Number of patients receiving Ig-replacement**	**Number of patients receiving Ig-replacement (%)**	**Percentage out of 1,203 patients with Ig replacement (%)**
Predominantly antibody disorders	1,390	981	71	82
CVID	728	630	87	52
Unclassified antibody deficiency	267	150	56	12
Agammaglobulinemia	112	97	87	8
Isolated IgG subclass deficiency	101	57	56	5
CSR/HIGM (Hyper-IgM)	38	22	58	2
IgA with IgG subclass deficiency	26	15	58	1.2
Defects in innate immunity	45	19	42	2
Combined immunodeficiencies	177	57	33	5
Combined ID	74	33	45	3
SCID	83	20	24	2
Unclassified Immunodeficiencies	76	24	32	2
Other well defined PIDs	283	86	30	7
A-T	66	25	38	
HIES	55	20	36	2
Unclassified syndromic PID	16	10	63	1
Diseases of immune dysregulation	136	19	14	2
Phagocytic disorders	199	9	5	0.7
Complement deficiencies	44	2	5	0.2
Autoinflammatory disorders	84	1	1	0.1
No PID entered	19	5	26	0.4
Total	2,453	1,203	49	100

More than half (52%) the patients on IgG treatment had CVID, 12% had an unclassified antibody deficiency, 8% had agammaglobulinemia, and 5% had isolated IgG subclass deficiency. Other PIDs were represented by <4% (Table 4).

The share of patients who received IgG replacement within the respective PID diagnosis was: CVID (87%), agammaglobulinemia (87%), unclassified syndromic PID (63%), CSR/HIGM (Hyper-IgM) (58%), IgA with isolated IgG subclass deficiency (58%), unclassified antibody deficiency (56%), and isolated IgG subclass deficiency (56%). All other PIDs comprised of <50% of patients who had been treated by IgG replacement. IgG substitution was not registered amongst the following PIDs (each with more than 10 registered patients): selective IgA deficiency, congenital neutropenia, FMF, HAE (C1Inh), TRAPS, and Shwachman-Diamond-syndrome (Supplementary Table 48).

**Figure 7:**
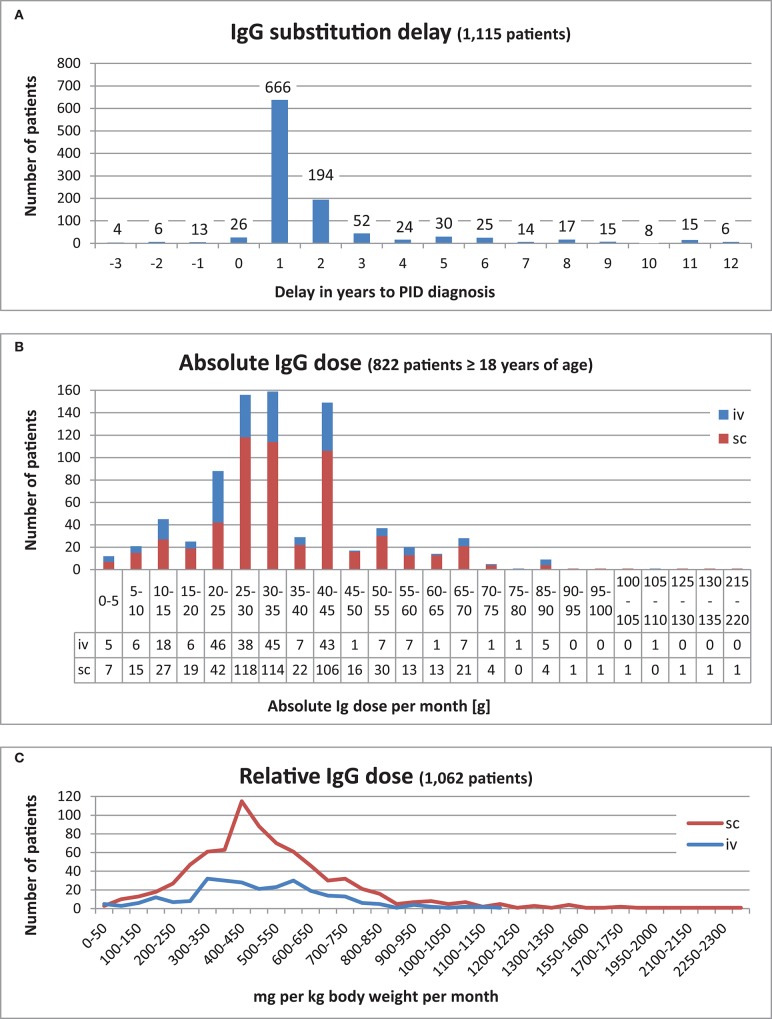
IgG substitution. **(A)** IgG substitution delay for 1,115 patients who received IgG replacement in the range of 3 years before PID diagnosis and 12 years afterwards. **(B)** Absolute IgG dose per month [g] of 822 patients ≥18 years of age. **(C)** Relative IgG dose (mg per kg body weight) for 1,062 patients.

The IgG substitution delay is defined as the time span between diagnosis and first IgG treatment. This data was calculated for 1,189 patients. The time span varied from 20 years prior to the PID diagnosis being established to 53 years post-diagnosis. Both extremes were observed in CVID patients. Most patients (666/56%) received their therapy within the first year of their PID diagnosis being established, while 194 (16%) received therapy within the second year of diagnosis (Figure 7A, an extract). There were 189 patients (16%) who received their first IgG treatment prior to clinical or genetic diagnosis (timespan is between 1 day and up to 20 years before a PID diagnosis). Further details about these patients can be found in Supplementary Table 49.

Regarding the route of administration, 844 of patients (70%) received subcutaneous (sc) IgG infusions, 78 of these patients with the addition of hyaluronidase; 344 (29%) had intravenous (iv) infusions of IgG, while 15 patients (1%) had no documented information about the route of administration.

Amongst the 853 adults (≥18 years old), 261 patients (31%), and 581 patients (68%) received IgG intravenously or subcutaneously, respectively; the route of administration was unknown in 11 patients. Amongst the 350 patients who underwent IgG substitution below the age of 18, 83 (24%) received IgG intravenously, while 263 patients (75%) received it subcutaneously. The route of administration was unknown in 4 children.

We next speculated whether the absolute IgG dose prescribed per month [g] correlated with the respective PID. We therefore analysed the 12 major PIDs and PIDs with minimum 7 patients ≥18 years of age in this context: The minimum IgG dose was between 1 g (CVID) and 35 g (DGS) per month and the highest dose between 28 g (ATM) and 130 g (CVID). Within these PIDs groups, the average was between 20 g (ATM) and 49 g (DGS per month, the median between 21 g (ATM) and 43 g (agammaglobulinemia). The total average of these PIDs was 35 g per month, whereas the total median was 33 g per month (Table 5).

**Table 5:** Absolute IgG dose for patients ≥18 years of age: minimum, maximum, average, and median (minimum 7 patients get IgG substitution).

**PID where minimum 7 patients above 17 years get IgG treatment**	**12 PIDs**	**Number of patients on IgG substitution**	**Number of patients dose per month (g) available**	**Min – Max IgG dose per month (g)**	**Average dose**	**Median dose**
Agammaglobulinemia	x	47	45	22–87	45	43
SCID	x	7	7	27–57	39	41
CVID	x	546	528	1–130	36	35
Combined ID	x	18	15	7–61	35	35
Unclassified antibody def.	x	99	95	3–87	34	33
CSR/HIGM (Hyper-IgM)		10	10	11–61	32	30
IgA with IgG subclass deficiency		13	13	7–43	24	28
Isolated IgG subclass deficiency	x	43	43	7–91	29	27
Unclassified IDs	x	16	14	5–76	29	24
HIES	x	8	8	5–35	23	24
ATM	x	7	6	14–26	20	21
DGS	x	3	3	35–70	49	35
CGD	x	0	–	–	–	–
Selective IgA deficiency	x	0	–	–	–	–
Total		814	784	1–130	35	33

Out of the 822 adult patients who received Ig substitutions, most (315) received 25–35 g per month, 149 patients received 40–45 g per month, and 88 patients received 20–25 g per month (Figure 7B).

To determine the relative replacement dosage in mg IgG per body weight (kg) per month, the available weight and IgG dosage data from 1,062 patients were used from the date at last news. For the sc route, the highest relative doses were between 400 and 450 mg per body weight (kg) per month, whereas for the iv route, there were two peaks, one was between 300 and 400, the other between 550 and 600 mg per kg body weight (kg) per month (Supplementary Table 50 and Figure 7C). The most prevalent dosage interval was 400–450 mg IgG per body weight (kg) per month. The average relative dosage was 517 mg per body weight (kg) per month, and the median was 474 mg per body weight (kg) per month.

To determine how often side effects occurred with IgG substitution, we analysed (i) the general side effects and (ii) the side effects corresponding to the route of application.

**Figure 8:**
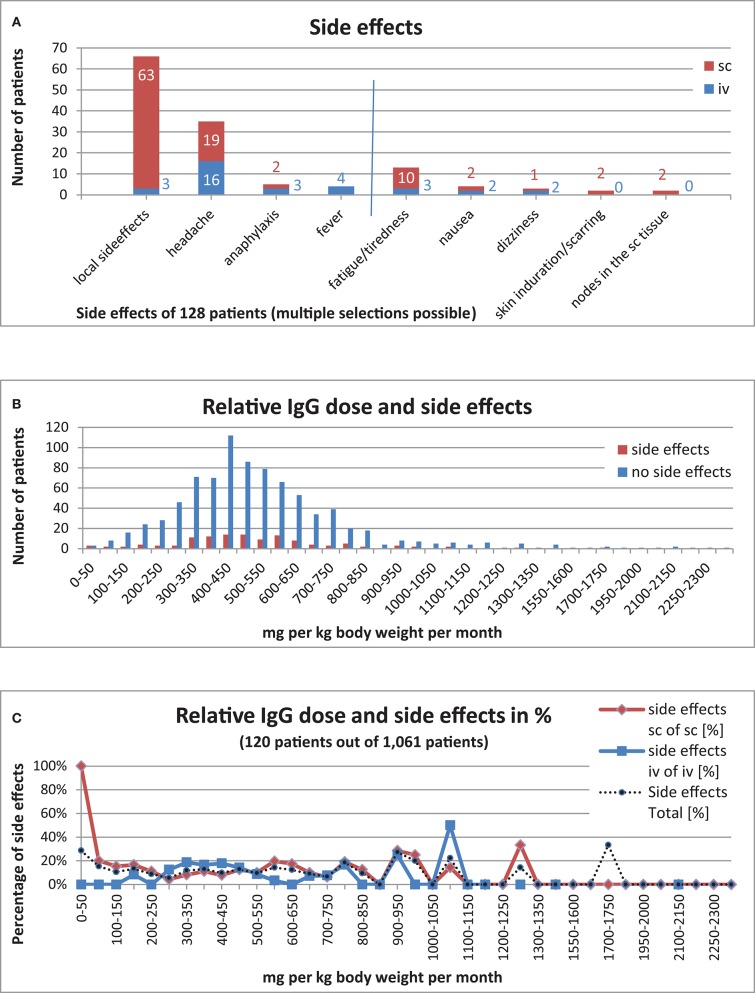
IgG substitution and side effects. **(A)** Side effects in 128 patients (11%) [(iv, 32 patients (25%); sc, 96 patients (75%)] out of 1,140 patients with IgG substitution, included 39 patients [(iv, 13 patients (33%); sc, 26 patients (76%)] with “other side effects.” **(B)** Relative IgG dose and side effects (120 patients with side effects out of 1,061 patients). **(C)** Relative IgG dose and side effects in percent (120 patients with side effects out of 1,061 patients).

Of the 1,203 patients who had received IgG replacement, 128 patients (11%) had documented side effects, whilst 922 (77%) patients were documented to not have had side effects. No information on side effects was available for the remaining 153 patients (13%). Local side effects such as rashes and swelling were recorded for 3 patients undergoing iv replacement but in 63 patients receiving sc IgG treatment. Headache was documented in 16 patients receiving iv replacement and 19 patients receiving sc treatment. Anaphylaxis was documented in 3 patients receiving iv replacement and in 2 patients receiving sc treatment. Fever was documented in 4 patients receiving iv replacement but did not occur in patients under sc treatment. Arterial or venous thrombosis, aseptic meningitis, and renal failures were not reported. In addition, other rare side effects were entered for 39 patients (iv: 13, sc: 26): fatigue/tiredness (iv: 3, sc: 10), nausea (iv: 2, sc: 2), skin induration/scarring (iv: 0, sc: 2), noduli in the subcutaneous tissue (iv: 0, sc: 2), dizziness when infusion ran too quickly (iv: 2), and dizziness without further reasons provided (iv:0, sc:2) (Figure 8A)

We also examined whether a high relative IgG dose per month had an influence on the occurrence of side effects. Information on side effects, Ig dose and weight was only available for 1,061 patients under IG replacement therapy. Side effects were reported in 120 (11%) of these patients. There was no significant correlation between the dosage and the frequency of side effects (Supplementary Table 50 and Figure 8B). Furthermore, whether a patient received subcutaneous vs. intravenous IgG replacement therapy had no significant bearing on the occurrence of side effects (Figure 8C).

A total of 334 HSCTs were registered in 300 patients (12% of all PID patients) between 2004 and June 4, 2017, of which 32 patients had more than one HSCT. Stem cells derived from bone marrow was transplanted 233 times (70%), from peripheral blood 81 times (24%), and from cord blood 8 times (2%). The source of HSCs was not documented in 12 (3%) cases. Most donors were matched unrelated (MUD) to the patient (163 cases, 49%); in 62 cases (19%) they were matched siblings (MSD); in 70 cases (21%) they were haplo-identical donors (one of the parents); and in 16 cases (5%) they were mismatched unrelated donors (MMUD); in 1 case (0.3%) the patient received autologous stem cells; in 14 cases (4%) the transplant originated from an “other related donor”; and in 8 cases (2%) the type of donor was not registered (Table 6).

**Table 6:** Donor and source of HSCT.

**Donor/Source**	**Bone marrow**		**Peripheral blood**		**Cord blood**		**Source unknown**		**HSCT total**	**%**
Matched unrelated (MUD)	124		35		2		2		163	49
Matched sibling (MSD)	56		4		1		1		62	19
Haplo-identical (parent)	30		36		1		3		70	21
Other related donor	12		1		1				14	4
Mismatched unrelated (MMUD)	9		5		2				16	5
Autologous	1		0		0				1	0.3
Unknown	1		0		1		6		8	2
Total	233	68%	81	24%	8	2%	12	3%	334	

Most of the HSCTs were performed in patients from the main category of combined immunodeficiencies (114 of all registered CID patients, 64%), followed by phagocytic disorders (78 patients, 39%), other well-defined PIDs (45 patients, 16%), diseases of immune dysregulation (41 patients, 30%), predominantly antibody disorders (17 patients, 1%), unclassified immunodeficiencies (3 patients, 4%), defects in innate immunity (1 patient, 2%), and autoinflammatory disorders (1 patient, 1%). None of the 44 patients with complement deficiencies received an HSCT.

Most of the 300 patients who had an HSCT were SCID patients. The relative proportion of patients who underwent an HSCT are described according to the PIDs as follows: 72 out of 84 SCID patients (86%), where 2 of these patients underwent additional gene therapy; 61 out of 129 CGD patients (47%); 28 out of 74 patients with combined immunodeficiency (38%); 20 out of 29 patients with Wiskott-Aldrich syndrome (69%); 16 out of 20 FHLH patients (80%); 11 out of 38 patients (29%) with CSR/HIGM (Hyper-IgM syndrome); 9 out of 29 XLP patients (31%); 8 out of 55 HIES patients (15%); and 7 out of 32 patients congenital neutropenia (22%). HSCTs were also carried out in 3 out of 75 patients (4%) with unclassified immunodeficiency; in 2 / 267 patients with unclassified antibody deficiency (0.7%); in 2/112 agammaglobulinemia patients (2%); and in 1/66 A-T patients (2%). Patients with DGS, selective IgA deficiency, and isolated IgG subclass deficiency were not treated with an HSCT. Only 2 out of the 728 CVID patients (0.3%) received an HSCT (for more details see Supplementary Table 51).

Eighty percent (240) of the HSCT patients had already received a genetic diagnosis by the time of transplantation. In 60 patients, HSCT was performed without knowledge of the exact genetic defect. Amongst these 60 patients: the genetic defect could not be identified in 24 patients, despite genetic testing; the genetic test results were still pending for 10 patients at the time of HSCT; 7 patients were marked as not tested, and no information was given for 19 patients.

**Figure 9:**
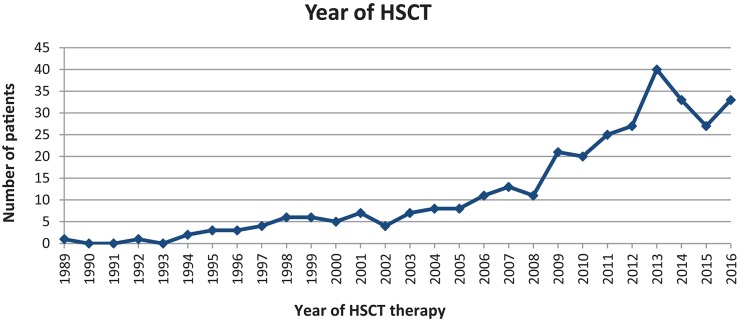
326 HSCTs performed between 1989 and 2016 for 294 patients.

The first HSCT in our cohort was performed in 1989. The second was recorded 3 years later. The frequency of HSCTs increased steadily from 2 in 1994, to up to 40 in 2013. Since then, the number of PID-related HSCTs has stabilised to around 25–40 transplantations per year (Figure 9).

We examined whether IgG treatment was still necessary after an HSCT. Out of the 300 patients who had undergone at least 1 HSCT, 42 (14%) received IgG replacement after HSCT, while 188 patients (62%) did not. There was no information in 69 patients (23%) with regard to IgG substitution after transplant (for details see Supplementary Table 52). Amongst the 41 patients who already had IgG treatment before their HSCT, 18 (44%) continued their treatment, whereas 23 patients (56%) did not. There was no information about 249 patients in terms of IgG treatment prior to HSCT. Following HSCT, 4 patients required IgG substitution for the first time: 3 patients between 6 and 9 months later (with XLP, atypical SCID, and unclassified phagocytic disorders) and 1 SCID patient after 3 years. These patients each had a different PID. For 13 patients, it could not be identified whether they shortly after or before their HSCT they got their IgG substitution as only year and month was given for both dates (4 persons) or only the year (9 patients).

Out of 298 patients who recieved at least one HSCT, 14 passed away. Two of them because of graft vs. host disease (compare section “Deceased patients” above), eight were lost to follow-up, and 276 are registered as “alive.”

Gene therapy was recorded in 5 patients (0.2%). The first case of gene therapy was documented in 2002 in an X-linked SCID patient, the second in 2005 in a *GP91-phox* CGD patient, the third and fourth in 2006 in an ADA-SCID and a WAS patient, respectively. In 2009, the X-linked SCID patient who had been treated in 2002 had a second gene therapy procedure, and in 2012 an additional WAS patient underwent gene therapy. Gene therapy was combined or followed-up by HSCT in 2 of the 5 patients: The ADA-SCID patient had a transplant of autologous bone marrow CD34+ cells engineered with the ADA gene after non-myeloablative conditioning, the WAS patient who had had gene therapy in 2006 at the age of almost 4 had an HSCT at the age of 11. The CGD patient who had gene therapy at the age of 5 had an HSCT at age 10.

We then speculated whether patients who had undergone gene therapy still needed IgG: the 2 patients with X-linked SCID and WAS who received gene therapy but no HSCT still received IgG substitution, despite their gene therapy being successful.

## Discussion

### PID Registries

Electronic PID registries can be found at different administrative levels: databases operated by single centres, national registries, or even international networks such as the ESID registry (5). Although their datasets differ, almost all these databases store a common subset of parameters on PID patients similar to the one covered by Level 1 of the PID-NET registry: PID diagnosis, basic demographic data, (presenting) symptoms, and treatment^7^. Among others, their aim is to identify the prevalence, incidence, and morbidity of PIDs. Many physicians and researchers recognize the importance of PID registries (5–8).

### Prevalence and Under-Reporting

Since its advent in 2009, the PID-NET registry has grown continuously from 630 German patients who were registered by 7 participating centres into the European ESID-Registry, to a current total of 2,453 patients registered by 36 centres. This covers all the major centres that treat PID patients in Germany. However, since it is not mandatory to register PID patients into the PID-NET registry, we are only able to report a minimum prevalence of PID diseases in Germany. The current data reported here yields a minimum prevalence of 2.72 per 100,000 inhabitants in Germany.

In 2006, the Kuwait National Primary Immunodeficiency Disorders Registry (KNPIDR) reported a childhood PID prevalence of 11.98 per 100,000 children (8). Other registries do not report a specific prevalence in children. In Germany, the corresponding value was 7.5 per 100,000 children (1,010 patients in 13,538,146 population under 18 years of age)^8^. Taking into account the data reported: (i) in 2014 by the Swiss registry (4.16/100,000) (9), (ii) the study in Olmsted County, Minnesota (USA) (4.6/100,000) (10), (iii) more recently by the UKPID registry (5.90/100,000) (11), (iv) the CEREDIH (France) (8.0/100,000) based on a recent epidemiological study by Mahlaoui et al. (1), and (v) Iranian Registry (IPIDR) (3,92/100.000) (12), we believe that the current report only covers about one third of PID patients living in Germany. A review article on 22 states from the Middle East and Northern Africa (MENA states) reports an overall prevalence between 0.81 to 30.5 per 1,000,000 inhabitants (13). The number of PID patients in South Africa is estimated to be between 2,850 and 45,728, although the actual reported number of patients in the SA registry is currently only 301 (14). The Latin American Society for Immunodeficiencies (LASID) registry is an international registry of 15 countries of South America representing 599 million inhabitants and reports for 2014 on 5,203 living patients (15) and for 2018 on 7,977 patients (16).

This under-reporting of PID patients into registries might create a bias; hence, the data presented here need to be interpreted carefully. This is also true for other registries, however, only the publication by the IPIDR gives an estimation on the expected underreporting (2.3% of the expected PID patients registered) (12).

There are several reasons why not all PID patients are registered in Germany: The main limiting factor for a complete documentation of all diagnosed PID patients into the registry remains the limited man-power for documentation. This is also true for subsequent quality control (monitoring), which is currently only possible in Level 2 and Level 3 projects.

Hence, the fewer patients are treated in a centre, the more likely they are to be completely registered. The number of physicians and study nurses in relation to the number of patients also has an important bearing on the quality and completeness of the respective datasets. Multiple overlapping registries and more specialized registries—such as the HSCT registries (SCETIDE and EBMT), and AID-Net—may also be a reason for the non-registration of PID patients into the PID-NET registry. The time-consuming ethical approval process deters smaller centres and local physicians from joining the registry. Patients with a less-severe PID who are mainly treated with IgG substitutions to avoid conditions such as infections often prefer to go to a local physician closer to home. As a possible reason for underreporting, it could also be suspected that PID patients do not always give consent for registration. However, according to personal communications with the treating physicians these cases are very rare.

The transition from adolescence to adulthood often leads to a halt in registry documentation, e.g., when the patient leaves the pediatric department and is treated by a local physician who does not participate in the project. This is especially true when one of the few specialized centres that treat adult PID patients is not within the vicinity.

Apart from the under-reporting of patients already diagnosed with PID, we assume there is also a substantial number of patients who are still to be diagnosed. The awareness campaigns that have been launched within the last few years (e.g., FIND-ID, https://www.find-id.net) might help to overcome this issue.

### Distribution Within the IUIS Categories

The distribution of patients within the main IUIS categories in the PID-NET registry is similar to that in the UKPID registry, except in the case of complement deficiency, where the proportion of registered patients among all patients is 13% in the UK (11) and only 2% in Germany. This is most likely due to documentation bias, since HAE patients (C1 deficiency) are seen by immunologists in the UK, but not in Germany. In contrast to the PID-NET registry, the UKPID also registers secondary antibody deficiencies.

The Centre de Référence Déficits Immunitaires Héréditaires (CEREDIH) runs the PID registry in France. Since CEREDIH uses an internal classification system based on the PID diagnoses, and differs from the IUIS classification, we could not directly compare the distribution among the main categories, e.g., CVID is categorized as a B-cell deficiency, which is part of the adaptive PID (17) category.

A similar situation is presented by the Iranian registry (12). However, the main categories of the Iranian registry presented do not match to the ESID registry's categories, e.g., “immunodeficiencies affecting cellular and humoral immunity” is not present in the ESID classification whereas the category “other well-defined PIDs” is not present in the Iranian register.

LASID registry reports of seven categories: predominantly antibody deficiencies (65%), other well-defined PIDs (10%), phagocytic disorders (9%), predominantly T-Cell deficiencies (9%), complement deficiencies (3%), unclassified IDs (3%), and immune dysregulation syndromes (1%) (15).

In Switzerland, the main category with the highest difference to our registry was predominantly antibody disorders, with 62.4% compared to 57% in Germany (9).The Kuwait registry named six main categories for their 176 patients: complement deficiencies (4%, Germany: 2%), diseases of immune dysregulation (7%, Germany: 6%), predominantly antibody disorders (20%, Germany: 57%), other well-defined PIDs (25%, Germany: 11%) (7). However, more autosomal recessive diseases are present in Kuwait, due to a higher number of consanguineous marriages. The South African Primary Immunodeficiency Registry (SAPID) with its 301 registered patients, uses also six categories: antibody deficiencies (45%), complemenrt deficiencies (29%), combinded ID (CD) and phagocyte defect (both 6%), syndromes (1,6%), autoinflammatory (3%), innate defects (2%), unclassified ID (defined as patients “with a clinical profile of immune deficit but without any laboratory-demonstrated defect” (1%), and dysregulation (0.7%) (14).

Moreover, it is unclear whether all publications are based on the same version of IUIS classification.

The classification of PID is a dynamic process that changes regularly due to ongoing research. The ESID classification is based on the IUIS classification. However, since IUIS publishes a new version of the PID classification every two years, it is continuous work in progress to align the ESID classification with the IUIS. We try to keep up with the modifications of the IUIS classification but the actual implementation in the ESID registry naturally lags behind. The current classification presented in this manuscript therefore has no one to one representation in an IUIS classification. For example, the PID-NET registry followed a suggestion by the IUIS to rename the main categories, whereby “predominantly T-cell deficiencies” were subordinated under “combined immunodeficiencies,” and “autoimmune and immune dysregulation syndromes” are now listed as “diseases of immune dysregulation.” Furthermore, the 2014 re-design of the registry with the introduction of clinical diagnostic working criteria led, in some cases, to changes in PID diagnoses; thus, the current distribution of main categories cannot be directly compared to those published before the re-design (18).

### PID Distribution

The distribution of PID regarding to the year of diagnosis was influenced by the first published description of the respective PID and the discovery of the disease-causing mutation. For example, the first report of A-T was in 1926, but cloning and identification of the *ATM* gene was only published in 1995 (19). The DGS causing microdeletion *Del 22q11.2* was found 1981. The genes defects causing HIES were only identified in 2007 (*STAT3) and* 2009 (*DOCK8*). A specific PID diagnosis can only be assigned when the PID was described and published before. With the introduction of the new diagnostic ESID-registry criteria for PID, the PID-NET registry has now implemented a more uniform classification system for PID, leading to more consistency in the registration of diagnoses.

### Genetic Testing

To date, there is no system in place that offers regular, widespread genetic testing in Germany. Genetic tests were not performed in most of the CVID patients, unclassified PIDs, isolated IgG subclass deficiency, and selective IgA deficiency (Supplementary Table 34). This might be due to the fact that the patient's PID diagnosis was considered to be already clear to the physician based on clinical and laboratory results and therefore a genetic confirmation of the PID diagnosis will not have an effect of the patient's further treatment. Another reason for not testing everybody are the still high costs. Genetic analysis was performed in 168 patients by targeted gene sequencing, but the suspected disease-causing mutation was not found. However, the proportion of PID patients who do have a genetically-confirmed diagnosis is increasing: whereas only about 31.2% of all patients in the PID-NET registry had a genetic diagnosis in March 2012 (4), this number increased to 36.4% by July 2017. Other registries report a similar percentage of genetically-confirmed diagnoses [France: 40% (17), Russia: 36% (20), Iran 33.1% (12)]; in the Kuwait registry, a PID-causing mutation was found in 53% of the 264 patients (21). This indicates that genetic testing is becoming an increasingly important component in the work-up of PID patients. But who should be tested? For example, at the PID reference centre in Freiburg, all patients with a suspected PID undergo genetic testing, given that the management plan always changes with a genetically-confirmed diagnosis.

### Presenting Symptoms

While 74% of all PID patients with presenting symptoms had infections, a substantial proportion of patients (25%) presented with immune dysregulation. Therefore, PID should be suspected in any patient with immune dysregulation. The ranking of presenting symptoms in the South African registry (ASID) was similar with regard to infections as most prevalent symptom, followed by autoimmunity features (14). However, due to a different classification, the precise numbers cannot be directly compared. The Iranian registry classified the presenting symptoms based on the affected organ (12). Thus, a direct comparison is again difficult. However, infections are frequently presented among the listed entities.

### Age Distribution

In the most recent report of the UKPID registry, 17% of patients (807) were 16 years or younger (11), whereas in Germany the number of registered PID patients aged 16 or under was 42% (947 patients). In 2014, the proportion of patients in the Swiss National Registry for PID who were younger than 18 was 31% (109 living patients from 348 patients) (9), in the US-American USIDNET it was 25% out of 3,459 patients (6) whereas in Germany, the corresponding percentage was 45% (1,010 living patients). This indicates that in the PID-NET registry, paediatric patients are considerably more strongly represented in comparison to the British, Swiss and US-American registries. This might also be due to the fact, that there are only a few centres in Germany which focus on treating adult PID patients.

### Diagnostic Delay

In Germany, there are the guidelines of API (Arbeitsgemeinschaft Pädiatrische Immunologie e.V.) and DGfI (Deutschen Gesellschaft für Immunologie) for the diagnosis of PID^9^. However, the registry does not ask for the fulfilment of theses formal criteria of the pathological susceptibility to infections, but the first clinical symptoms suggestive of a PID as based on the physician's judgement.

Comparison of the diagnostic delay with other registries can be difficult, since the exact definition of “diagnostic delay” is missing (11) or is different to the criteria used in this publication. The Kuwait registry defines diagnostic delay as time between the “initial presentation” of the patient and the definitive PID diagnosis (8), whereas CEREDIH uses the timespan between birth and diagnosis, resulting in a median diagnostic delay of 6 years in CVID and 1 year in agammaglobulinemia (17). The median diagnostic delay for CVID patients in Switzerland was also 6 years (9), whereas in the UK it was 4 years (11), and in Germany 3 years. The median diagnostic delay for agammaglobulinemia patients was 1 year in the UK (11) and <1 year in Germany.

The reason why some PID patients only manifest late in their lives may either lie in the variable expressivity of their respective mutation (e.g., as seen in *ADA1*), in the fact that a PID-predisposing condition needs to be triggered by a specific infection (e.g., EBV in XLP), or that the PID is not primarily monogenetic but has an epigenetic component (22).

### IgG Substitution

The median IgG dosage for CVID patients in Sweden (90 patients) and Germany (568 patients) was similar: 500 mg per kg body weight per month in Sweden (23) and 481 mg per kg body weight per month in the PID-NET registry.

According to the Swedish PIDcare registry, of the 566 patients who had received IgG substitution by the end of September 2017, 9.4% were treated intravenously, 84.1% subcutaneously, and 6.5% intramuscularly^10^. This compares to 29% intravenously and 70% subcutaneously in Germany. Other registries did not report the relative proportions of sc vs. iv therapy.

Documentation of severe side effects due to IgG substitution was rarely observed. In Germany, 5 out of the 128 patients (11%) with side effects had anaphylaxis. There may also be a bias in documentation due to way the physician communicates with the patient; for example, if the physician states that a little swelling or induration is normal, the patient might not report it as a side effect at all. Other registries did not report any side effects from immunoglobulin replacement.

## Conclusion and Outlook

The medical care of PID in Germany differs in contrast to some other countries in such as there is no real nationwide coverage of medical care in the field of clinical immunology. The medical care for adults are restricted to only very few centres whereas for patients under 18 years (with a mainly genetically diagnosed PID), the medical care takes place in many children hospitals. The German PID-NET registry is a precious tool for physicians, researchers, the pharmaceutical industry, politicians, and ultimately the patients, for whom the new medical and political outcomes will eventually lead to better understanding in PID, a more timely diagnosis and better treatment.

In order to make the registry even more powerful, we suggest addressing the following items for improving the data entry process at hospitals: motivation program for physicians and study nurses, incentives for participation (e.g., own statistics), additional staff for research purposes, closer collaboration between participating and not yet participating departments within and between hospitals, as well as with potential financial backers. Additional personnel (e.g., an additional documenting specialist to support the growing number of centres) would also provide a significant level of help in fostering the documenting process. Furthermore, the implementation of additional departments that monitor PID patients should be promoted. In addition to these general measures, another aim is to extend the Level 1 dataset covered by the registry by providing more detailed information on malignancies and the use of biologicals, and entering additional laboratory values. However, substantial long-term funding is ultimately needed in order to achieve these aims.

## Data Availability

The datasets for this manuscript are not publicly available due to the respective agreements with the documenting centres. Requests to access the datasets should be directed to the PID-NET registry, e.g. via the corresponding authors.

## Ethics Statement

This study was approved by the ethics committee of the University Hospital Freiburg for the ESID registry (approval no. ESID registry: 493/14). All patients or their legal guardians gave written informed consent for participation in the registry.

## Author Contributions

All authors contributed to the management and documentation of patients and gave their final approval to the submitted version. SE-H, BG, GK, and VS performed data analysis and wrote the manuscript.

### Conflict of Interest Statement

ES received speaker's fees, travel grants, research funding, or compensation for consultancies from Roche, Chugai, MSD, Baxalta (Shire/Takeda), CSL Behring, AbbVie, Janssen, Novartis. MF recieved honoraria for Advisory-Board-Meetings/lectures from Shire, CSL Behring; travel grants from Octapharma. KW has received grants in the last five years by the BMBF, DFG, CSL Behring, Biotest, Bristol-Myers-Squibb unrelated to this publication. He also has received honoraria from Takeda/Shire/Baxalta/Baxter, CSL-Behring, Biotest, Octapharma and Pfizer. PTO received honoraria from Novartis. SB reports Advisory Board Membership / Consultancy for Bayer, Celgene, Chugai, Clinigen, Ipsen, Isofol, Lilly, Merck & Co., Novartis, Pfizer, Roche, Sensorion, and Takeda Millenium outside of the submitted work. CB received honoraria for consultancies/lectures from AbbVie, Gilead, Janssen, MSD, ViiV. Research funding by Deutsche Leberstiftung, DZIF, Hector Stiftung, NEAT ID. UHB received grants from the BMBF and the European Commission, and consulting fees from Biotest, CSL Behring, octapharma, and Shire. SE has received consultation fees from UCB, Novartis, Sobi, and research support from UCB. SE-H, during the conduct of this 7-year study, was funded by the Federal Ministry of Education and Research (BMBF), by the European Society for Immunodeficiencies (ESID), by the Care-for-Rare Foundation, by PROimmun e.V., and by a restricted grant from LFB. BG has obtained a large grant from the BMBF (2009-2018). Unrelated to this research, up until 2018 he received research funding from the European Commission (EC), the Deutsche Forschungsgemeinschaft (DFG), the BMBF, and the companies CSL-Behring, Baxalta, Bristol-Myers-Squibbs and Novartis. In the last 7 years, he also received consultancy fees from the following companies: Novartis, Janssen-Cilag, Shire/Baxalta/Baxter, Kedrion, CSL-Behring, and Biotest. AJ has received a research grant by Baxalta. GK was partially funded by grants from the BMBF and ESID for the purpose of this research. TN receives travel reimbursements of the European Medicines Agency (EMA), the Pediatric Rheumatology JIR cohort (Lausanne, Switzerland), the Jeffrey Modell Foundation for Primary Immunodeficiencies (New York, USA) and the PENTA group (Pediatric European Network Trial in AIDS) and author honoraria from uptodate.com (Waltham, Massachusetts, USA). CMS reports research grant and speaker honoraria from Octapharma; clinical study grant and speaker honoraria from Baxalta/Shire; research grant from CSL Behring; and clinical study grant and speaker honoraria from Fresenius. She is a member in Advisory board of Octapharma, Baxalta/Shire. ES reports personal fees from Chugai, AbbVie, Janssen Cilag, Celgene, Eli Lilly, Shire (Takeda), and Novartis. MTS received speaker's fees, travel grants, research funding, or compensation for consultancies or board memberships from AbbVie, Actelion, BMS, Celgene, Chugai/Roche, Genzyme, Hexal/Sandoz, Janssen-Cilag, MSD, Novartis, Pfizer, Sanofi Pasteur, Shire (Baxalta), UCB. H-PT reports personal fees from Abbvie, Chugai, Janssen Cilag, Eli Lilly, Novartis, Roche, Sandoz Hexal, Sanofi Aventis, and Shire (Takeda). Data of the PID-NET registry were stored and processed on the ESID registry platform. The ESID registry was supported in the years 2012-2017 by the PPTA (Plasma Protein Therapeutics Association, https://www.pptaglobal.org), Baxter, Novartis, GSK and UCB. The PID-NET registry was funded by BMBF. The publication fee of this article was covered by Deutsche Forschungsgemeinschaft under Germany's Excellence Strategy - EXC 2155 RESIST - Project ID 39087428. The remaining authors declare that the research was conducted in the absence of any commercial or financial relationships that could be construed as a potential conflict of interest.

## Footnotes

^1^ESID. *ESID Registry – Working Definitions for Clinical Diagnosis of PID*. Available online at: https://esid.org/Working-Parties/Registry/Diagnosis-criteria (accessed August 8, 2017).

^2^ESID. *ESID Registry – Working Definitions for Clinical Diagnosis of PID*. Available online at: https://esid.org/Working-Parties/Registry/Diagnosis-criteria (accessed August 8, 2017).

^3^ESID. *ESID Online Registry: Diseases and Genes*. Available online at: https://esid.org/Working-Parties/Registry/New-ESID-Registry/List-of-diseases-and-genes (accessed August 8, 2017).

^4^ESID. *Registry Working Party*. New ESID questionnaires. Available online at: https://esid.org/Working-Parties/Registry/New-ESID-Registry/New-ESID-questionnaires (accessed August 8, 2017).

^5^ESID. *ESID Registry – Working Definitions for Clinical Diagnosis of PID*. Available online at: https://esid.org/Working-Parties/Registry/Diagnosis-criteria (accessed August 8, 2017).

^6^Statistisches Bundesamt (Destatis). *Bevölkerung auf Grundlage des Zensus 2011*. Available online at: https://www.destatis.de/DE/ZahlenFakten/GesellschaftStaat/Bevoelkerung/Bevoelkerungsstand/Tabellen/Zensus_Geschlecht_Staatsangehoerigkeit.html (accessed December 5, 2018), German, English

^7^USIDNET. *Core Report Form*.. Available online at: https://usidnet.org/about-the-registry/registry-user-resources/data-collection-forms/ and https://usidnet.org/wp-content/uploads/2014/01/Paper-Registry-2017-Update.pdf (accessed August 14, 2018).

^8^*Statistisches Bundesamt (Destatis): Bevölkerung: Deutschland, Stichtag, Altersjahre, Bevölkerung auf Grundlage des Zensus 2011*. Available online at: https://www-genesis.destatis.de/genesis/online/link/tabelleErgebnis/12411-0005/ (accessed July 5, 2017), German.

^9^Susan Farmand, Ulrich Baumann, Horst von Bernuth, Michael Borte et al. *Leitlinie: Diagnostik auf Vorliegen eines primären Immundefekts*. AWMF. Available online at: https://www.awmf.org/leitlinien/detail/ll/112-001.html (accessed March 7, 2019), German.

^10^PIDcare. *Resultat och statistik sedan registret startades*. Available online at: http://pidcare.se/wp-content/uploads/2017/07/Redovisning-kvartal-3.pdf (accessed November 19, 2018), Swedish.

## Abbreviations

AI: autoimmune disease
ALPS: autoimmune lymphoproliferative syndrome
APDS: activated PI3K-delta syndrome
APECED: autoimmune polyendocrinopathy candidiasis ectodermal dystrophy
A-T: ataxia telangiectasia
ATM: ataxia telangiectasia mutated
CEREDIH: Centre de Référence Déficits Immunitaires Héréditaires
CID: combined immunodeficiency
CGD: chronic granulomatous disease
CINCA syndrome: chronic infantile neurologic cutaneous and articular syndrome
CMC: chronic mucocutaneous candidiasis
combined ID: combined immunodeficiency
CSR/HIGM: Class-Switch Recombination defects/Hyper IgM (HIGM) syndromes
CVID: common variable immunodeficiency
def.: deficiency
DGS: DiGeorge syndrome
EBMT registry: European Society for Blood and Marrow Transplantation Registry
ESID: European Society for Immunodeficiencies
FCAS: familial cold autoinflammatory syndrome
FHLH: familial hemophagocytic lymphohistiocytosis syndromes
FMF: familial Mediterranean fever
G6PD: glucose-6-phosphate dehydrogenase deficiency
GSD 1b: glycogen storage disease type 1b
HAE (*C1inh*): hereditary angioedema (C1 inhibitor)
HIES: hyper IgE syndrome
HLA: human leukocyte antigen
HSCT: hematopoietic stem cell transplantation
IBD: inflammatory bowel disease
ICF: immunodeficiency centromeric instability facial anomalies
ID: immunodeficiency
IgA: immunoglobulin A
IgG: immunoglobulin G
IgM: immunoglobulin M
innate ID: defects in innate immunity
IPEX: immunodysregulation polyendocrinopathy enteropathy X-linked syndrome
iv: intravenous
LAD: leukocyte adhesion deficiency
MBL: mannose-binding lectin
MSMD: mendelian susceptibility to mycobacterial disease
MonoMAC: monocytopenia and mycobacterial infection
NBS: Nijmegen breakage syndrome
PFC: properdin P factor complement deficiency
PID: primary immunodeficiency
PMS2: post-meiotic segregation 2
RALD: Ras-associated lymphoproliferative disease
SCID: severe combined immunodeficiency
SPAD: deficiency of specific IgG (specific antibody deficiency)
sc: subcutaneous
SCETIDE: Stem Cell Transplant for primary Immune Deficiencies in Europe
TLR/NFkappa-B: Defects of Toll-like receptor/nuclear factor of kappa light polypeptide gene enhancer in B cells 1
TRAPS: TNF-receptor associated periodic fever syndrome
WAS: Wiskott-Aldrich syndrome
WHIM: warts hypogammaglobulinemia infections and myelokathexis
XLP: X-linked lymphoproliferative syndrome
XLT (*WASP*): X-linked thrombocytopenia with mutations in *WASP*
WASP: Wiskott-Aldrich syndrome protein.
