# Supplementary material for: The German National Registry of Primary Immunodeficiencies (2012–2017)
Source: Front Immunol. 2019 Jul 19;10:1272. doi: 10.3389/fimmu.2019.01272 (PMC6659583; doi:10.3389/fimmu.2019.01272)
Supplement: Supplementary file 1 [file Presentation_1.pdf]

Supplements

Table of Content

S1: Documenting centres 4
S2: ESID Online Registry: List of diseases and genes with registered patients. 16
S3: Genetic delay in 24 SCID patients 17
S4: Genetic delay in 37 HIES patients 17
S5: Registered PID-distribution in Germany, March 2012. 18
S6: The 12 foremost PID and genes within their main category 20
S7: Main categories of PID: Differences in grouping main categories (2012 and 2017) 21
S8: Age and gender distribution of 1,437 living patients of the 12 PIDs patients groups, XLP, WAS, and FHLH patients (no HSCT or gene therapy) 22
S9: Age and gender distribution of 208 living patients of the 12 PIDs patients groups, XLP, WAS, and FHLH patients (with HSCT, and 3 patients also with gene therapy) 22
S10: Age range and median age at last news from patient in years of the 12 most prevalent PID diagnoses and WAS, XLP and FHLH (patients with HSCT but no gene therapy), living patients 23
S11: Age at gene therapy 23
S12: Age and gender distribution of 665 living CVID patients. No patients received an HSCT or gene therapy. 24
S13: Age and gender distribution of 221 living unclassified antibody deficiency patients. No patients received an HSCT or gene therapy. 24
S14: Age and gender distribution of 8 living SCID patients. No patients received an HSCT or gene therapy. 25
S15: Age and gender distribution of 64 living SCID patients (with HSCT, no gene therapy). 25
S16: Age and gender distribution of 52 living DGS patients (with HSCT, no gene therapy). 26
S17: Age and gender distribution of 82 living isolated IgG subclass deficiency patients. No patients received an HSCT or gene therapy. 26
S18: Age and gender distribution of 42 living unclassified IDs. No patients received an HSCT or gene therapy. 27
S19: Age and gender distribution of 47 living selective IgA deficiency patients. No patients received an HSCT or gene therapy. 27
S20: Age and gender distribution of 58 living CGD patients. No patients received an HSCT or gene therapy. 28
S21: Age and gender distribution of 51 living CGD patients (HSCT but no gene therapy) 28
S22: Age and gender distribution of 97 living agammaglobulinemia. No patients received an HSCT or gene therapy. 29
S23: Age and gender distribution of 38 living combined ID. No patients received an HSCT or gene therapy. 29
S24: Age and gender distribution of 25 living combined ID patients (HSCT but no gene therapy) 30
S25: Age and gender distribution of 47 living HIES patients. No patients received an HSCT or gene therapy. 30
S26: Age and gender distribution of 8 living HIES patients (HSCT but no gene therapy) 31
S27: Age and gender distribution of 55 living A-T patients. No patients received an HSCT or gene therapy. 31
S28: Age-and gender distribution of 21 male patients receiving HSCT and/or gene therapy. 32
S29: Age-and gender distribution of 18 living WAS patients (HSCT but no gene therapy) 32
S30: Age-and gender distribution of 15 living XLP patients. No patients received an HSCT or gene therapy. 33

S31: Age-and gender distribution of 7 living XLP patients (HSCT but no gene therapy) 33
S32: Age-and gender distribution of 3 living FHLH patients. No patients received an HSCT or gene therapy. 34
S33: Age-and gender distribution of 15 living FHLH patients (HSCT but no gene therapy) 34
S34: PIDs with nearly no genetic information 35
S35: 12 PIDs: genetic information 36
S36: Mainly male and female patients in PID with non-X-linked gene defect, with a diagnosed gene defect 37
S37: Consanguinity and family case in main categories 37
S38: Gender, deceased, lost, gene identified, consanguinity, and family case by PIDs 45
S39: Presenting symptoms 49
S40: Onset of the 12 most common PIDs 50
S41: Onset of symptoms mainly in the first year of life 51
S42: Onset of symptoms mainly between the ages of 1 to 5 51
S43: Onset of symptoms with main onset age of 1 to 5 years and late onset 51
S44: Genetic diagnosis before onset of symptoms 52
S45: Age at diagnosis in 2,314 patients 53
S46: Average and median of genetic diagnostic delay 54
S47: Cause of death for 51 patients 58
S48: PID with no IgG substitution (PIDs with minimum 10 patients of 270 patients) 58
S49: IgG substitution delay 63
S50: Relative IgG-dose [mg per kg body weight per month] and side effects (1,061 patients) 65
S51: Patients with HSCT 67
S52: Patients with HSCT and Ig treatment after HSCT 68
Age distribution by year of birth 69
    S53: Age distribution by year of birth of 2,453 patients 69
    S54: Age distribution by year of birth of 12 PIDs (1,825 patients) 69
    S55: Age distribution by year of birth of 728 CVID patients 70
    S56: Age distribution by year of birth of the 5 PIDs with the most patients (all registered patients) 70
    S57: Age distribution by year of birth of the first 7-12 PIDs with the most patients (all registered patients) 71

State of Germany	Centre	Number of patients 2012	Number of patients 2017
Baden-Württemberg		512	526
	Freiburg, CCI (c.d. / a.d.*)	508	488
	Ulm, University Children's Hospital	3	79
	Tübingen, University Hospital (c.d. / a.d.)	0	22
	Heidelberg, Children's Hospital (Immunology)	0	9
	Stuttgart, Klinikum Stuttgart – Olgahospital (c.d.)	4	7
Bavaria		221	485
	Munich, LMU: Children's Hospital Dr. von Hauner	191	309
	Würzburg, University Hospital (c.d. / a.d.)	27	59
	Munich, TUM, Children Hospital Schwabing	0	30
	Erlangen, University Children's Hospital	0	8
Berlin		33	326
	Berlin, Charité (c.d. / a.d.)	33	322
	Berlin, Vivantes Klinikum im Friedrichshain (c.d.)	0	3
	Berlin-Buch, Helios Hospital (c.d.)	0	1
Bremen		5	11
	Bremen, Prof.-Hess-Kinderklinik	5	11
Hamburg		0	7
	Hamburg-Eppendorf, Paediatric SCT and Immunology	0	7
Hessen		59	103
	Frankfurt, University Hospital	59	103
Lower Saxony		270	388
	Hannover, MHH (c.d. / a.d.)	270	384
	Göttingen, UMG: Centre for Child and Adolescent Health	0	4
Mecklenburg-Vorpommern		4	5
	Rostock, University Children's Hospital	4	5
North Rhine-Westphalia		177	282
	Düsseldorf, University Children's Hospital	57	86
	Krefeld ID Centre	47	77
	Münster, University Hospital	21	44
	Bochum, University Children's Hospital	37	30
	Bonn, University Hospital (c.d. / a.d.)	14	22
	Sankt Augustin, Asklepios Klinik	1	7
	Aachen, Children's University Hospital	0	5
	Dortmund, KlinikumDo, Childrens Hospital	0	5
	Cologne, Kinderkrankenhaus Amsterdamer Strasse	0	4
	Essen, St. Josef Krankenhaus Essen-Werden GmbH (a.d.)	0	2
Saarland		0	8
	Homburg, University Children's Hospital	0	8

State of Germany	Centre	Number of patients 2012	Number of patients 2017
Saxony		43	225
	Leipzig, Children's hospital St. Georg	0	184
	Leipzig, MVZ Dr. Reising-Ackermann u. Kollegen (a.d.)	62	67
	Dresden, University Children's Hospital	43	41
	Leipzig, University Children's Hospital	0	0
Saxony-Anhalt		62	68
	Halle, University Children's Hospital	0	1
	Magdeburg, University Children's Hospital	0	0
Schleswig-Holstein		0	19
	Kiel, UKSH (c.d. / a.d.)	0	19
**Total:**		**1,386**	**2,453**

S1: Documenting centres

*c.d.: children department; a.d.: adult department

German centres which entered data into PID-NET registry. We grouped the centres by federal states (alphabetically) and cities (according to the amount of registered patients). Magdeburg and university hospital of Leipzig did not enter any data yet

Main category	Sub category	PID diagnosis	*Mutated gene**	Number of patients
Autoinflammatory disorders (84 patients)	Blau syndrome	Caspase recruitment domain-containing protein 15 deficiency (CARD15)	*CARD15*	0
	CINCA syndrome	CINCA syndrome	*CIAS1 (NLRP3)*	6
	Familial cold autoinflammatory syndrome	Familial cold autoinflammatory syndrome	*CIAS1 (NLRP3)*	2
	Familial Mediterranean fever	Familial Mediterranean fever defect	*MEFV*	24
	Familial periodic fever	Hyper IgD syndrome (MVK)	*MVK*	5
		TNF-receptor associated periodic fever syndrome (TRAPS)	*TNFRSF1A*	18
	Muckle-Wells syndrome	Muckle-Wells syndrome	*CIAS1 (NLRP3)*	7
	Other autoinflammatory diseases with known genetic defect	Other autoinflammatory diseases with known genetic defect	*CAD14*	0
			HOIL1	0
			IL1RN	0
			IL36RN	0
			LPIN2	0
			PLCG2	0
			PSMB8	0
			SH3BP2	0
			SLC29A3	0
	Periodic fever, unclassified	Periodic fever, unclassified	none	0
	Periodic fever aphtous stomatitis, pharyngitis and adenopathy (PFAPA)	PFAPA	none	0
	Pyogenic sterile arthritis pyoderma gangrenosum and acne	Proline/serine/threonine phosphatase-interacting protein 1 deficiency (PSTPIP1)	*PSTPIP1*	0
	Unclassified autoinflammatory diseases	Unclassified autoinflammatory diseases	none	22
Combined immunodeficiencies (177 patients)	Atypical Severe Combined Immunodeficiency (Atypical SCID)	Atypical Severe Combined Immunodeficiency (Atypical SCID)	*ADA*	1
			Artemis	1
			CARD11	0
			CD3d	0
			CD3e	0
			CD3z	0
			Coronin-1A	1
			IL2RG (X-linked)	0
			IL21R	0
			IL7Ralpha	0
			JAK3	0
			PNP	2
			RAG1	1
			RAG2	1

Main category	Sub category	PID diagnosis	*Mutated gene**	Number of patients
	CD4-deficiency	Selective CD4 cell deficiency	*CD4*	1
			none	1
	CD8-deficiency	CD8 deficiency	*CD8A*	0
	Combined immunodeficiency (CID)	Combined immunodeficiency (CID)	*Artemis*	2
			CARD11	0
			Caspase 8	0
			CD3G	0
			CD27	0
			CD70	0
			Cernunnos	3
			CTLA-4	4
			DNA-PKcs	0
			DOCK8	5
			IKBKB	0
			IL21R	0
			IL2Ralpha	0
			ITK	1
			LCK	0
			LIG4	2
			LRBA	1
			MAGT1	0
			MALT1	0
			MSN (moesin)	0
			MST1 (STK4)	0
			Orai1 (TMEM142A)	2
			OX40	0
			PGM3	0
			PIK3CD (PI3K-delta)	4
			PIK3R1	0
			PNP	6
			PRKDC	2
			RECQL4 (Poikilodermia congenita)	0
			RhoH	0
			RLTPR	0
			STAT1	0
			STAT5	0
			STAT5a	0

Main category	Sub category	PID diagnosis	*Mutated gene**	Number of patients
			STAT5b	0
			STIM1	1
			TRAC	0
			TTC7A	0
			UNC119D	0
			ZAP70	0
			none	41
		Activated PI3K-delta syndrome (APDS)	*PIK3R1 (PI3K-delta)*	0
			PIK3R1	0
	HLA class I deficiency	HLA class I deficiency	*TAP1*	0
			TAP2	0
			TAPBP	0
	HLA class II deficiency	HLA class II deficiency	*MHC2TA*	1
			RFX5	0
			RFXANK	1
			RFXAP	2
			none	1
	NUDE/SCID	Winged-helix nude deficiency (FOXN1)	*FOXN1*	0
	Omenn syndrome	Omenn syndrome	*ADA*	0
			Artemis	0
			CD45	0
			Coronin-1A	0
			Del 22q11.2	0
			IL2RG (X-linked)	0
			IL21R	0
			IL2Ralpha	0
			IL7Ralpha	0
			JAK3	0
			RAG1	1
			RAG2	1
			RMRP	0
			none	3
	Severe combined immunodeficiency (SCID)	Reticular Dysgenesis - AK2 (SCID)	*AK2*	1
		Reticular Dysgenesis - UNK (SCID)	none	0
		Severe combined immunodeficiency (SCID)	*ADA*	10
			Artemis	11
			CD3d	0
			CD3e	0
			CD3z	0

Main category	Sub category	PID diagnosis	*Mutated gene**	Number of patients
			CD45	0
			Coronin-1A	0
			Del 22q11.2	0
			DNA-PKcs	0
			IL2RG (X-linked)	18
			IL21R	0
			IL7Ralpha	3
			JAK3	2
			LIG4	0
			RAG1	14
			RAG2	6
			none	19
Complement deficiencies (44 patients)	Complement deficiency	Acquired angioedema	none	1
		C3b inactivator deficiency	*C3b inactivator*	0
		CD59 antigen P18-20 deficiency (CD59)	*CD59*	0
		Complement component 1 deficiency	*C1Q-alpha*	0
			C1Q-beta	1
			C1Q-gamma	0
			C1r	0
			C1s	0
			Q, subunit unknown	0
		Complement component 2 deficiency	*C2*	6
		Complement component 3 deficiency	*C3*	0
		Complement component 4 deficiency	*C4*	0
		Complement component 5 deficiency	*C5*	0
		Complement component 6 deficiency	*C6*	0
		Complement component 7 deficiency	*C7*	1
		Complement component 8 deficiency	*C8*	3
		Complement component 9 deficiency	*C9*	0
		Complement factor B deficiency	*Factor B*	0
		Complement factor H deficiency	*Factor H*	0
		Decay-accelerating factor for complement deficiency (DAF CD55)	*CD55*	0
		Factor D deficiency	*Factor D*	0
		Factor I deficiency	*Factor I*	1
		Ficolin3 deficiency	*FCN3*	0
		Hereditary Angioedema (C1inh)	*C1 Inhibitor*	20
			none	3

Main category	Sub category	PID diagnosis	*Mutated gene**	Number of patients
		Mannan-binding lectin serine protease (MASP) deficiency	*COLEC11*	0
			MASP1	0
			MASP2	0
		Membrane Attack Complex Inhibitor (CD59) deficiency	*CD59*	0
		Membrane Cofactor Protein (CD46) deficiency	*CD46*	0
		Properdin P factor complement deficiency (PFC)	*Properdin*	1
			none	1
		Thrombomodulin deficiency	*THBD*	0
	Mannose-binding lectin (MBL)	Mannose-binding lectin deficiency (MBL)	*MBL*	1
	Unclassified complement deficiencies	Unclassified complement deficiencies	none	5
Defects in innate immunity (45 patients)	CARD 9 deficiency	CARD9 deficiency	*CARD9*	0
	Chronic mucocutaneous candidiasis (CMC)	Chronic mucocutaneous candidiasis (CMC)	*ACT1*	0
			AIRE	0
			IL-17 F	1
			IL-17 receptor alpha	1
			STAT1	13
			none	4
	Defects of TLR/NFkappa-B signalling	Defects of TLR/NFkappa-B signalling	*IKK-gamma (NEMO, IKBKG)*	3
			IRAK4	3
			MyD88	3
			NFKBIA	0
			TIRAP	0
	Epidermodysplasia verruciformis	Epidermodysplasia verruciformis	*TMC6 (gene 1)*	0
			TMC8 (gene 2)	0
	Herpetic encephalitis	Herpetic encephalitis (HSE)	*TBK1*	0
			TLR3	0
			TRAF3	0
			TRIF	0
			UNC93	0
	HOIL1 deficiency	HOIL1 deficiency	none	0
	Predisposition to several viral infection	Predisposition to several viral infection	*MCM4*	0
			STAT2	0
	Trypanosomias	Trypanosomias	*APOL-I*	0
	Unclassified defects in innate immunity	Unclassified defects in innate immunity	none	12
	Warts hypogammaglobulinemia infections and myelokathexis (WHIM)	Warts hypogammaglobulinemia infections and myelokathexis (WHIM)	*CXCR4*	5

Main category	Sub category	PID diagnosis	*Mutated gene**	Number of patients
Diseases of immune dysregulation (136 patients)	Autoimmune lymphoproliferative syndrome (ALPS)	Autoimmune lymphoproliferative syndrome (ALPS)	*CARD11*	0
			Caspase 10 (ALPS IIA)	0
			Caspase 8 (ALPS IIB)	0
			CD95 (germline - ALPS IA)	18
			CD95 (somatic - ALPS Im)	5
			FAS (TNFRSF6)	8
			FASL (CD178) (ALPS IB)	3
			PRKCD	0
			none	4
		ALPS-like disease	*LRBA*	0
			FADD	0
		Ras associated lymphoproliferative disease (RALD)	*K-Ras*	1
			N-Ras	0
	Autoimmune polyendocrinopathy candidiasis ectodermal dystrophy (APECED)	Autoimmune polyendocrinopathy candidiasis ectodermal dystrophy (APECED)	*AIRE*	4
			ITCH	0
	Early-onset inflammatory bowel disease	Early-onset inflammatory bowel disease	*ILR10*	0
			ILR10 receptor alpha chain	3
			ILR10 receptor beta chain	1
			none	1
		Inflammatory Bowel Disease-like (IBD-like)	*LRBA*	1
	Early-onset multi-organ autoimmune disease	Early-onset multi-organ AI	*CTLA-4*	0
			STAT3-GOF	2
	Hemophagocytic lymphohistiocytosis (HLH)	CD27 deficiency	*CD27*	1
		CD70-deficiency	*CD70*	0
		Chediak Higashi syndrome	*LYST*	3
			none	1
		Familial hemophagocytic lymphohistiocytosis syndromes (FHLH)	*PRF1*	6
			STX11	0
			STXBP2 (Munc 18-2)	5
			UNC13D	5
			none	4

Main category	Sub category	PID diagnosis	*Mutated gene**	Number of patients
		Griscelli syndrome type 2	*RAB27A*	2
			none	1
		Hermansky-Pudlak syndrome	*AP3B1*	1
		ITK deficiency (HLH phenotype)	*ITK*	0
		X-linked lymphoproliferative syndrome (XLP)	*BIRC4/XIAP (XLP2)*	19
			SH2D1A (XLP1)	10
	IPEX	FOXP3 deficiency (IPEX)	*FOXP3*	5
		Interleukin 2 receptor alpha deficiency (CD25) (IPEX phenotype)	*IL2Ralpha*	1
		IPEX-like disease	*LRBA*	0
	Type 1 interferonopathies	Type 1 interferonopathies	*ACP5*	1
			ADAR1	0
			RNASEH2A	0
			RNASEH2B	0
			RNASEH2C	0
			SAMHD1	0
			TREX1	0
			STING (TMEM173)	0
	Unclassified disorders of immune dysregulation	Unclassified disorders of immune dysregulation	none	20
Other well defined PIDs (283 patients)	Asplenia syndrome	Asplenia syndrome (Ivemark syndrome)	none	4
		Isolated congenital asplenia	*RPSA*	0
			none	2
	Cartilage hair hypoplasia	Cartilage hair hypoplasia	*RMRP*	4
	CHARGE syndrome	CHARGE syndrome	*CHARGE-CHD7*	0
			SEMA3E	0
	Congenital sideroblastic anemia with immunodeficiency, fevers, and developmental delay (SIFD)	Congenital sideroblastic anemia with immunodeficiency, fevers, and developmental delay (SIFD)	*TNRT1*	0
	Defects of Vitamin B12 and Folate metabolism	Defects of Vitamin B12 and Folate metabolism	*MTHFD1*	0
			SLC46A1	0
			TCN2	0
	DiGeorge syndrome	DiGeorge syndrome	*Del 10p*	0
			Del 22q11.2	64
			none	1
	DNA-breakage disorder	AT-like disorder	*MRE11*	0
		Ataxia telangiectasia (A-T)	*ATM*	63
			none	3

Main category	Sub category	PID diagnosis	*Mutated gene**	Number of patients
		Bloom syndrome	*BLM Helicase*	1
			none	0
		DNA-ligase 1 ATP-dependent deficiency (LIG1)	*LIG1*	0
		Immunodeficiency centromeric instability facial anomalies syndrome (ICF)	*DNMT3B*	0
			ZBTB24	1
			none	1
		MCM4 deficiency	*MCM4*	0
		Nijmegen breakage syndrome (NBS1)	*NBS1*	10
		Other DNA-breakage disorder	none	0
		Post-Meiotic Segregation 2 (PMS2) deficiency	*PMS2*	1
		RNF168 deficiency	*RNF168*	0
		Seckel syndrome	none	0
	Dyskeratosis congenita	Dyskeratosis congenita	*DKC1*	2
			NHP2	0
			NOP10	0
			RTEL1	1
			TERC	3
			TERT	0
			TINF2	0
			none	1
		Hoyeraal-Hreidarsson syndrome	*APOLLO (snm1b def)*	0
			DKC1	0
			NHP2	0
			NOP10	0
			RTEL1	0
			TERC	0
			TERT	0
			TINF2	0
	Fc receptor deficiencies	Fc receptor deficiencies	*FCGR1A*	0
			FCGR2A	0
			FCGR2B	0
			FCGR3A	0
			FCGR3B	1
			FCGRT	0
	FILS syndrome	Facial dysmorphism, immunodeficiency, livedo, and short stature (FILS syndrome)	*POLE1*	0

Main category	Sub category	PID diagnosis	*Mutated gene**	Number of patients
	GINS1 deficiency	GINS1 deficiency	*GINS1*	0
	Hyper IgE syndromes	Hyper IgE syndrome (HIES)	*DOCK8*	8
			STAT3	36
			STAT3 DN	2
			Tyk2	0
			none	9
	IKAROS deficiency	IKAROS deficiency	*IKAROS*	0
	Immunodeficiencies with multiple intestinal atresias	Immunodeficiencies with multiple intestinal atresias	*TTC7A*	0
	MonoMAC	Monocytopenia and mycobacterial infection (MonoMAC)	*GATA2*	3
	MYSM1 deficiency	MYSM1 deficiency	*MYSM1*	0
	Netherton syndrome	Netherton syndrome	*SPINK5*	5
	Other syndromic PID	Activated PI3K-delta syndrome (APDS)	*PIK3CD (PI3K-delta)*	2
			PIK3R1	1
	Schimke disease	Schimke disease	*SMARCAL1*	2
	Trichohepatoenteric syndrome (Giraud syndrome)	Trichohepatoenteric syndrome	*SKIV2L*	0
			TTC37	0
	Unclassified syndromic immunodeficiencies	Unclassified syndromic immunodeficiencies	none	16
	VODI	Hepatic venoocclusive disease with immunodeficiency (VODI)	*SP110*	0
	WILD syndrome	Warts, immunodeficiency, lymphedema, dysplasia (WILD) syndrome	none	0
	Wiskott-Aldrich syndrome (WAS)	WIP deficiency	*WIPF1*	0
		Wiskott-Aldrich syndrome (WAS)	*WASP* (X-linked)	24
			none	5
		X-linked thrombocytopenia with mutations in WASP	*WASP* (X-linked)	7
Phagocytic disorders (199 patients)	Actin beta deficiency	Actin beta deficiency (ACTB)	*Actin beta*	0
	Barth syndrome	Barth syndrome	*TAZ*	0
	Chronic granulomatous disease(CGD)	Chronic granulomatous disease (CGD)	*GP91-phox(CYBB)*	80
			P22-phox (CYBA)	7
			P40-phox (NCF4)	0
			P47-phox (NCF1)	24
			P67-phox (NCF2)	1
			none	17
	Clericuzio-type poikiloderma with neutropenia syndrome	Clericuzio-type poikiloderma with neutropenia syndrome	*C16orf57*	0
	COHEN syndrome	COHEN syndrome	*COH1*	0
	Congenital neutropenia	Congenital neutropenia	*CSF3R*	0
			ELA2	7

Main category	Sub category	PID diagnosis	*Mutated gene**	Number of patients
			G6PC3	4
			GATA2	2
			GFI1	0
			HAX1	4
			JAGN1	1
			P14	0
			WASP (X-linked)	2
			none	12
	Cyclic neutropenia	Cyclic neutropenia	*ELA2*	4
	Defects with susceptibility to mycobacterial infection (MSMD)	Defects with susceptibility to mycobacterial infection (MSMD)	*GP91-phox(CYBB)*	0
			IFNGR1	4
			IFNGR2	0
			IL12B	0
			IL12R beta-1	0
			IL18	0
			IL23-alpha	0
			IRAK4	0
			IRF8	0
			ISG15	0
			STAT1	3
			STAT5	0
			none	2
	Glycogen storage disease type 1b (GS1b)	Glycogen storage disease type 1b (GS1b)	*G6PT1*	1
	Leukocyte adhesion deficiency (LAD)	Leukocyte adhesion deficiency (LAD)	*LAD1 / ITGB2*	1
			LAD2 / FUCT1	1
			LAD3	0
	Localized juvenile peridontitis	Localized juvenile peridontitis	*Formyl peptide receptor*	0
	Myeloperoxidase deficiency (MPO)	Myeloperoxidase deficiency (MPO)	*MPO*	0
	Neutrophil glucose-6-phosphate dehydrogenase	Glucose-6-phosphate dehydrogenase deficiency (G6PD)	*G6PD*	1
	Papillon-Lefevre syndrome	Papillon-Lefevre syndrome	*CTSC*	0
	PID with partial albinism	Partial albinism and immunodeficiency syndrome	none	0
	Pulmonary alveolar proteinosis	Pulmonary alveolar proteinosis	*CSF2RA*	0
	RAC2-GTPase defect	RAS-related C3 bolutinum toxin substrate 2 deficiency (RAC2)	*RAC2*	0
	Shwachman-Diamond-syndrome	Shwachman-Diamond-syndrome	*SBDS*	11
	Specific granule defect	CCAAT/enhancer binding protein epsilon deficiency (CEBPE)	*CEBPE*	0

Main category	Sub category	PID diagnosis	*Mutated gene**	Number of patients
	Unclassified phagocytic disorders	Unclassified phagocytic disorders	none	10
Predominantly antibody disorders (1,390 patients)	Agammaglobulinemias	Agammaglobulinemia	*BLNK/SLP65*	0
			BTK (X-linked)	91
			CD79A	1
			CD79B	0
			IGHM	3
			IGLL1	1
			LRRC8	0
			PIK3R1	0
			TCF3	0
			none	16
	Class switch recombination defects (CSR) / HIGM syndromes	CSR defects and Hyper IgM (HIGM) syndromes	*AID*	6
			CD40 (TNFRSF5)	3
			CD40L (CD154)	16
			PMS2	1
			UNG	1
			none	11
	Hypogammaglobulinemias	Common variable immunodeficiency disorders (CVID)	*BAFFR*	1
			CD19	2
			CD20	0
			CD21	0
			CD81	1
			ICOS	2
			LRBA	1
			NFKB1	1
			NFKB2	3
			TACI	10
			TWEAK	0
			none	707
		Deficiency of specific IgG (Specific antibody deficiency - SPAD)	none	18
		IgA with IgG subclass deficiency	*TACI*	0
			none	26
		Immunoglobulin chain deficiencies	*Heavy chain*	0
			Kappa light chain	0
			Lambda light chain	0
		Isolated IgG subclass deficiency	*BAFFR*	0
			CD19	0
			CD21	0

Main category	Sub category	PID diagnosis	*Mutated gene**	Number of patients
			CD81	0
			ICOS	0
			TACI	0
			none	101
		Other immunoglobulin gene deletions	none	0
		Selective IgA deficiency	*TACI*	0
			none	69
		Selective IgM deficiency	none	5
		Thymoma with immunodeficiency	none	2
		Transcobalamin II deficiency	*Transcobalamine II*	0
		Transient hypogammaglobulinemia of infancy	none	21
	Unclassified antibody deficiency	Unclassified antibody deficiency	*11q23*	2
			LRBA	1
			NFKB1	0
			PIK3CD (PI3K-delta)	1
			PRKCD	0
			none	263
		Steinert myotonica dystrophia	*ZNF9*	3
Unclassified immunodeficiencies (76 patients)	Unclassified immunodeficiencies	Unclassified immunodeficiencies	*TACI*	1
			none	75
No PID documented				19
**Total**				**2,453**

S2: ESID Online Registry: List of diseases and genes with registered patients.

* Number of patients with a disease-causing mutation identified in this particular gene.

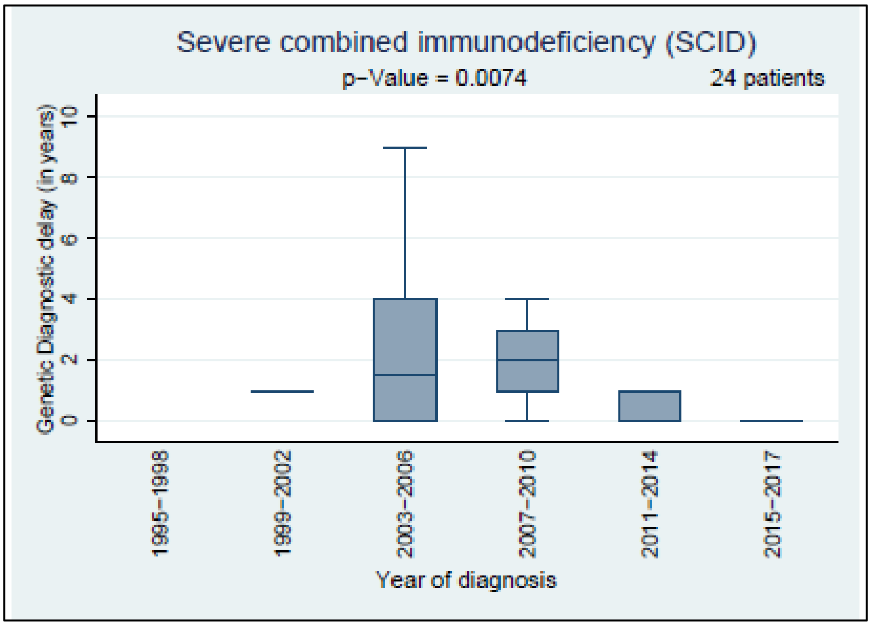

S3: Genetic delay in 24 SCID patients

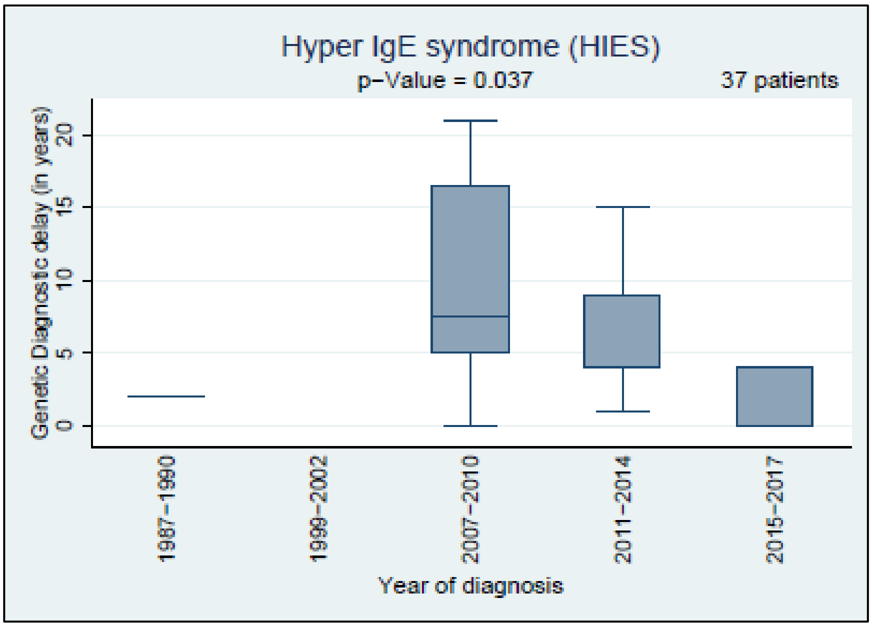

S4: Genetic delay in 37 HIES patients

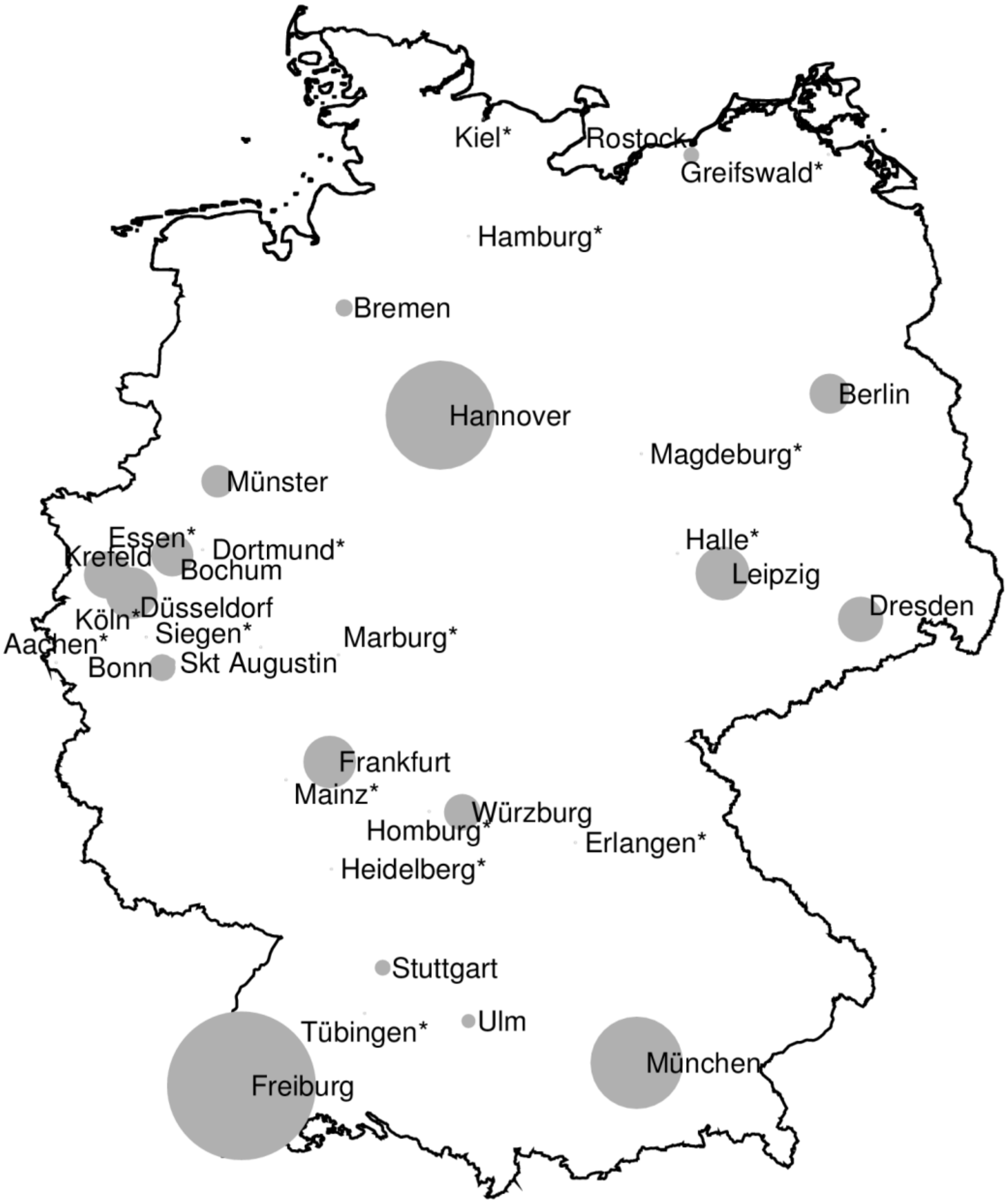

S5: Registered PID-distribution in Germany, March 2012.

Size of the circles is proportional to the numbers of patients. The map is adapted with permission from www.gadm.org. [3]

Main category/PID/Gene	Number of patients	Number of patients [%]
Predominantly antibody disorders	1,39	56,67%
CVID	728	29,68%
(no gene documented)	707	28,82%
TACI	10	0,41%
NFKB2	3	0,12%
CD19	2	0,08%
ICOS	2	0,08%
NFKB1	1	0,04%
BAFFR	1	0,04%
CD81	1	0,04%
LRBA	1	0,04%
Unclassified antibody deficiency	267	10,88%
(no gene documented)	263	10,72%
11q23	2	0,08%
PIK3CD (PI3K-delta)	1	0,04%
LRBA	1	0,04%
Agammaglobulinemia	112	4,57%
BTK (X-linked)	91	3,71%
(no gene documented)	16	0,65%
IGHM	3	0,12%
CD79A	1	0,04%
IGLL1	1	0,04%
Isolated IgG subclass deficiency	101	4,12%
(no gene documented)	101	4,12%
Selective IgA deficiency	69	2,81%
(no gene documented)	69	2,81%
Other well defined PIDs	283	11,54%
A-T	66	2,69%
ATM	63	2,57%
(no gene documented)	3	0,12%
DGS	65	2,65%
Del 22q11.2	64	2,61%
(no gene documented)	1	0,04%
HIES	55	2,24%
STAT3	36	1,47%
(no gene documented)	9	0,37%
DOCK8	8	0,33%
STAT3-DN	2	0,08%

Main Category/PID/Gene	Number of patients	Number of patients [%]
Phagocytic disorders	199	8,11%
CGD	129	5,26%
GP91-phox(CYBB)	80	3,26%
P47-phox (NCF1)	24	0,98%
(no gene documented)	17	0,69%
P22-phox (CYBA)	7	0,29%
P67-phox (NCF2)	1	0,04%
Combined immunodeficiencies	177	7,26%
SCID	83	3,42%
(no gene documented)	19	0,82%
IL2RG (X-linked)	18	0,73%
RAG1	14	0,57%
Artemis	11	0,45%
ADA	10	0,41%
RAG2	6	0,24%
IL7Ralpha	3	0,12%
JAK3	2	0,08%
Combined ID	74	3,02%
(no gene documented)	41	1,67%
PNP	6	0,24%
DOCK8	5	0,20%
PIK3CD (PI3K-delta)	4	0,16%
CTLA-4	4	0,16%
Cernunnos	3	0,12%
Artemis	2	0,08%
Orai1 (TMEM142A)	2	0,08%
PRKDC	2	0,08%
LIG4	2	0,08%
STIM1	1	0,04%
ITK	1	0,04%
LRBA	1	0,04%
Unclassified immunodeficiencies	76	3,06%
Unclassified IDs	76	3,06%
(no gene documented)	75	3,02%
TACI	1	0,04%
**Total**	**1,825**	**74,40%**

S6: The 12 foremost PIDs and genes within their main category

Main PID Category	Number of patients March 2012		Number of patients 4.7.2017	
Predominantly antibody disorders	858	(63%)	1390	(57%)
Other well defined PIDs	171	(13%)	283	(12%)
Phagocytic disorders	111	(8%)	199	(8%)
Predominantly T-cell deficiencies*<br>= Combined immunodeficiencies**	60	(4%)	177	(7%)
Autoimmune and immunedysregulation syndromes*<br>= Diseases of immune dysregulation**	46	(3%)	136	(6%)
Autoinflammatory syndromes*<br>= Autoinflammatory disorders	42	(3%)	84	(3%)
Unclassified immunodeficiencies	54	(4%)	76	(3%)
Defects in innate immunity	13	(1%)	45	(2%)
Complement deficiencies	13	(1%)	44	(2%)
No PID documented			19	(1%)
**Total**	**1,368**		**2,453**	

S7: Main categories of PID: Differences in grouping main categories (2012 and 2017)

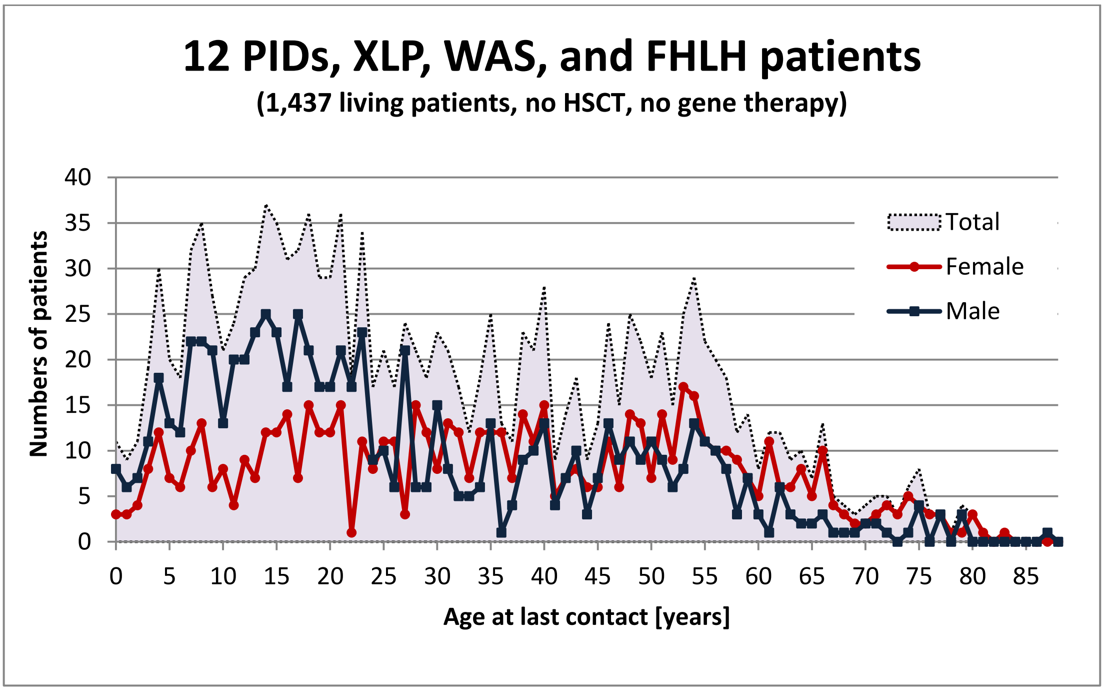

S8: Age and gender distribution of 1,437 living patients of the 12 PIDs patients groups, XLP, WAS, and FHLH patients (no HSCT or gene therapy)

(For further details, see "Age distribution by year of birth" page 69ff).

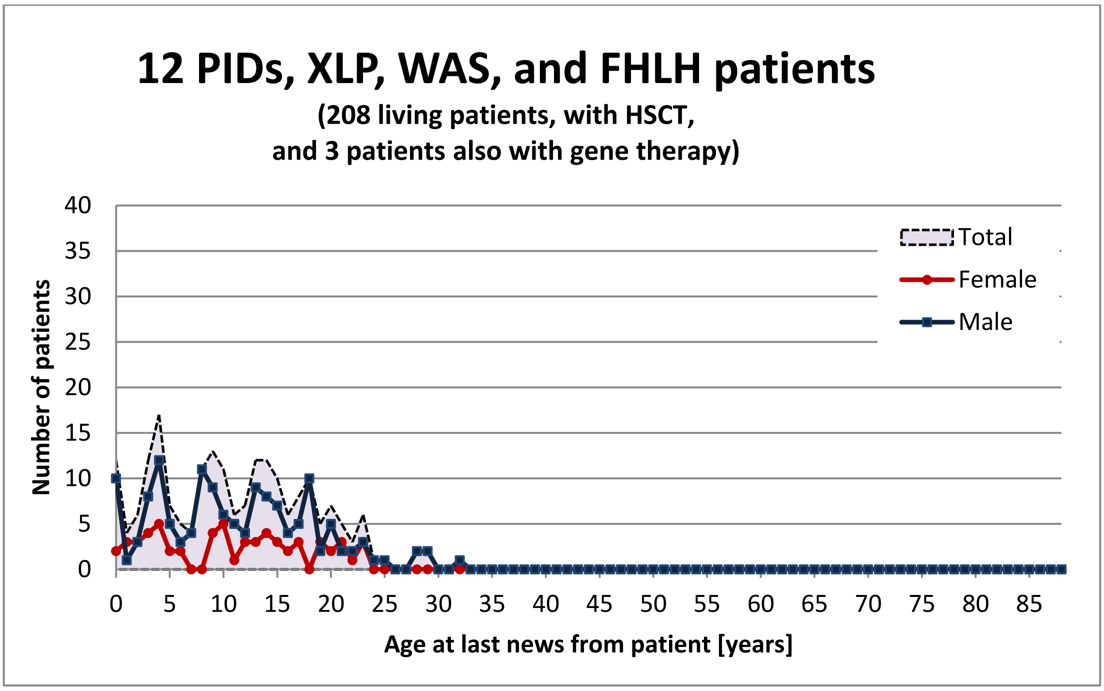

S9: Age and gender distribution of 208 living patients of the 12 PIDs patients groups, XLP, WAS, and FHLH patients (with HSCT, and 3 patients also with gene therapy)

(For further details, please see "Age distribution by year of birth" page 69ff).

12 PIDs, WAS, XLP, FHLH	Male [n]	Female [n]	Total (HSCT/ no gene therapy)		Age range [years]	Median age [years]	Living patients with and without HSCT/gene therapy
**CVID**	1	0	1	0,15%	12	12	666
**Unclassified antibody def.**			0				223
**CGD**	46	5	51	45%	0 - 29	12	114
**Agammaglobulinemia**	2		2	2%	12 - 17	15	99
**Isolated IgG subclass deficiency**			0	0%	-	-	82
**SCID**	37	27	64	83%	0 - 28	10	77
**Unclassified IDs**	1	2	3	7%	14 - 19	19	44
**Combined ID**	14	11	25	40%	0 - 24	10	63
**DGS**			0	0%	-	-	52
**Selective IgA deficiency**			0	0%	-	-	47
**A-T**	1		1	2%	9	9	57
**HIES**	2	6	8	15%	2 - 23	16	55
**WAS**	18		18	64%	0 - 25	9	28
**XLP**	7		7	32%	8 - 32	21	22
**FHLH**	7	8	15	83%	2 - 23	5	18
**Total**	**136**	**60**	**195**	**12%**	**0 - 32**		**1,647**

S10: Age range and median age at last news from patient in years of the 12 most prevalent PID diagnoses and WAS, XLP and FHLH (patients with HSCT but no gene therapy), living patients

PID	Male [n]	Female [n]	Total (gene therapy)		Age at gene therapy	Living patients with and without HSCT/gene therapy
**SCID**	2	0	2	0,3%	8 month and 8 years*, 16 month**	666
**CGD**	1	0	1	0,4%	4 years 9 months**	223
**WAS**	2	0	2	2%	2 years 5 month, 4 years**	114
**Total**	5	0	5	0,5%	N/A	1,003

* gene therapy was attempted twice
** received HSCT the same day / following gene therapy

S11: Age at gene therapy

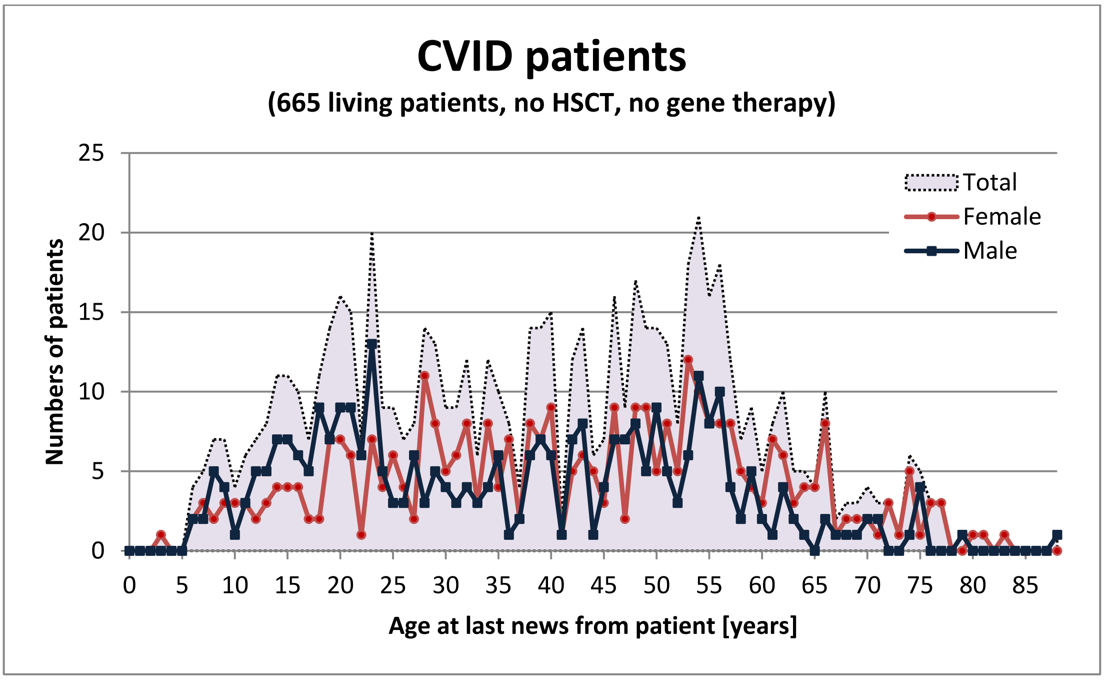

S12: Age and gender distribution of 665 living CVID patients. No patients received an HSCT or gene therapy.

(For further details, see “Age distribution by year of birth” page 69ff)

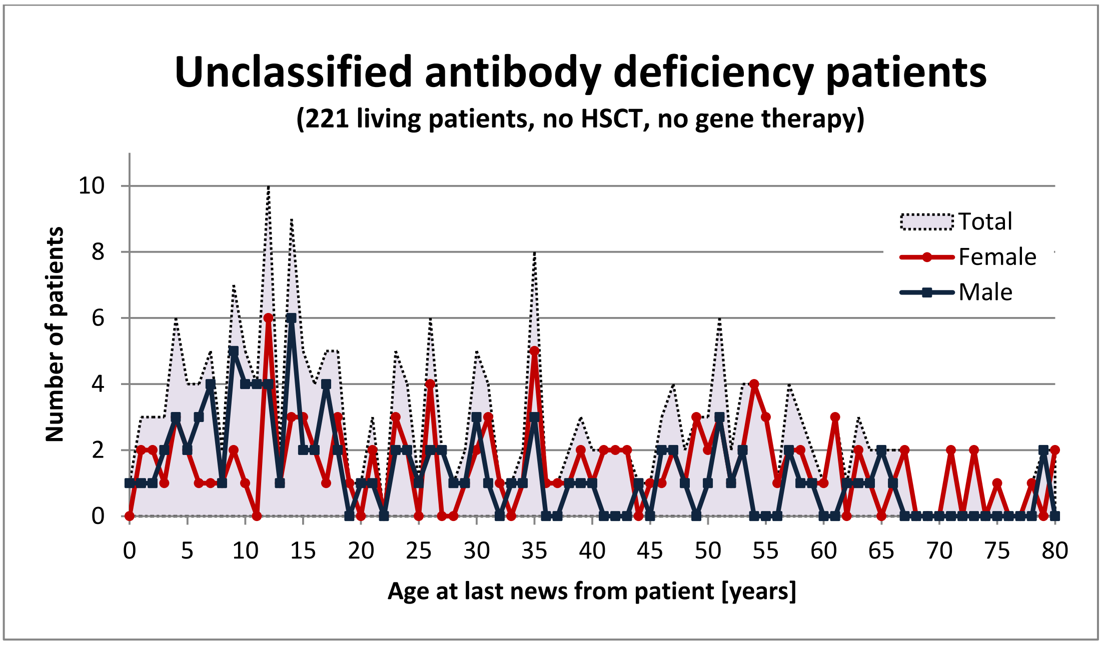

S13: Age and gender distribution of 221 living unclassified antibody deficiency patients.
No patients received an HSCT or gene therapy.

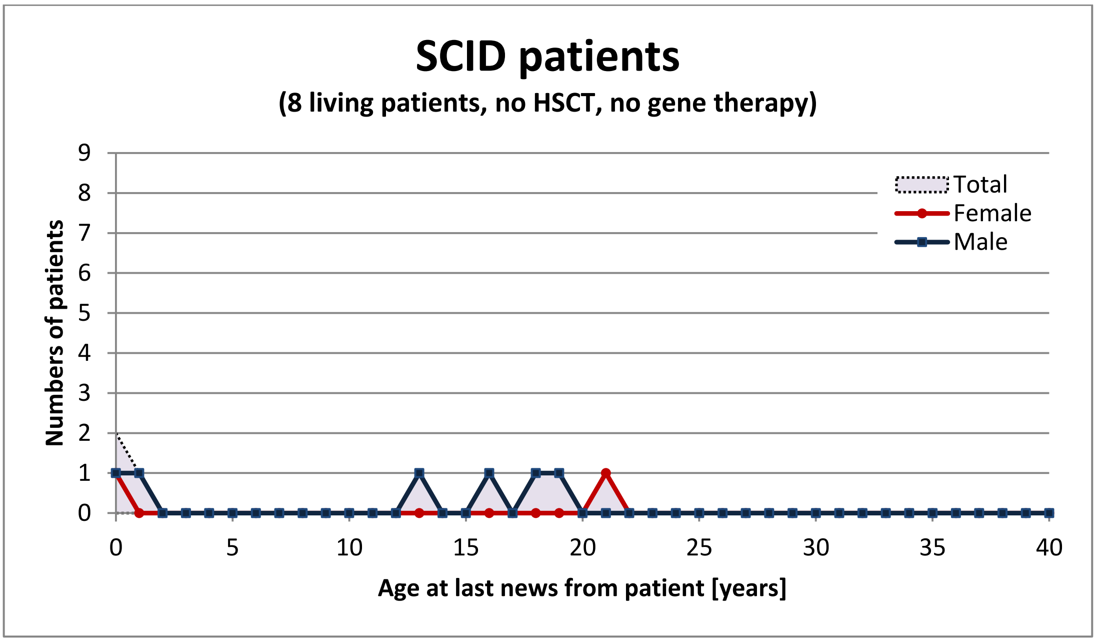

S14: Age and gender distribution of 8 living SCID patients. No patients received an HSCT or gene therapy.

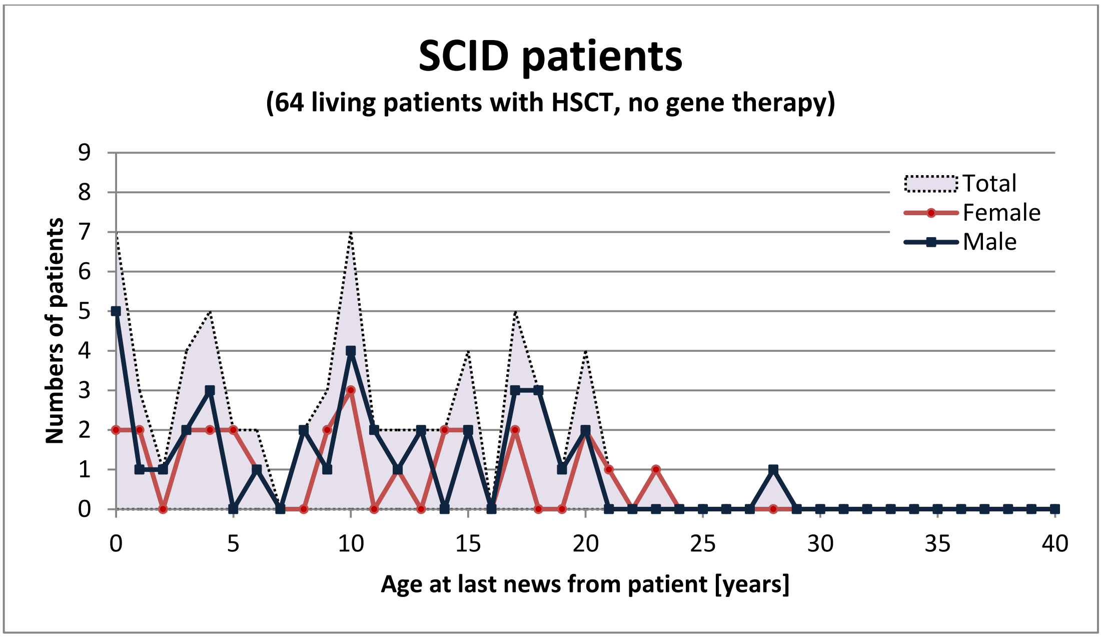

S15: Age and gender distribution of 64 living SCID patients (with HSCT, no gene therapy).

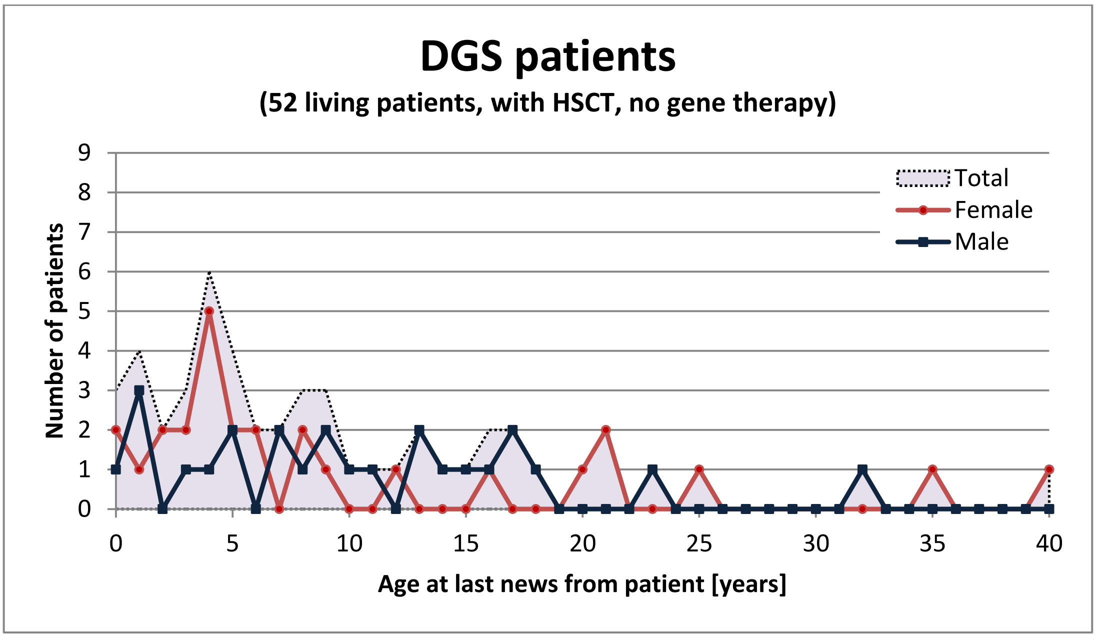

S16: Age and gender distribution of 52 living DGS patients (with HSCT, no gene therapy).

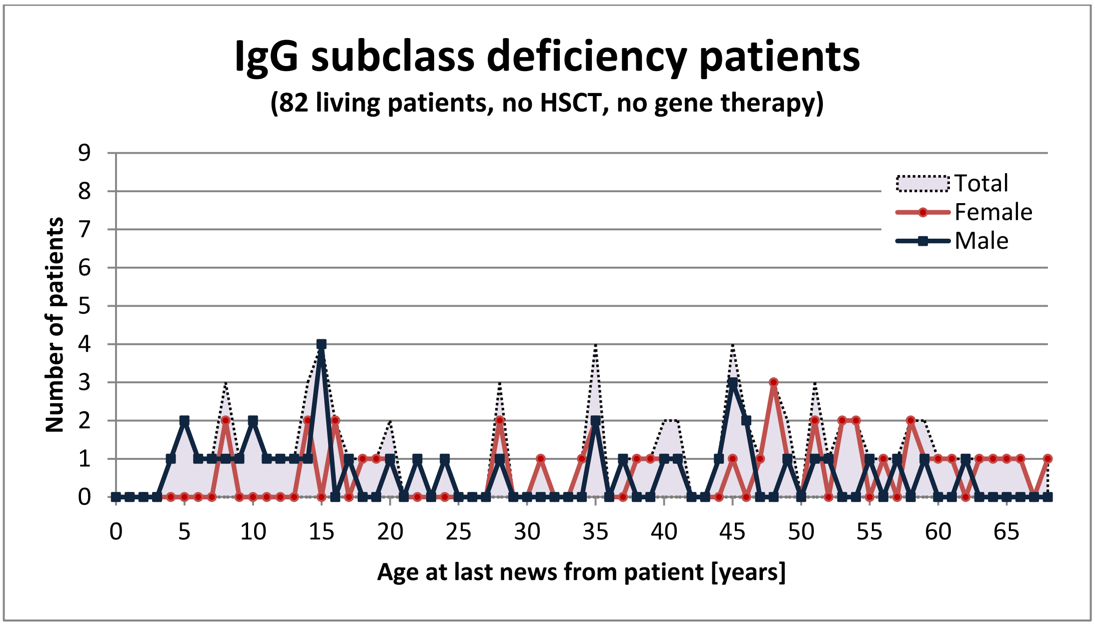

S17: Age and gender distribution of 82 living isolated IgG subclass deficiency patients. No patients received an HSCT or gene therapy.

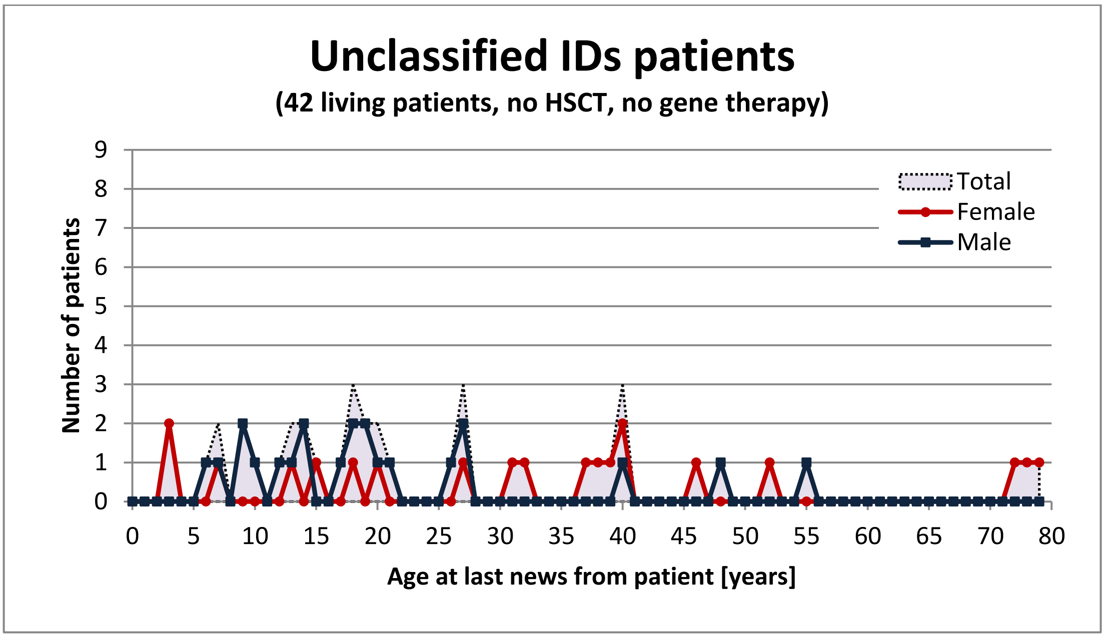

S18: Age and gender distribution of 42 living unclassified IDs. No patients received an HSCT or gene therapy.

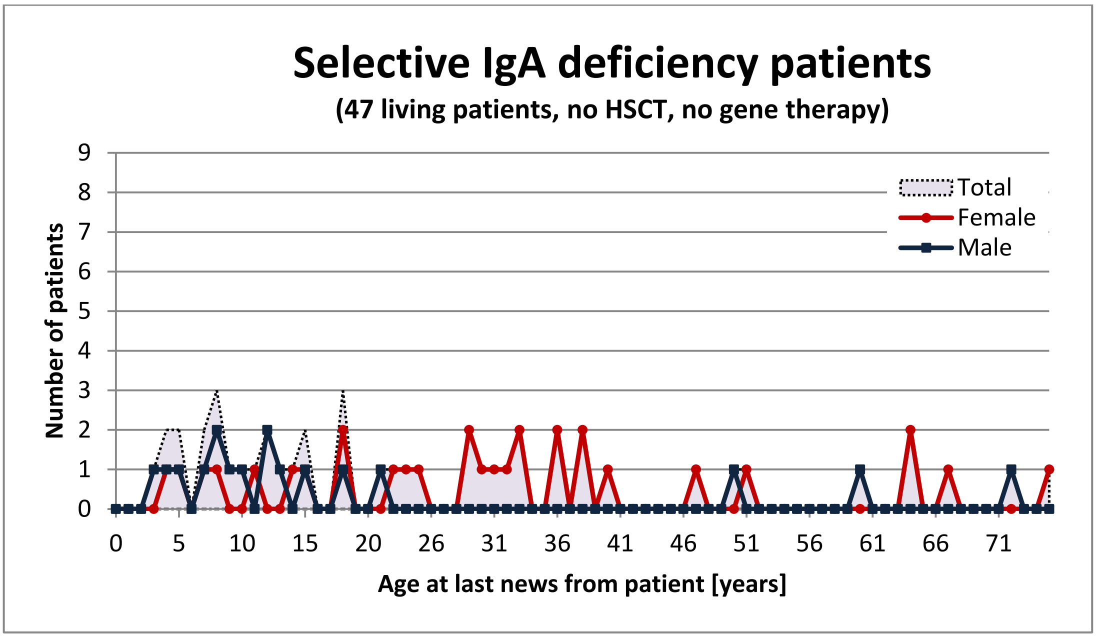

S19: Age and gender distribution of 47 living selective IgA deficiency patients. No patients received an HSCT or gene therapy.

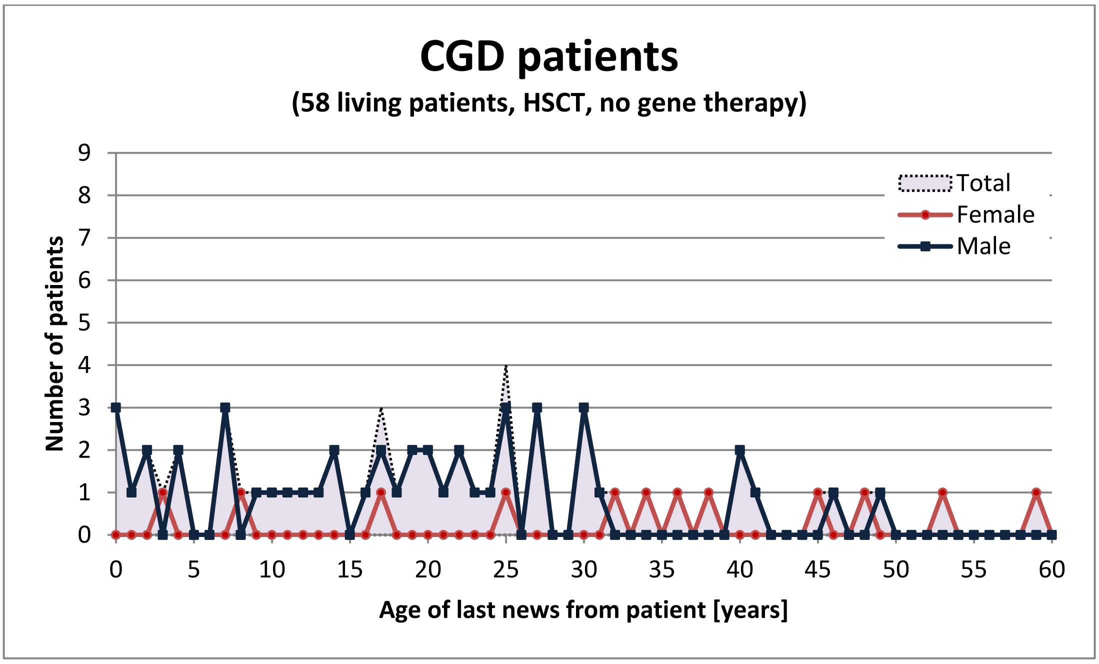

S20: Age and gender distribution of 58 living CGD patients. No patients received an HSCT or gene therapy.

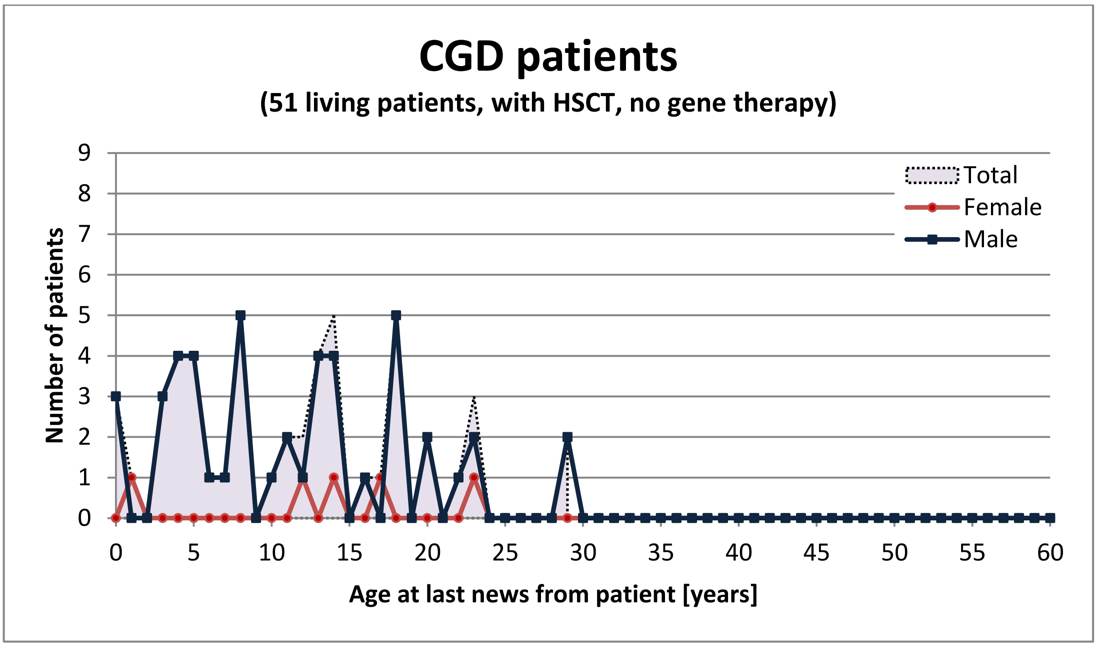

S21: Age and gender distribution of 51 living CGD patients (HSCT but no gene therapy)

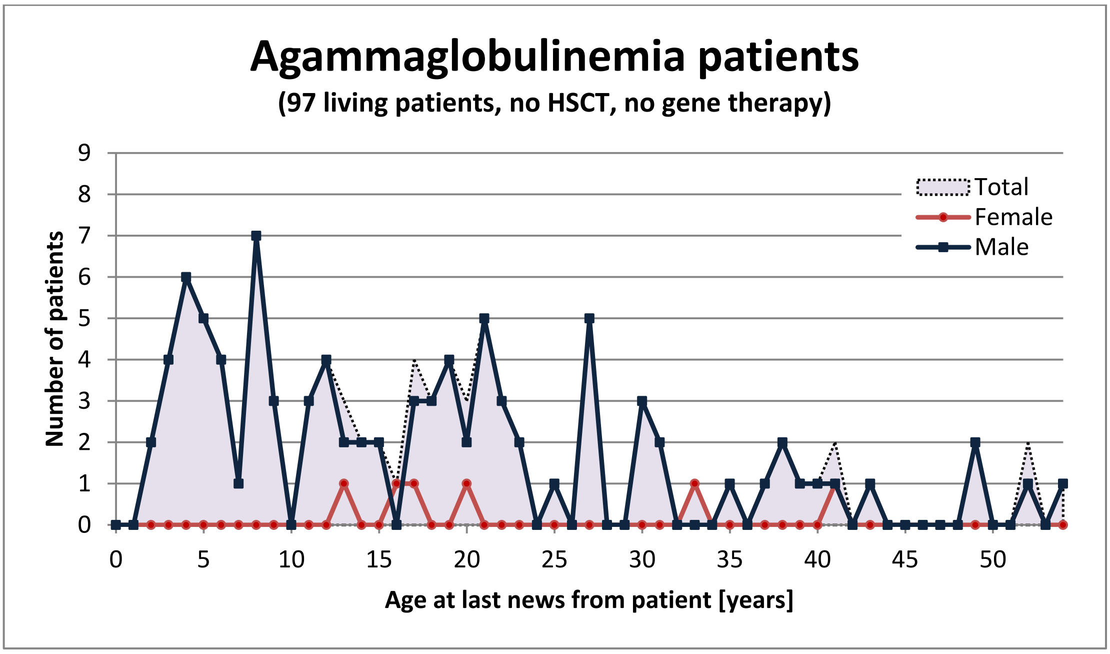

S22: Age and gender distribution of 97 living agammaglobulinemia. No patients received an HSCT or gene therapy.

Two patients with 12 and 17 years who received HSCT are not shown here.

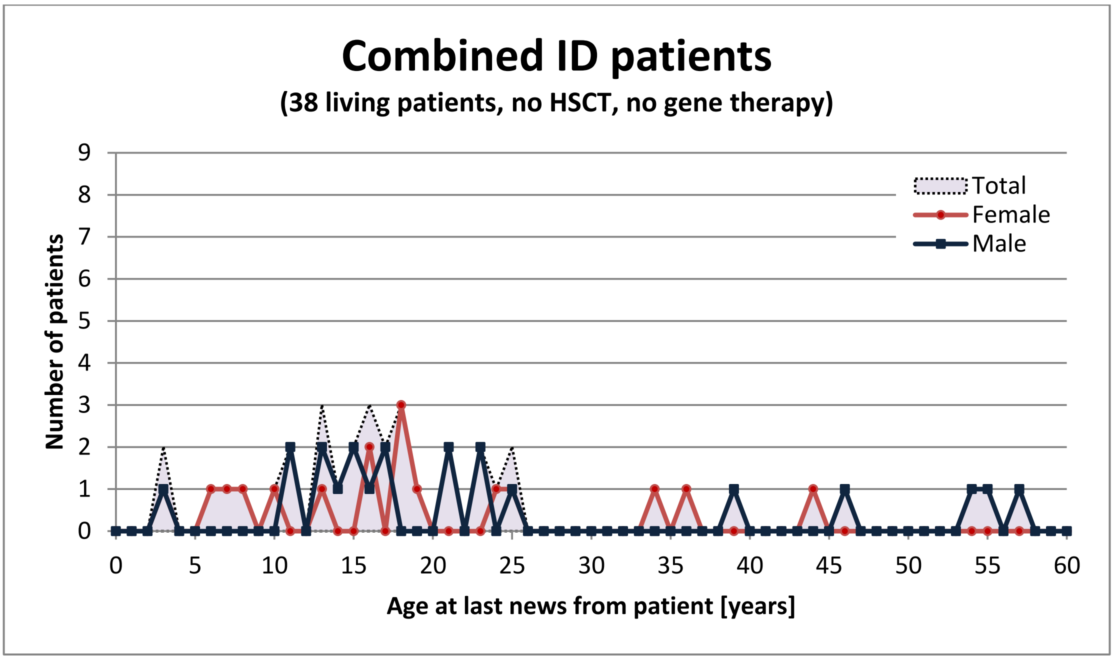

S23: Age and gender distribution of 38 living combined ID. No patients received an HSCT or gene therapy.

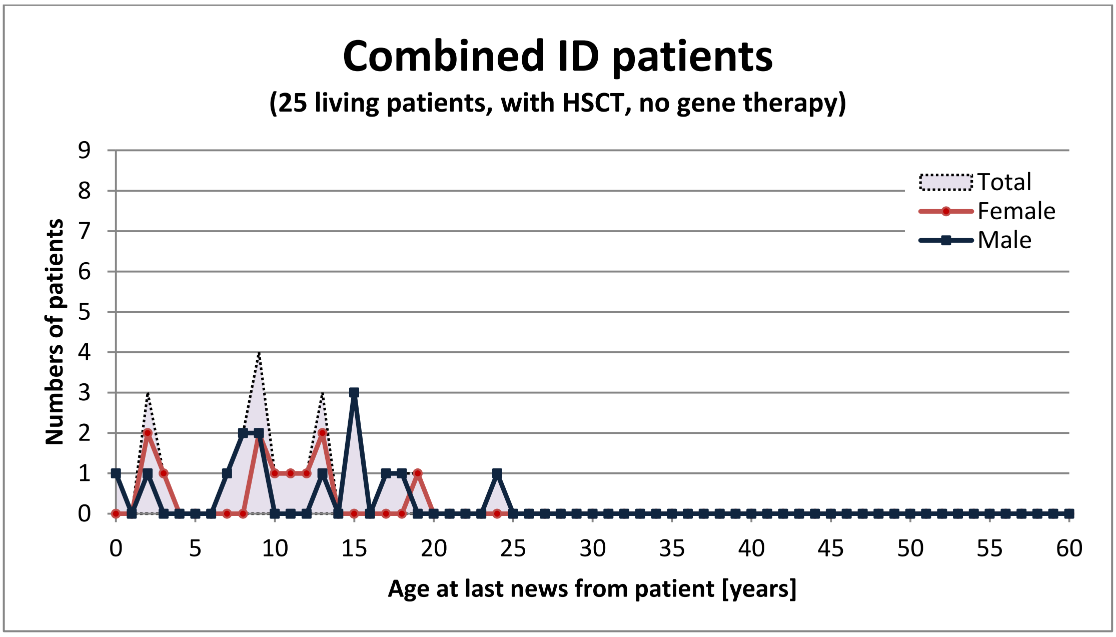

S24: Age and gender distribution of 25 living combined ID patients (HSCT but no gene therapy)

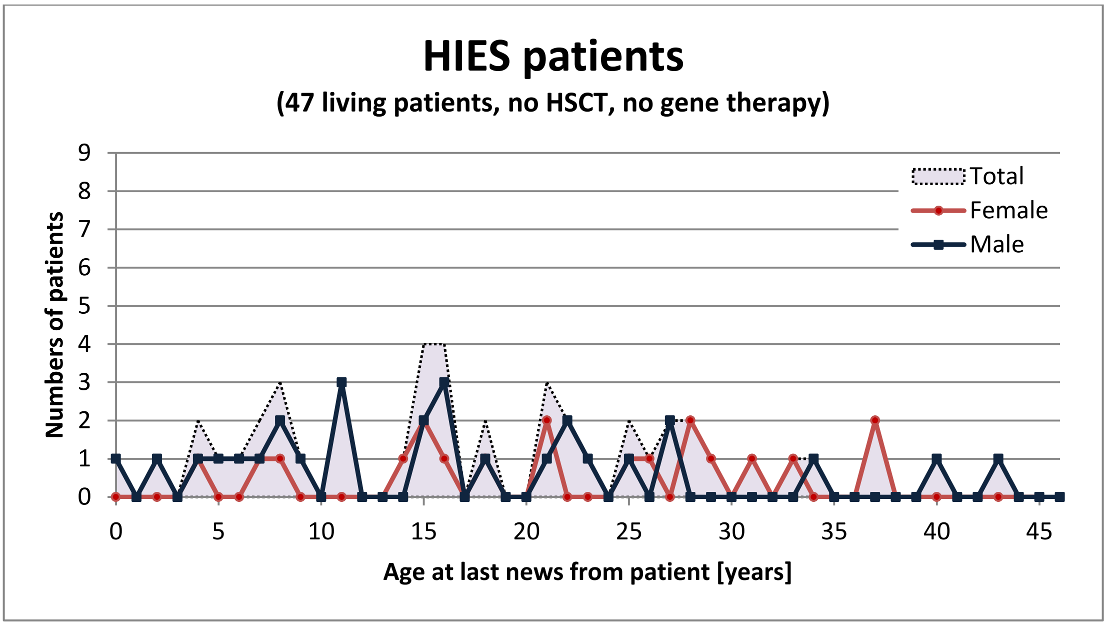

S25: Age and gender distribution of 47 living HIES patients. No patients received an HSCT or gene therapy.

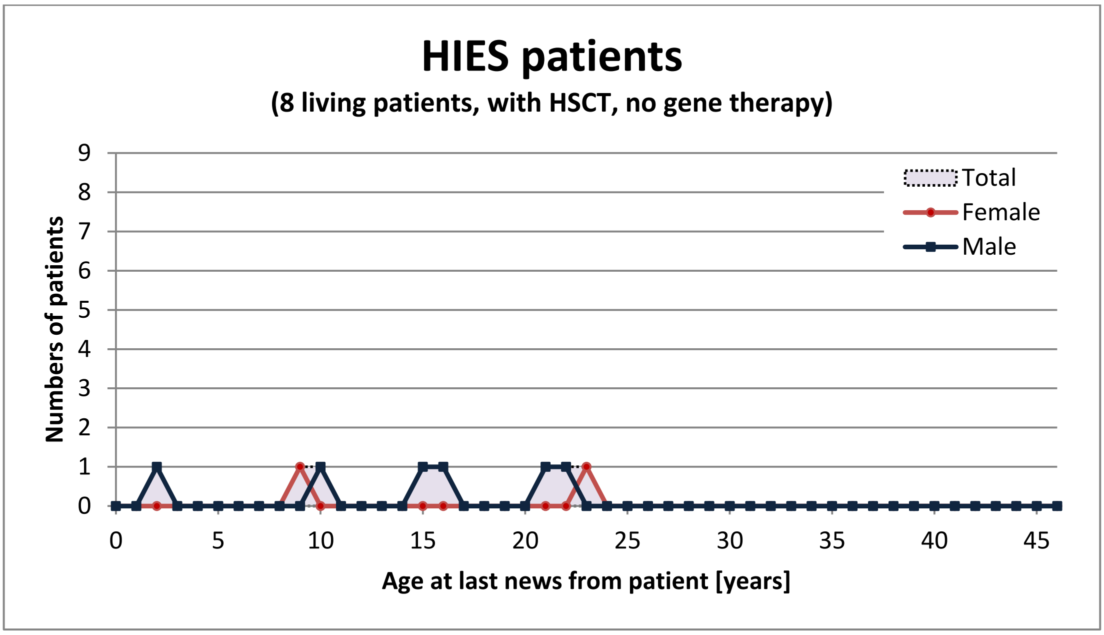

S26: Age and gender distribution of 8 living HIES patients (HSCT but no gene therapy)

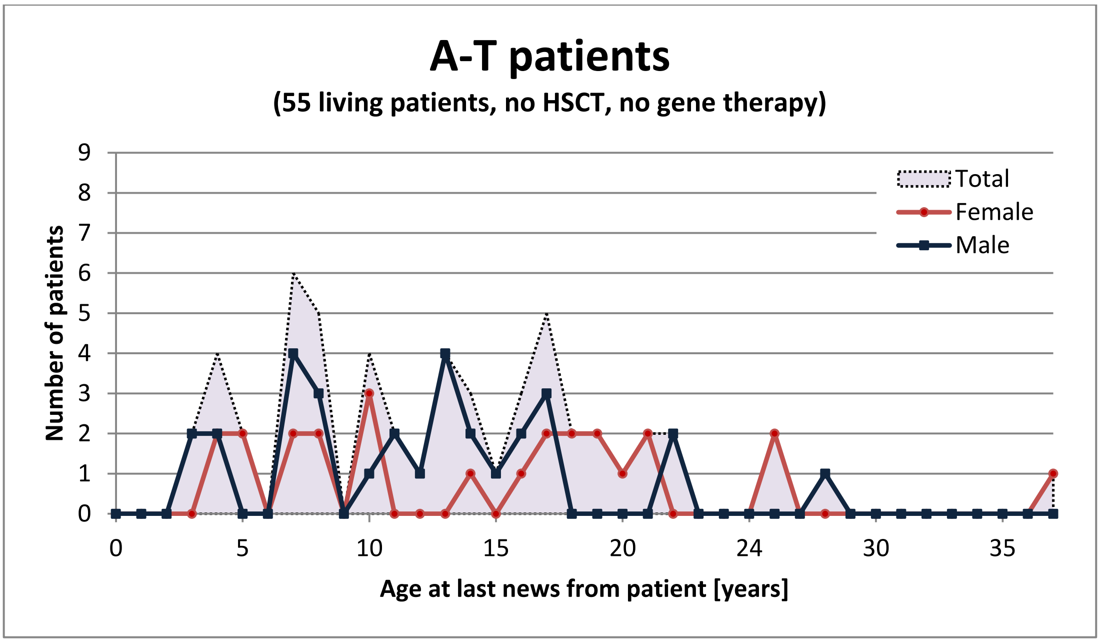

S27: Age and gender distribution of 55 living A-T patients. No patients received an HSCT or gene therapy.

Only 1 male 9 year old patient received HSCT who is not included here.

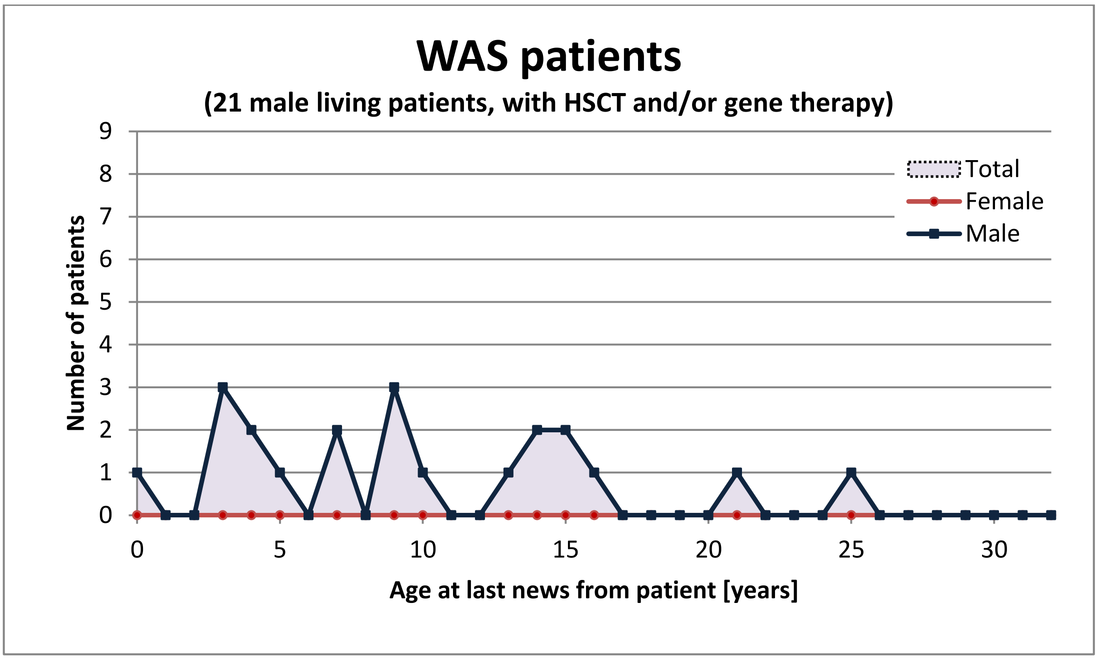

S28: Age-and gender distribution of 21 male WAS patients receiving HSCT and/or gene therapy.

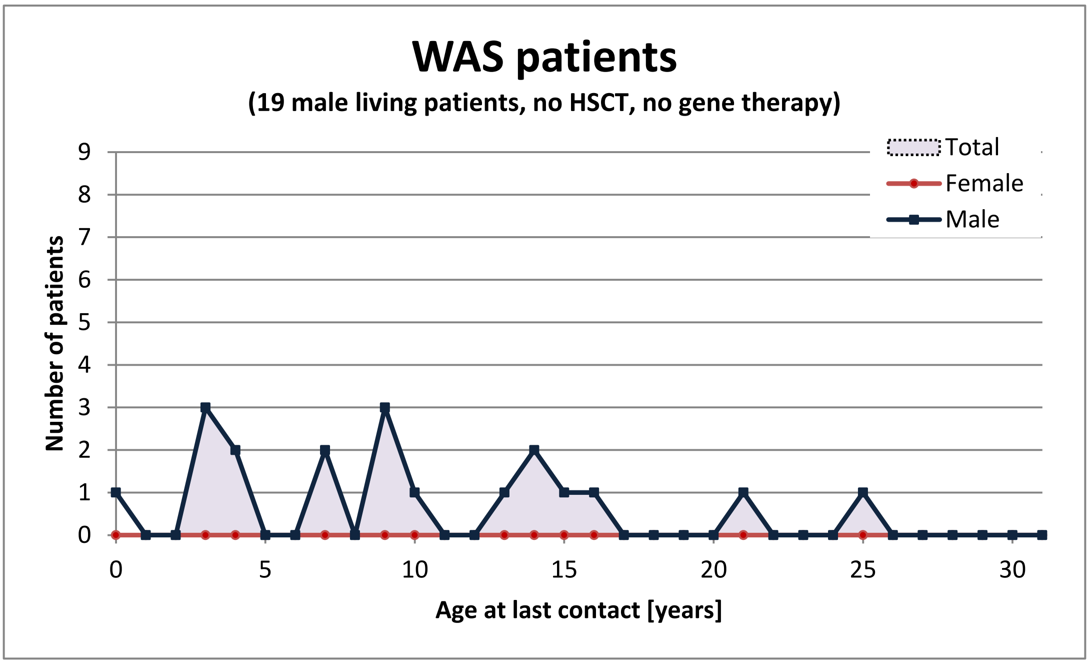

S29: Age-and gender distribution of 18 living WAS patients (HSCT but no gene therapy)

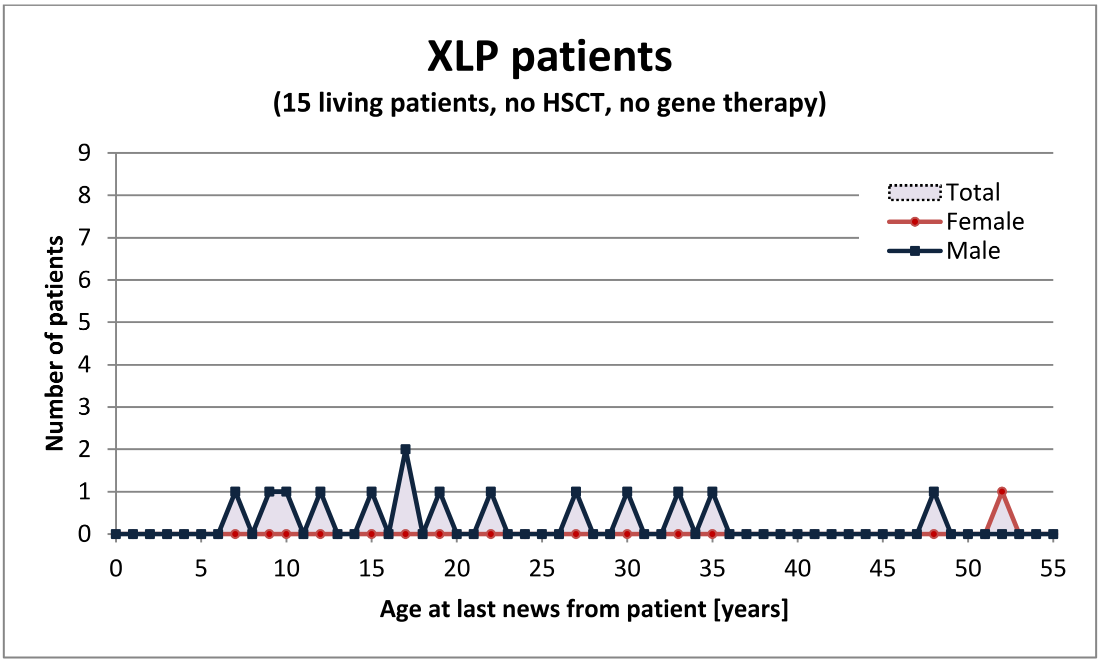

S30: Age-and gender distribution of 15 living XLP patients. No patients received an HSCT or gene therapy.

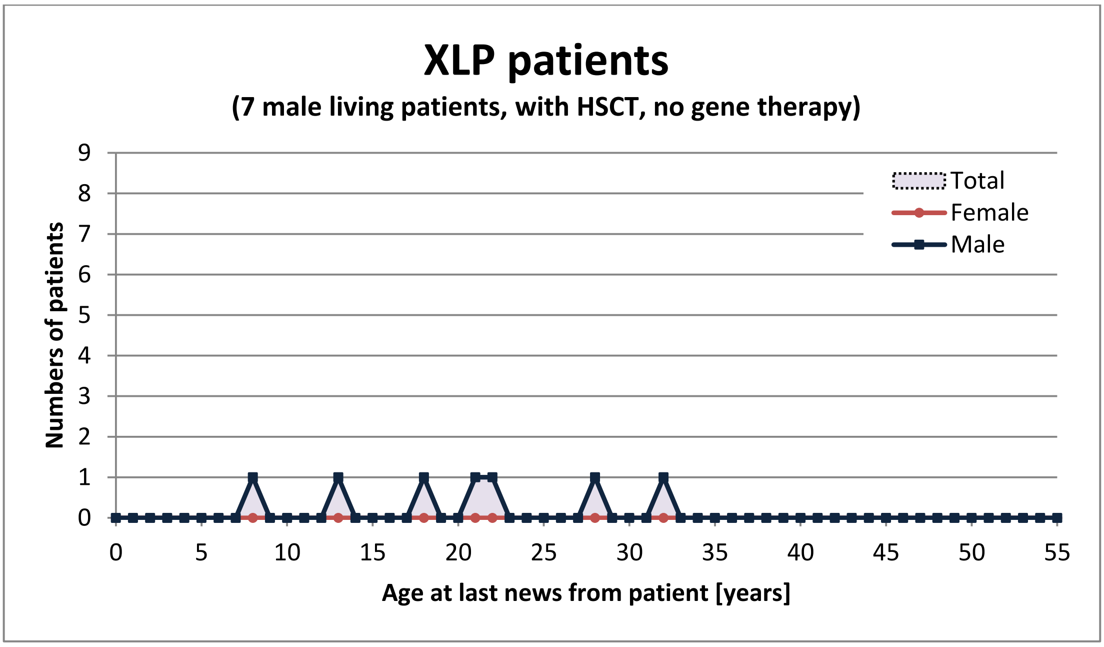

S31: Age-and gender distribution of 7 living XLP patients (HSCT but no gene therapy)

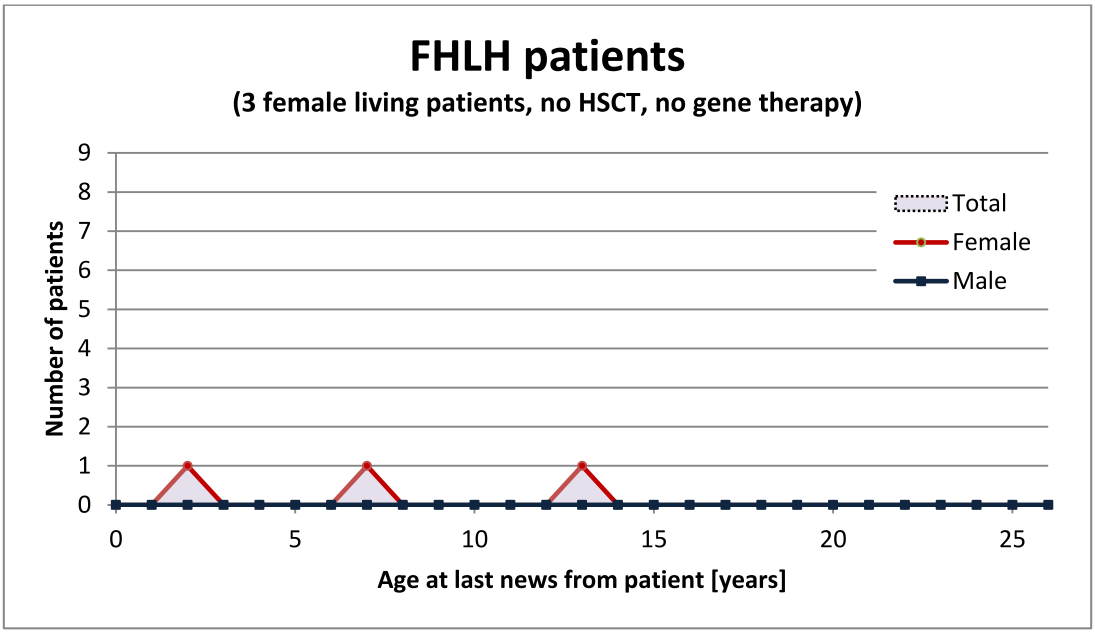

S32: Age-and gender distribution of 3 living FHLH patients. No patients received an HSCT or gene therapy.

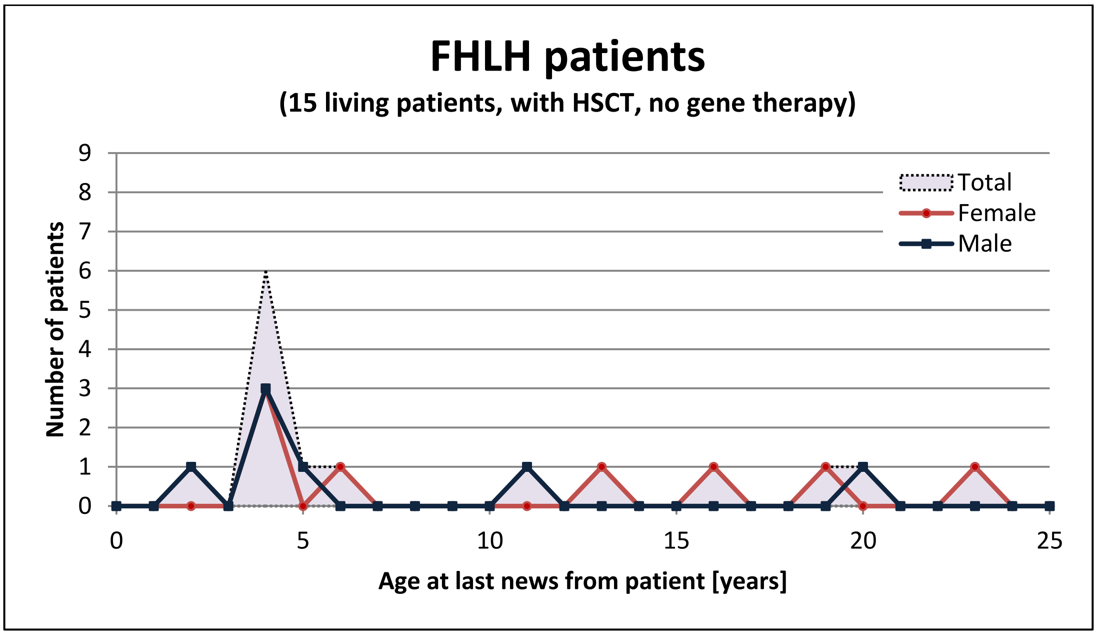

S33: Age-and gender distribution of 15 living FHLH patients (HSCT but no gene therapy)

PID	Number of patients	No gene registered	%	Pending	%	No defect found*	%	Not genetic-ally tested	%	Genetic test: unknown or no information	%	Mutation found	%
CVID	728	707	97%	9	1%	49	7%	505	69%	144	20%	21	3%
Unclassified antibody def.	267	263	99%	7	3%	16	6%	212	79%	28	10%	4	1%
Isolated IgG subclass def.	101	101	100%	0	0%	3	3%	75	74%	23	23%	0	0%
Unclassified IDs	75	74	99%	6	8%	16	21%	24	32%	28	37%	1	1%
Selective IgA deficiency	69	69	100%	0	0%	2	3%	52	75%	15	22%	0	0%
IgA with IgG subclass def.	26	26	100%	0	0%	0	0%	23	88%	3	12%	0	0%
Unclassified autoinflammatory	22	22	100%	1	5%	3	14%	17	77%	1	5%	0	0%
Transient hypogamma-globulinemia of infancy	21	21	100%	0	0%	0	0%	21	100%	0	0%	0	0%
Unclassified disorders of immune dysregulation	20	20	100%	1	5%	7	35%	11	55%	1	5%	0	0%
No PID Diagnosis documented yet	19	19	100%	0	0%	0	0%	0	0%	19	100%	0	0%
Specific IgG def (SPAD)	18	18	100%	0	0%	1	6%	6	33%	11	61%	0	0%
Syndromic PID, unclassified	16	16	100%	2	13%	7	44%	3	19%	4	25%	0	0%
Innate ID, unclassified	12	12	100%	4	33%	5	42%	1	8%	2	17%	0	0%
Unclassified phagocytic disorders	10	10	100%	2	20%	5	50%	1	10%	2	20%	0	0%
Omenn syndrome	5	3	60%	0	0%	3	60%	0	0%	0	0%	2	40%
Complement ID, unclassified	5	5	100%	0	0%	1	20%	4	80%	0	0%	0	0%
Selective IgM deficiency	5	5	100%	0	0%	0	0%	3	60%	2	40%	0	0%
Ivemark syndrome	4	4	100%	0	0%	0	0%	2	50%	2	50%	0	0%
Isolated congenital asplenia	2	2	100%	0	0%	0	0%	2	100%	0	0%	0	0%
Thymoma with immunodef.	2	2	100%	0	0%	0	0%	2	100%	0	0%	0	0%
Acquired angioedema	1	1	100%	0	0%	0	0%	1	100%	0	0%	0	0%
**Total**	**1,428**	**1,400**	**100%**	**32**	**2%**	**118**	**8%**	**965**	**68%**	**285**	**20%**	**28**	**2%**

*= Patient has been genetically tested but no defect has been found.

S34: PIDs with nearly no genetic information

12 PIDs	Number of patients	No gene regi-stered	%	Pending	%	No defect found*	%	Not geneti-cally tested	%	Genetic test: unknown or no information	%	Mut-ation found	%
**CVID**	728	707	97%	9	1%	49	7%	505	69%	144	20%	21	3%
**Unclassified antibody deficiency**	267	263	99%	7	3%	16	6%	212	79%	28	10%	4	1%
**CGD**	129	17	13%	4	3%	1	1%	5	4%	7	5%	112	87%
**Agammaglobulinemia**	112	16	14%	0	0%	9	8%	3	3%	4	4%	96	86%
**Isolated IgG subclass deficiency**	101	101	100%	0	0%	3	3%	75	74%	23	23%	0	0%
**SCID**	83	19	24%	3	4%	6	7%	2	2%	8	10%	64	76%
**Unclassified IDs**	76	75	99%	6	8%	16	21%	24	32%	29	38%	1	1%
**Combined ID**	74	41	55%	7	9%	17	23%	13	18%	4	5%	33	45%
**Selective IgA deficiency**	69	69	100%	0	0%	2	3%	52	75%	15	22%	0	0%
**A-T**	66	3	5%	0	0%	0	0%	1	2%	2	3%	63	95%
**DGS**	65	1	2%	0	0%	0	0%	0	0%	1	2%	64	98%
**HIES**	55	9	16%	1	2%	6	11%	1	2%	1	2%	46	84%
**Total**	**1,825**	**1,321**	**72%**	**37**	**2%**	**125**	**7%**	**893**	**49%**	**266**	**15%**	**504**	**28%**

*= Patient has been genetically tested but no defect has been found.

S35: 12 PIDs: genetic information

PID/Gene	Female [n]	%	Male [n]	%	Total [n]
**HIES: *STAT3***	13	36%	23	64%	**36**
**FMF: *MEFV***	9	38%	15	63%	**24**
**A-T: *ATM***	26	41%	37	59%	**63**
**HAE: C1 Inhibitor**	15	75%	5	25%	**20**

S36: Mainly male and female patients in PID with non-X-linked gene defect, with a diagnosed gene defect

Main category	Number of patients	Consanguinity		Familial case		Relative to index patient	
**Autoinflammatory disorders**	84	7	8%	26	31%	6	7%
**Combined immunodeficiencies**	178	44	25%	46	26%	9	5%
**Complement deficiencies**	44	2	5%	25	57%	9	20%
**Defects in innate immunity**	45	7	16%	18	40%	5	11%
**Diseases of immune dysregulation**	136	15	11%	56	41%	24	18%
**No PID entered**	19		0%	4	21%	1	5%
**Other well defined PIDs**	283	38	13%	61	22%	23	8%
**Phagocytic disorders**	199	26	13%	55	28%	16	8%
**Predominantly antibody disorders**	1,390	40	3%	218	16%	62	4%
**Unclassified immunodeficiencies**	75	7	9%	12	16%	5	7%
**Total**	**2,453**	**186**		**521**		**160**	

S37: Consanguinity and family case in main categories

Main category	Subcategory	Disease	Number of patients	Male	Female	Deceased	Lost to follow-up	Mutated gene identified	Consanguinity	Familial case
Predominantly antibody disorders			1,390	53%	47%	18	88	11%	3%	16%
	Agammaglobulinemias		112	93%	7%		6	86%	4%	29%
		Agammaglobulinemia	112	93%	7%		6	86%	4%	29%
	Class switch recombination defects (CSR) / HIGM syndromes		38	76%	24%		1	71%	24%	24%
		CSR defects and Hyper IgM (HIGM) syndromes	38	76%	24%		1	71%	24%	24%
	Hypogammaglobulinemias		970	48%	52%	13	56	2%	2%	14%
		Common variable immuno-deficiency disorders (CVID)	728	48%	52%	12	30	3%	2%	14%
		Deficiency of specific IgG (Specific antibody deficiency - SPAD)	18	72%	28%		4	0%	11%	39%
		IgA with IgG subclass deficiency	26	27%	73%		1	0%	8%	12%
		Isolated IgG subclass deficiency	101	50%	50%	1	8	0%	2%	15%
		Selective IgA deficiency	69	42%	59%		11	0%	3%	10%
		Selective IgM deficiency	5	80%	20%			0%	0%	0%
		Thymoma with immunodeficiency	2	100%	0%			0%	0%	0%
		Transient hypogamma-globulinemia of infancy	21	76%	23%		2	0%	5%	14%
	Unclassified antibody deficiency		270	50%	51%	5	25	3%	1%	14%
		Steinert myotonica dystrophia	3	33%	67%	1		100%	0%	0%
		Unclassified antibody deficiency	267	50%	51%	4	25	1%	1%	15%
Other well defined PIDs			283	58%	41%	8	12	85%	13%	22%
	Asplenia syndrome		6	17%	80%		1	0%	0%	17%
		Asplenia syndrome (Ivemark syndrome)	4	25%	67%		1	0%	0%	0%
		Isolated congenital asplenia	2	0%	100%			0%	0%	50%

Main category	Subcategory	Disease	Number of patients	Male	Female	Deceased	Lost to follow-up	Mutated gene identified	Consanguinity	Familial case
	Cartilage hair hypoplasia		4	50%	50%	1		100%	0%	50%
		Cartilage hair hypoplasia	4	50%	50%	1		100%	0%	50%
	DiGeorge syndrome		65	48%	52%		4	98%	2%	2%
		DiGeorge syndrome (DGS)	65	48%	52%		4	98%	2%	2%
	DNA-breakage disorder		80	59%	41%	7	3	95%	26%	23%
		Ataxia telangiectasia (A-T)	66	58%	42%	6	3	95%	30%	23%
		Bloom syndrome	1	0%	100%			100%	0%	0%
		Immunodeficiency centromeric instability facial anomalies syndrome (ICF)	2	50%	50%			50%	50%	0%
		Nijmegen breakage syndrome (NBS1)	10	70%	30%	1		100%	0%	30%
		Post-Meiotic Segregation 2 (PMS2) deficiency	1	100%	0%			100%	0%	0%
	Dyskeratosis congenita		7	57%	43%		1	86%	29%	43%
		Dyskeratosis congenita	7	57%	43%		1	86%	29%	43%
	Fc receptor deficiencies		1	100%	0%			100%	0%	0%
		Fc receptor deficiencies	1	100%	0%			100%	0%	0%
	Hyper IgE syndromes		55	55%	45%			84%	15%	38%
		Hyper IgE syndrome (HIES)	55	55%	45%			84%	15%	38%
	MonoMAC		3	33%	67%			100%	0%	33%
		Monocytopenia and mycobacterial infection (MonoMAC)	3	33%	67%			100%	0%	33%
	Netherton syndrome		5	20%	80%			100%	40%	40%
		Netherton syndrome	5	20%	80%			100%	40%	40%
	Other syndromic PID		3	33%	67%			100%	0%	33%
		Activated PI3K-delta syndrome (APDS)	3	33%	67%			100%	0%	33%
	Schimke disease		2	50%	50%			100%	50%	0%
		Schimke disease	2	50%	50%			100%	50%	0%

Main category	Subcategory	Disease	Number of patients	Male	Female	Deceased	Lost to follow-up	Mutated gene identified	Consanguinity	Familial case
	Unclassified syndromic immunodeficiencies		16	56%	44%		1	0%	19%	19%
		Unclassified syndromic immunodeficiencies	16	56%	44%		1	0%	19%	19%
	Wiskott-Aldrich syndrome (WAS)		36	100%	0%		2	86%	0%	22%
		Wiskott-Aldrich syndrome (WAS)	29	100%	0%		1	83%	0%	14%
		X-linked thrombocytopenia with mutations in WASP	7	100%	0%		1	100%	0%	57%
Phagocytic disorders			199	71%	28%	7	5	79%	13%	28%
	Chronic granulomatous disease (CGD)		129	84%	16%	7	5	87%	12%	31%
		Chronic granulomatous disease (CGD)	129	84%	16%	7	5	87%	12%	31%
	Congenital neutropenia		32	41%	58%			63%	6%	22%
		Congenital neutropenia	32	42%	58%			63%	6%	22%
	Cyclic neutropenia		4	50%	50%			100%	0%	25%
		Cyclic neutropenia	4	50%	50%			100%	0%	25%
	Defects with susceptibility to mycobacterial infection (MSMD)		9	44%	56%			78%	33%	22%
		Defects with susceptibility to mycobacterial infection (MSMD)	9	44%	56%			78%	33%	22%
	Glycogen storage disease type 1b (GS1b)		1	0%	100%			100%	0%	0%
		Glycogen storage disease type 1b (GS1b)	1	0%	100%			100%	0%	0%
	Leukocyte adhesion deficiency (LAD)		2	50%	50%			100%	50%	0%
		Leukocyte adhesion deficiency (LAD)	2	50%	50%			100%	50%	0%
	Neutrophil glucose-6-phosphate dehydrogenase		1	100%	0%			100%	100%	0%
		Glucose-6-phosphate dehydrogenase deficiency (G6PD)	1	100%	0%			100%	100%	0%

Main category	Subcategory	Disease	Number of patients	Male	Female	Deceased	Lost to follow-up	Mutated gene identified	Consanguinity	Familial case
	Shwachman-Diamond-syndrome		11	64%	36%			100%	9%	0%
		Shwachman-Diamond-syndrome	11	64%	36%			100%	9%	0%
	Unclassified phagocytic disorders		10	60%	40%			0%	30%	50%
		Unclassified phagocytic disorders	10	60%	40%			0%	30%	50%
Combined immunodeficiencies			177	56%	44%	11	8	63%	25%	26%
	Atypical severe combined immunodeficiency (Atypical SCID)		7	57%	43%	1		100%	43%	0%
		Atypical severe combined immunodeficiency (Atypical SCID)	7	57%	43%	1		100%	43%	0%
	CD4-deficiency		2	0%	100%			50%	0%	0%
		Selective CD4 cell deficiency	2	0%	100%			50%	0%	0%
	Combined immunodeficiency (CID)		74	55%	45%	5	5	45%	23%	26%
		Combined immunodeficiency (CID)	74	55%	45%	5	5	45%	23%	26%
	HLA class II deficiency		5	40%	60%	1		80%	0%	0%
		HLA class II deficiency	5	40%	60%	1		80%	0%	0%
	Omenn syndrome		5	40%	60%	1		40%	0%	40%
		Omenn syndrome	5	40%	60%	1		40%	0%	40%
	Severe combined immunodeficiency (SCID)		84	61%	39%	3	3	77%	29%	30%
		Reticular Dysgenesis - AK2 (SCID)	1	0%	100%			100%	0%	0%
		Severe combined immunodeficiency (SCID)	83	61%	39%	3	3	77%	29%	30%

Main category	Subcategory	Disease	Number of patients	Male	Female	Deceased	Lost to follow-up	Mutated gene identified	Consanguinity	Familial case
Diseases of immune dysregulation			136	66%	34%	4	8	77%	11%	41%
	Autoimmune lymphoproliferative syndrome (ALPS)		39	59%	41%		2	90%	5%	49%
		Autoimmune lymphoproliferative syndrome (ALPS)	38	61%	39%		2	89%	5%	50%
		Ras associated lympho-proliferative disease (RALD)	1	0%	100%			100%	0%	0%
	Autoimmune polyendocrinopathy candidiasis ectodermal dystrophy (APECED)		4	0%	100%		1	100%	0%	0%
		APECED / APS1 with CMC - Autoimmune polyendocrinopathy candidiasis ectodermal dystrophy (APECED)	4	0%	100%		1	100%	0%	0%
	Early-onset inflammatory bowel disease		6	50%	50%			83%	83%	50%
		Early-onset inflammatory bowel disease	5	40%	60%			80%	100%	60%
		Inflammatory Bowel Disease-like	1	100%	0%			100%	0%	0%
	Early-onset multi-organ autoimmune disease		2	0%	100%			100%	0%	0%
		Early-onset multi-organ autoimmune disease	2	0%	100%			100%	0%	0%
	Hemophagocytic lymphohistiocytosis (HLH)		58	76%	24%	3	5	90%	12%	50%
		CD27 deficiency	1	0%	100%			100%	0%	0%
		Chediak Higashi syndrome	4	50%	50%			75%	0%	0%
		Familial hemophagocytic lymphohistiocytosis syndromes (FHLH)	20	60%	40%	1	1	80%	25%	50%
		Griscelli syndrome, type 2	3	67%	33%			67%	33%	67%
		Hermansky-Pudlak syndrome	1	0%	100%			100%	100%	100%

Main category	Subcategory	Disease	Number of patients	Male	Female	Deceased	Lost to follow-up	Mutated gene identified	Consanguinity	Familial case
		X-linked lymphoproliferative syndrome (XLP)	29	97%	3%	2	4	100%	0%	55%
	IPEX		6	100%	0%			100%	17%	0%
		FOXP3 deficiency (IPEX)	5	100%	0%			100%	0%	0%
		Interleukin 2 receptor alpha deficiency (CD25) (IPEX phenotype)	1	100%	0%			100%	100%	0%
	Type 1 interferonopathies		1	0%	100%			100%	0%	0%
		Type 1 interferonopathies	1	0%	100%			100%	0%	0%
	Unclassified disorders of immune dysregulation		20	70%	30%	1		0%	0%	25%
		Unclassified disorders of immune dysregulation	20	70%	30%	1		0%	0%	25%
Autoinflammatory disorders			84	64%	36%	1	14	74%	8%	31%
	CINCA syndrome		6	67%	33%		1	100%	17%	33%
		CINCA syndrome	6	67%	33%		1	100%	17%	33%
	Familial cold autoinflammatory syndrome		2	100%	0%			100%	0%	0%
		Familial cold autoinflammatory syndrome	2	100%	0%			100%	0%	0%
	Familial Mediterranean fever (FMF)		24	63%	38%		4	100%	21%	42%
		Familial Mediterranean fever defect (FMF)	24	63%	38%		4	100%	21%	42%
	Familial periodic fever		23	52%	48%		3	100%	4%	43%
		Hyper IgD syndrome (MVK)	5	40%	60%		2	100%	20%	20%
		TNF-receptor associated periodic fever syndrome (TRAPS)	18	56%	44%		1	100%	0%	50%
	Muckle-Wells syndrome		7	57%	43%			100%	0%	29%
		Muckle-Wells syndrome	7	57%	43%			100%	0%	29%

Main category	Subcategory	Disease	Number of patients	Male	Female	Deceased	Lost to follow-up	Mutated gene identified	Consanguinity	Familial case
	Unclassified autoinflammatory diseases		22	77%	23%	1	6	0%	0%	9%
		Unclassified autoinflammatory diseases	22	77%	23%	1	6	0%	0%	9%
Unclassified immunodeficiencies			76	49%	51%		6	1%	9%	16%
	Unclassified immunodeficiencies		76	49%	51%		6	1%	10%	16%
		Unclassified immunodeficiencies	76	49%	51%		6	1%	10%	16%
Defects in innate immunity			45	56%	44%		4	64%	16%	40%
	Chronic mucocutaneous candidiasis (CMC)		19	58%	42%		3	79%	11%	42%
		Chronic mucocutaneous candidiasis (CMC)	19	58%	42%		3	79%	11%	42%
	Defects of TLR/NFkappa-B signalling		9	78%	22%			100%	33%	33%
		Defects of TLR/NFkappa-B signalling	9	78%	22%			100%	33%	33%
	Unclassified defects in innate immunity		12	42%	58%		1	0%	17%	33%
		Unclassified defects in innate immunity	12	42%	58%		1	0%	17%	33%
	Warts hypogammaglobulinemia infections and myelokathexis (WHIM)		5	40%	60%			100%	0%	60%
		Warts hypogammaglobulinemia infections and myelokathexis (WHIM)	5	40%	60%			100%	0%	60%
Complement deficiencies			44	45%	56%	2	4	77%	5%	57%
	Complement deficiency		38	45%	57%	2	4	87%	5%	63%
		Acquired angioedema	1	100%	0%			0%	0%	100%
		Complement component 1 deficiency	1	0%	100%	1		100%	100%	0%
		Complement component 2 deficiency	6	50%	50%		1	100%	0%	0%

Main category	Subcategory	Disease	Number of patients	Male	Female	Deceased	Lost to follow-up	Mutated gene identified	Consanguinity	Familial case
		Complement component 7 deficiency	1	100%	0%			100%	100%	0%
		Complement component 8 deficiency	3	67%	33%		3	100%	0%	100%
		Factor I deficiency	1	100%	0%			100%	0%	0%
		Hereditary Angioedema (HAE, C1inh)	23	30%	73%			87%	0%	78%
		Properdin P factor complement deficiency (PFC)	2	100%	0%	1		50%	0%	100%
	Mannose-binding lectin (MBL)		1	0%	100%			100%	0%	0%
		Mannose-binding lectin deficiency (MBL)	1	0%	100%			100%	0%	0%
	Unclassified complement deficiencies		5	60%	40%			0%	0%	20%
		Unclassified complement deficiencies	5	60%	40%			0%	0%	20%
No PID entered			19	68%	32%			0%		21%
		no PID entered	19	68%	32%			0%	0%	21%
**Total**			**2,453**			**51**	**149**			

S38: Gender, deceased, lost, gene identified, consanguinity, and family case by PIDs

Distribution of patients on main categories, subcategories, diseases, number of patients, share of gender distribution, number of patients deceased or lost to follow-up, and share of patients with known genetic cause, consanguinity of parents and PID case with family members of the patient. Missing values: unknown or no information given.

PID / presenting symptoms	PID patients with information	Infec-tions	%	Immune dysregu-lation	%	Syn-dromal	%	Mali-gnancy	%	Other sym-ptoms	%	No first sym-ptoms	%	Diagnosis by lab abnor-malities	%
CVID	626	577	92%	144	23%	11	2%	8	1%	31	5%	8	1%	8	1%
Unclassified antibody deficiency	238	208	87%	37	16%	17	7%	1	0,42%	20	8%	10	4%	8	3%
CGD	113	87	77%	36	32%		0%		0%	7	6%	6	5%	1	1%
Agammaglobulinemia	99	91	92%	6	6%	1	1%		0%	3	3%	5	5%	4	4%
Isolated IgG subclass deficiency	89	83	93%	9	10%	3	3%	1	1%	7	8%	2	2%	2	2%
SCID	77	59	77%	16	21%	3	4%		0%	8	10%	10	13%	2	3%
Combined ID	71	45	63%	37	52%	5	7%		0%	10	14%	3	4%	2	3%
A-T	59	15	25%	4	7%	20	34%		0%	39	66%	1	2%	1	2%
DGS	56	16	29%	4	7%	47	84%		0%	6	11%	3	5%	1	2%
Selective IgA deficiency	56	43	77%	12	21%	1	2%		0%	5	9%	3	5%	3	5%
HIES	53	42	79%	15	28%	13	25%		0%	6	11%		0%		
Unclassified IDs	50	38	76%	16	32%	3	6%	2	4%	5	10%	1	2%	1	2%
ALPS	34	6	18%	30	88%		0%		0%	3	9%	2	6%	1	3%
CSR/HIGM (Hyper-IgM)	33	32	97%	9	27%	1	3%		0%	1	3%		0%		
Congenital neutropenia	29	22	76%	5	17%	3	10%		0%	6	21%	3	10%	3	10%
XLP	27	13	48%	14	52%		0%	3	11%	3	11%	3	11%		
IgA with IgG subclass deficiency	26	25	96%	6	23%	2	8%		0%	3	12%		0%		
FMF	24	4	17%	16	67%		0%		0%	7	29%	1	4%		
WAS	24	10	42%	10	42%	4	17%		0%	9	38%	1	4%		
HAE (C1Inh)	21		0%		0%		0%		0%	16	76%	5	24%	4	19%
Transient hypogamma-globulinemia of infancy	21	14	67%	1	5%		0%		0%	1	5%	5	24%	5	24%
FHLH	20	6	30%	12	60%	2	10%		0%	2	10%	2	10%		
Unclassified disorders of immune dysregulation	20	7	35%	16	80%	1	5%		0%		0%	3	15%	2	10%
Unclassified autoinflammatory	20	1	5%	12	60%		0%		0%	8	40%		0%		

PID / presenting symptoms	PID patients with information	Infec-tions	%	Immune dysregu-lation	%	Syn-dromal	%	Mali-gnancy	%	Other sym-ptoms	%	No first sym-ptoms	%	Diagnosis by lab abnor-malities	%
TRAPS	17	2	12%	7	41%		0%		0%	6	35%	3	18%	2	12%
CMC	16	15	94%	1	6%	2	13%		0%	2	13%		0%		
Syndromic PID, unclassified	15	11	73%	4	27%	10	67%		0%	2	13%		0%		
Innate ID, unclassified	12	11	92%	2	17%	3	25%		0%	1	8%		0%		
Shwachman-Diamond-syndrome	11	8	73%	6	55%	6	55%		0%	2	18%		0%		
Specific IgG deficiency (SPAD)	11	11	100%	1	9%		0%		0%	3	27%		0%		
NBS1	9	5	56%		0%	8	89%		0%	1	11%		0%		
Unclassified phagocytic disorders	9	4	44%	3	33%	2	22%		0%	1	11%	1	11%	1	11%
TLR/NFkappa-B	8	6	75%	1	13%	1	13%		0%		0%	1	13%		
Atypical SCID	7	5	71%	1	14%	1	14%		0%	1	14%		0%		
MSMD	7	6	86%	3	43%		0%		0%	1	14%	1	14%		
Muckle-Wells syndrome	7	1	14%	5	71%	1	14%		0%	1	14%		0%		
No PID entered	7	5	71%	2	29%		0%		0%		0%		0%		
XLT (WASP)	7	1	14%	1	14%	1	14%		0%	5	71%	1	14%	1	14%
CINCA syndrome	6		0%	2	33%	2	33%		0%	4	67%		0%		
Dyskeratosis congenita	6	3	50%	2	33%	2	33%	1	17%		0%	1	17%		
C2 deficiency	5	4	80%	1	20%		0%		0%		0%		0%		
Complement ID, unclassified	5	5	100%	1	20%		0%		0%		0%		0%		
Early onset of IBD	5	2	40%	4	80%		0%		0%		0%		0%		
HLA class II deficiency	5	4	80%	1	20%		0%		0%		0%	1	20%		
Hyper IgD syndrome (MVK)	5	1	20%	5	100%		0%		0%		0%		0%		
IPEX	5	1	20%	4	80%		0%		0%		0%		0%		
Netherton syndrome	5		0%	1	20%	3	60%		0%	2	40%		0%		
Omenn syndrome	5	2	40%	2	40%		0%		0%	1	20%		0%		
WHIM	5	4	80%	3	60%		0%		0%		0%	1	20%		

PID / presenting symptoms	PID patients with information	Infec-tions	%	Immune dysregu-lation	%	Syn-dromal	%	Mali-gnancy	%	Other sym-ptoms	%	No first sym-ptoms	%	Diagnosis by lab abnor-malities	%
Cartilage hair hypoplasia	4	3	75%		0%	4	100%		0%		0%		0%		
Chediak Higashi syndrome	4	3	75%	1	25%	1	25%		0%	1	25%		0%		
Cyclic neutropenia	4	3	75%	1	25%		0%		0%		0%		0%		
IgM deficiency	4	4	100%		0%		0%		0%		0%		0%		
Ivemark syndrome	4	1	25%		0%	2	50%		0%	1	25%		0%		
APDS	3	3	100%	1	33%		0%	1	33%		0%		0%		
APECED	3		0%	1	33%		0%		0%	2	67%		0%		
C8 deficiency	3	1	33%		0%		0%		0%		0%	2	67%		
MonoMAC	3	1	33%	1	33%		0%		0%	1	33%		0%		
Steinert myotonica dystrophia	3	1	33%	1	33%	1	33%		0%		0%		0%		
Early-onset multi-organ autoimmune disease	2	1	50%	2	100%		0%		0%		0%		0%		
FCAS	2		0%		0%		0%		0%	2	100%		0%		
Griscelli, type 2	2		0%	1	50%		0%		0%		0%	1	50%		
ICF	2	2	100%	1	50%	1	50%		0%		0%		0%		
Isolated congenital asplenia	2		0%		0%		0%		0%		0%	2	100%	2	100%
LAD	2	2	100%		0%		0%		0%		0%		0%		
PFC	2	2	100%		0%		0%		0%		0%		0%		
Thymoma with immunodeficiency	2	1	50%		0%		0%	2	100%		0%		0%		
Acquired angioedema	1		0%	1	100%		0%		0%		0%		0%		
Bloom syndrome	1		0%		0%	1	100%		0%		0%		0%		
C1 deficiency	1	1	100%	1	100%		0%		0%		0%		0%		
C7 deficiency	1	1	100%		0%		0%		0%		0%		0%		
CD25 deficiency	1	1	100%		0%	1	100%		0%		0%		0%		
CD27 deficiency	1	1	100%		0%		0%		0%		0%		0%		
CD4 deficiency	1		0%	1	100%		0%		0%		0%		0%		

PID / presenting symptoms	PID patients with information	Infec-tions	%	Immune dysregu-lation	%	Syn-dromal	%	Mali-gnancy	%	Other sym-ptoms	%	No first sym-ptoms	%	Diagnosis by lab abnor-malities	%
G6PD	1	1	100%		0%		0%		0%		0%		0%		
GSD 1b	1		0%		0%		0%		0%		0%	1	100%	1	100%
Hermansky-Pudlak syndrome	1		0%	1	100%		0%		0%		0%		0%		
IBD-like	1		0%	1	100%		0%		0%	1	100%		0%		
MBL	1	1	100%		0%		0%		0%		0%		0%		
PMS2 deficiency	1	1	100%		0%		0%		0%		0%		0%		
RALD	1	1	100%	1	100%		0%		0%		0%		0%		
Reticular Dysgenesis - AK2 (SCID)	1		0%		0%		0%		0%	1	100%		0%		
Type 1 interferonopathies	1	1	100%	1	100%		0%		0%		0%		0%		
**Total**	**2,153**	**1,598**	**74%**	**547**	**25%**	**190**	**9%**	**19**	**1%**	**248**	**12%**	**93**	**4%**	**55**	**3%**
**Female**	**927**	**690**	**74%**	**248**	**27%**	**87**	**9%**	**5**	**0,54%**	**97**	**10%**	**41**	**4%**	**26**	**3%**
**Male**	**1,226**	**908**	**74%**	**299**	**24%**	**103**	**8%**	**14**	**1,14%**	**151**	**12%**	**52**	**4%**	**29**	**2%**

S39: Presenting symptoms

pink: 50% and more, less than 15 patients, dark pink: 50% and more, more than 15 patients

**Top 12 PIDs / Age**	**0**	**01 to 5**	**06 to 10**	**11 to 15**	**16 to 20**	**21 to 25**	**26 to 30**	**31 to 35**	**36 to 40**	**41 to 45**	**46 to 50**	**51 to 100**	**Total**
CVID	55	132	73	62	74	36	45	40	39	29	16	33	634
Unclassified antibody deficiency	36	74	22	16	10	8	13	9	8	8	9	9	222
Isolated IgG subclass deficiency	21	22	3	8	3	3	2	7	5	3	1	3	81
Unclassified IDs	12	31	6	4	2	1	2	2		1	1	2	64
Selective IgA deficiency	9	28	4	4	3	2	2	2		1	1	2	58
CGD	45	52	10	3	4								114
Combined ID	30	20	6	8	2						2		68
Agammaglobulinemia	48	46	4		1		3						102
HIES	30	17	3		1		1						52
SCID	60	9	1										70
A-T	6	52	2										60
DGS	46	9		1									56
**Total (Top 12)**	**398**	**492**	**134**	**106**	**100**	**50**	**68**	**60**	**52**	**42**	**30**	**49**	**1,581**
%	25	31	8	7	6	3	4	4	3	3	2	3	100
**Total (all PID)**	**616**	**680**	**201**	**133**	**120**	**54**	**74**	**64**	**54**	**46**	**34**	**53**	**2,129**
%	29	32	9	6	6	3	3	3	3	2	2	2	100

S40: Onset of the 12 most common PIDs

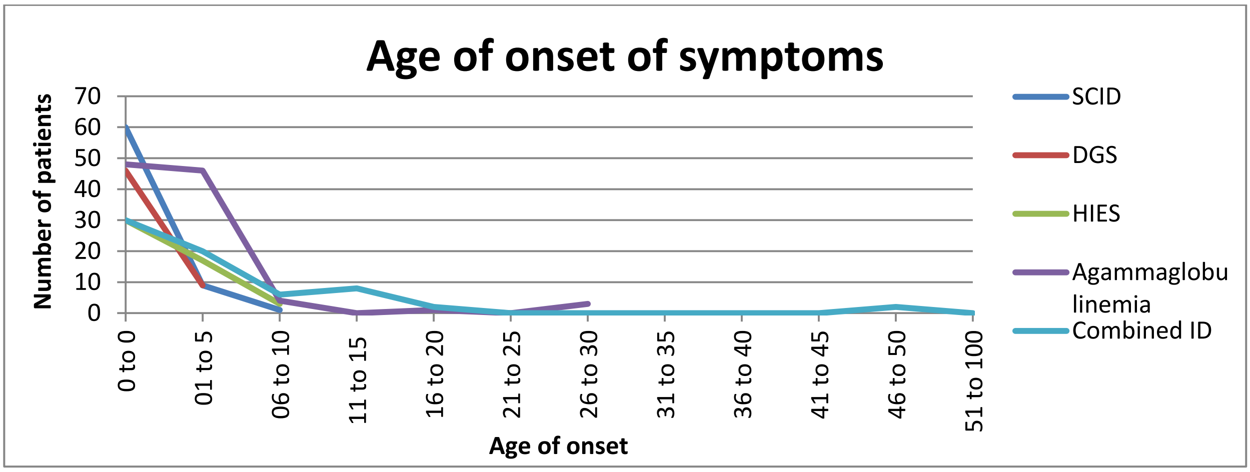

S41: Onset of symptoms mainly in the first year of life

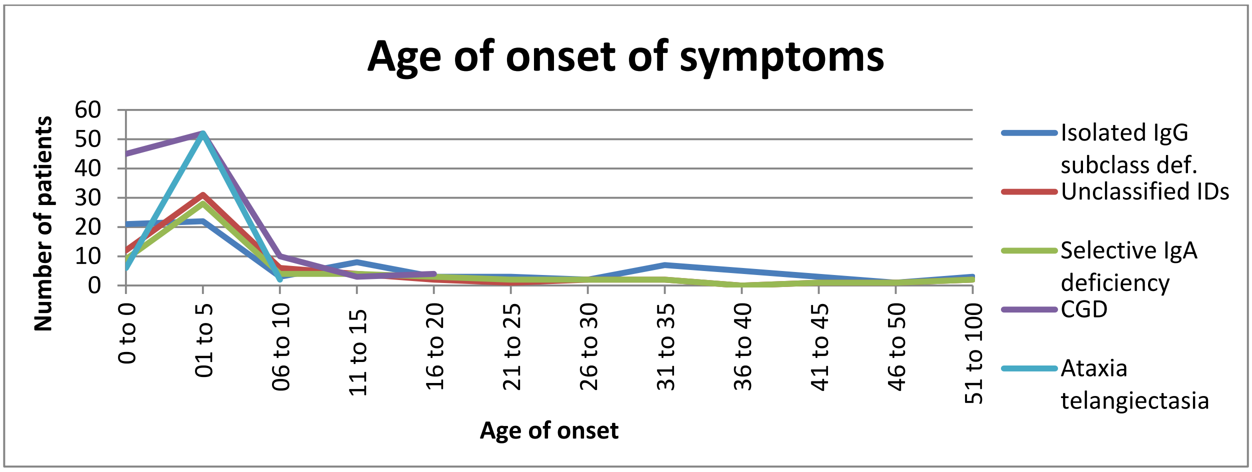

S42: Onset of symptoms mainly between the ages of 1 to 5

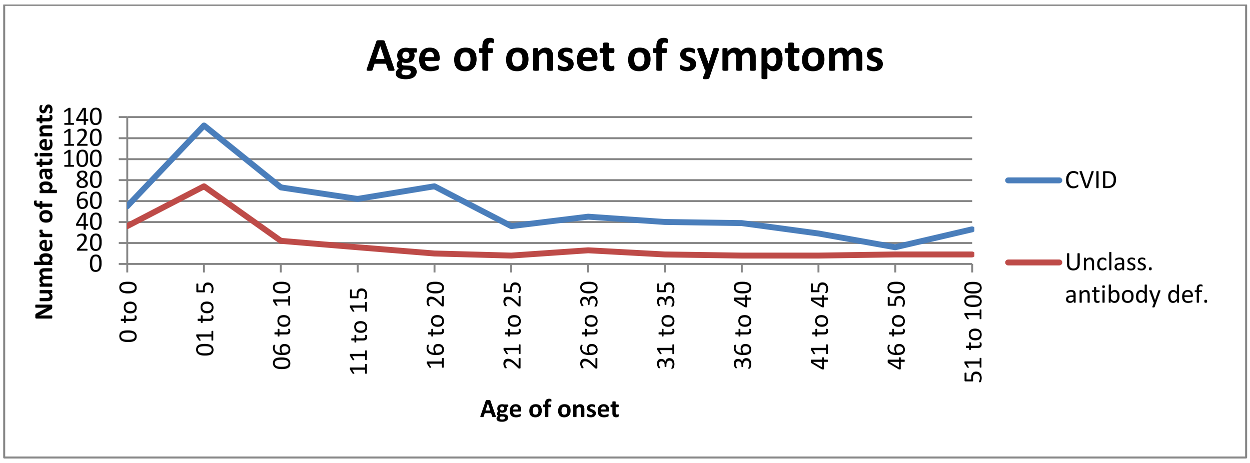

S43: Onset of symptoms with main onset age of 1 to 5 years and late onset

Main category	PID	Gene	Number of patients	Symptoms after genetic diagnosis [years]
**Complement deficiencies**	HAE (C1Inh)	*C1 Inhibitor*	2	Between 1 and 4
**Diseases of immune dysregulation**	ALPS	*FAS (TNFRSF6)*	1	1
	FHLH	*PRF1*	1	1*
**Other well defined PIDs**	Cartilage hair hypoplasia	*RMRP*	1	2
	DGS	*Del 22q11.2*	2	1**
	HIES	*STAT3*	1	1
	XLT (WASP)	*WASP* (X-linked)	1	1
**Phagocytic disorders**	G6PD	*G6PD*	1	1

S44: Genetic diagnosis before onset of symptoms

* prenatal genetic diagnosis
** One of the two patients had a prenatal diagnosis.

12 PID	Patients	Main category / PID / Age of diagnosis		0	1	2	3	4	5	6	7	8	9	10	11	12	13	14	15	16	17	Total	%
	82	**Autoinflammatory disorders**		3	8	7	7	11	8	4	4	5	1	5	2		1	1	2	1		70	85%
	2	FCAS						1							1							2	100%
	24	FMF			5		2	5	3		2	1	1	2					1			22	92%
	5	Hyper IgD syndrome (MVK)				2		1						1								4	80%
	18	TRAPS			3	3			2	2	1	1		1	1			1	1			16	89%
	20	Unclassified autoinflammatory				2	5	3	3	1	1	2		1								18	90%
	169	**Combined immunodeficiencies**		58	38	8	5	7	7	1	4	1	3	2	6	7	2	2	3	2	2	158	93%
	7	Atypical SCID		1	1	1		1			1				1					1		7	100%
	2	CD4 deficiency			1						1											2	100%
x	71	Combined ID		8	7	5	4	3	7	1	1	1	3	2	4	7	2	1	2	1	2	61	86%
	5	HLA class II deficiency		1	1	1												1	1			5	100%
	5	Omenn syndrome		2	2																	4	80%
	1	Reticular Dysgenesis - AK2 (SCID)		1																		1	100%
x	78	SCID		45	26	1	1	2			1				1							77	100%
	1	C1 deficiency						1														1	100%
	6	C2 deficiency			1	1		1		1		1										5	83%
	1	C7 deficiency													1							1	100%
	1	Factor I deficiency											1									1	100%
	23	HAE (C1Inh)		1	3	2	1	2	2		1				1	2	2		2		2	21	91%
	42	**Defects in innate immunity**		4	8	4	6		1		1	1	2	1		1	4			2	1	36	86%
	18	CMC		1	3	3	3		1					1			2			1	1	16	89%
	11	Innate ID, unclassified		1	2	1	1					1	1				1			1		9	82%
	8	TLR/NFkappa-B		2	3		2				1											8	100%
	134	**Diseases of immune dysregulation**		19	15	6	9	8	6	8	6	3	6	3	8	5	3	6	3	1	4	119	89%
	38	ALPS		2	4	2	4	5	2		1	1	2		2	4	1	3	1			34	89%
	4	APECED					1		1		1		1									4	100%
	1	CD25 deficiency			1																	1	100%
	1	CD27 deficiency															1					1	100%
	4	Chediak Higashi syndrome		2											1							3	75%
	5	Early onset of IBD		2	1	1					1											5	100%
	2	Early-onset multi-organ AI							1			1										2	100%
	20	FHLH		10	3			1		1				1			1		1	1		19	95%
	3	Griscelli, type 2		2															1			3	100%
	1	Hermansky-Pudlak syndrome													1							1	100%
	1	IBD-like			1																	1	100%
	18	Immune dysregulation, unclassified			4					2	1		3		2			2			1	15	83%
	5	IPEX		1	1	1	2															5	100%
	1	RALD				1																1	100%
	1	Type 1 interferonopathies								1												1	100%
	29	XLP				1	2	2	2	4	2	1		2	2	1	1				3	23	79%
	274	**Other well defined PIDs**		67	35	33	26	15	12	11	8	7	6	9	4	8	4	5		1	5	258	94%
	3	APDS		1		1			1													3	100%
x	63	ATM			5	18	15	4	6	3	4	3	2	2		1						63	100%
	1	Bloom syndrome										1										1	100%
	4	Cartilage hair hypoplasia		1											1		1				1	4	100%
x	64	DGS	2	32	13	3	1	1	2	1	1	1						2			1	60	94%
	7	Dyskeratosis congenita				2				1			1		1						1	6	86%
	1	Fc receptor deficiencies												1								1	100%
x	54	HIES		8	4	4	3	6	1	3	2	1	2	1	2	4	2	3			1	47	87%
	2	ICF		1			1															2	100%
	2	Isolated congenital asplenia		1		1																2	100%
	4	Ivemark syndrome		3	1																	4	100%
	10	NBS1		2	1		1	1	1				1	3								10	100%
	5	Netherton syndrome		3						1											1	5	100%
	1	PMS2 deficiency			1																	1	100%
	2	Schimke disease					1		1													2	100%
	14	Syndromic PID, unclassified		1		1	1	3		1	1	1		2		2						13	93%
	27	WAS		11	7	3	3			1						1						26	96%
	7	XLT (WASP)		3	3																	6	86%
	186	**Phagocytic disorders**	1	43	31	19	20	9	7	12	5	7	4	3	4	4	4	1			1	175	94%
x	123	CGD	1	25	18	16	18	4	4	7	2	6	2	2	2	3	2	1				113	92%
	28	Congenital neutropenia		7	3	3	2		3	2	1	1			2	1	2				1	28	100%
	4	Cyclic neutropenia		2	1			1														4	100%
	1	G6PD		1																		1	100%
	1	GSD I		1																		1	100%
	2	LAD		1	1																	2	100%
	8	MSMD		2	1			2		1	1											7	88%
	11	Shwachman-Diamond-syndrome		4	3			1			1		2									11	100%
	8	Unclassified phagocytic disorders			4			1		2				1								8	100%
x	107	Agammaglobulinemia		16	32	24	10	8	4	2		2	2	1	1	1						103	95%
	37	CSR / HIGM (Hyper-IgM)		4	9	2	3	6	2		2			2					1			31	84%
	17	Specific IgG def (SPAD)		2	2			4	1	1	2		1	1				2				16	94%
	21	Transient hypogamma		2	16	1	1		1													21	100%
	6	CINCA syndrome		3													1					4	67%
	7	Muckle-Wells syndrome						1		1		1								1		4	57%
	42	**Complement deficiencies**		1	4	3	1	4	2	2	1	1	1		2	2	2		2	1	2	31	74%
	3	C8 deficiency																		1		1	33%
	4	Complement ID, unclassified								1												1	25%
	1	MBL																				0	0%
	2	PFC																				0	0%
	5	WHIM											1			1	1					3	60%
	3	MonoMAC															1			1		2	67%
	1324	**Predominantly antibody disorders**		36	86	65	64	52	#	#	#	#	#	27	#	#	17	21	27	#	17	654	49%
x	703	CVID		8	8	11	14	17	13	21	12	7	12	11	13	18	13	13	19	24	13	247	35%
x	69	IgA deficiency			2	4	4	3	11	2	1	3	2		1	1	1		2	1		38	55%
	24	IgA with IgG subclass deficiency			1		2	1	2	1		2			1	1		1		1		13	54%
x	92	IgG subclass deficiency			5	7	7	5	4	2	2	2	1	2	3	1	1	1	2	2		47	51%
	4	IgM deficiency							2													2	50%
	3	Steinert-Myotonica Dystrophia																				0	0%
	2	Thymoma																				0	0%
x	245	Unclassified antibody def.		4	11	16	23	8	8	3	10	8	8	10	3	10	2	4	3	1	4	136	55%
	61	**Unclassified Immunodeficiencies**		3	3	3	5	5	4	5	1	2	5	3	1	3	1				1	45	74%
x	61	Unclassified IDs		3	3	3	5	6	4	5	1	2	5	3	1	3	1				1	46	74%
	2314			234	##	#	#	#	95	75	59	51	54	53	#	#	#	#	37	37	#	1,546	67%

Main category / PID / Age of diagnosis	18	19	20	21	22	23	24	25	26	27	28	29	30	31	32	33	34	35	36	37	38	39	40	41	42	43	44	45	46	47	48	49	50	51	52	53	54	55	56	57	58	59	60	61	62	63	64	65	66	67	68	69	70	71	72	73	74	75	79	81	Total	%	Patients
**Autoinflammatory disorders**		2				2					1			1						1	1	1			1						1															1															12	15%	82
FCAS																																																													0	0%	2
FMF											1			1																																															2	8%	24
Hyper IgD syndrome (MVK)																					1																																								1	20%	5
TRAPS																						1			1																																				2	11%	18
Unclassified autoinflammatory		1				1																																																							2	10%	20
**Combined immunodeficiencies**	3						1			1	1						1	1						1											1	1																									11	7%	169
Atypical SCID																																																													0	0%	7
CD4 deficiency																																																													0	0%	2
Combined ID	3									1	1						1	1						1											1	1																									10	14%	71
HLA class II deficiency																																																													0	0%	5
Omenn syndrome							1																																																						1	20%	5
Reticular Dysgenesis - AK2 (SCID)																																																													0	0%	1
SCID																																																													0	0%	77
C1 deficiency																																																													0	0%	1
C2 deficiency																								1																																					1	17%	6
C7 deficiency																																																													0	0%	1
Factor I deficiency																																																													0	0%	1
HAE (C1Inh)				1							1																																																		2	9%	23
**Defects in innate immunity**					1	1								1									1								1			1																											6	14%	42
CMC					1									1																																															2	11%	18
Innate ID, unclassified						1																	1																																						2	18%	11
TLR/NFkappa-B																																																													0	0%	8
**Diseases of immune dysregulation**		2			1		2	1		1				1	1		1			1			1			2	1																																		15	11%	134
ALPS							1	1						1						1																																									4	11%	38
APECED																																																													0	0%	4
CD25 deficiency																																																													0	0%	1
CD27 deficiency																																																													0	0%	1
Chediak Higashi syndrome		1																																																											1	25%	4
Early onset of IBD																																																													0	0%	5
Early-onset multi-organ AI																																																													0	0%	2
FHLH										1																																																			1	5%	20
Griscelli, type 2																																																													0	0%	3
Hermansky-Pudlak syndrome																																																													0	0%	1
IBD-like																																																													0	0%	1
Immune dysregulation, unclassified																	1						1			1																																			3	17%	18
IPEX																																																													0	0%	5
RALD																																																													0	0%	1
Type 1 interferonopathies																																																													0	0%	1
XLP		1			1		1								1											1	1																																		6	21%	29
**Other well defined PIDs**	4		1	1			1			1			1	4			1									1			1																																16	6%	274
APDS																																																													0	0%	3
ATM																																																													0	0%	63
Bloom syndrome																																																													0	0%	1
Cartilage hair hypoplasia																																																													0	0%	4
DGS							1			1			1	1																																															4	6%	64
Dyskeratosis congenita																										1																																			1	14%	7
Fc receptor deficiencies																																																													0	0%	1
HIES	3													3			1																																												7	13%	54
ICF																																																													0	0%	2
Isolated congenital asplenia																																																													0	0%	2
Ivemark syndrome																																																													0	0%	4
NBS1																																																													0	0%	10
Netherton syndrome																																																													0	0%	5
PMS2 deficiency																																																													0	0%	1
Schimke disease																																																													0	0%	2
Syndromic PID, unclassified			1																																																										1	7%	14
WAS				1																																																									1	4%	27
XLT (WASP)																													1																																1	14%	7
**Phagocytic disorders**	1	2	1		1	1											1			1		1				1															1																				11	6%	186
CGD	1	2	1			1											1			1		1				1															1																				10	8%	123
Congenital neutropenia																																																													0	0%	28
Cyclic neutropenia																																																													0	0%	4
G6PD																																																													0	0%	1
GSD I																																																													0	0%	1
LAD																																																													0	0%	2
MSMD					1																																																								1	13%	8
Shwachman-Diamond-syndrome																																																													0	0%	11
Unclassified phagocytic disorders																																																													0	0%	8
Agammaglobulinemia		1	1										2														1																																		5	5%	108
CSR / HIGM (Hyper-IgM)										1									1	1								1						1																	1										6	16%	37
Specific IgG def (SPAD)																						1																																							1	6%	17
Transient hypogamma																																																													0	0%	21
CINCA syndrome						1																									1																														2	33%	6
Muckle-Wells syndrome		1																		1																										1															3	43%	7
**Complement deficiencies**	3	2		2							1													2																1																					11	26%	42
C8 deficiency	1			1																																																									2	67%	3
Complement ID, unclassified	2																																							1																					3	75%	4
MBL																								1																																					1	100%	1
PFC		2																																																											2	100%	2
WHIM																															1			1																											2	40%	5
MonoMAC	1																																																												1	33%	3
**Predominantly antibody disorders**	14	16	15	18	7	9	15	19	20	10	19	20	14	16	16	13	20	25	14	25	13	21	17	21	17	14	17	12	11	17	9	12	15	18	18	11	8	9	8	12	7	13	9	5	4	3	1	2	1	6	5	2	3		1	1	2	1	2	1	674	51%	1328
CVID	9	14	12	14	7	6	11	17	13	6	13	13	7	14	13	10	14	18	11	18	10	12	12	14	13	10	10	8	7	9	5	7	12	10	6	6	5	6	6	7	6	6	5	4	2	2	1	2	1	4	1	1	2		1	1	2		1		457	65%	704
IgA deficiency	3	1		1		2	1	1	2	1	2	1	1		2		1	2		1				1								1		1	2			1					1								1	1									31	45%	69
IgA with IgG subclass deficiency										1	1	2				1	1					1				1				1				1	1																										11	46%	24
IgG subclass deficiency								1				1	2			1	1	1		3	1	5	3			2	2		2	4			1	1	4		1	1		2		2		1	1						1									1	45	49%	92
IgM deficiency																																1								1																					2	50%	4
Steinert-Myotonica Dystrophia																											1															2																			3	100%	3
Thymoma																														1																				1											2	100%	2
Unclassified antibody def.	2		2	3		1	3		5	1	3	3	2	2	1	1	3	4	2	2	2	2	2	6	4	1	3	3	2	2	4	3	2	4	5	5	2	1	2	2	1	3	3		1	1				1	1		1				1	1			111	45%	247
**Unclassified Immunodeficiencies**				1						1		1						3		1			1							2	1			2																	1			2							16	26%	61
Unclassified IDs				1						1		1						3		1			1							2	1			2																	1			2							16	26%	62
	#	#	17	22	10	13	19	20	20	14	22	21	15	23	17	13	24	29	14	29	14	23	20	24	18	18	18	12	12	19	12	12	15	21	19	12	8	9	8	13	8	13	9	5	4	4	1	2	1	6	6	2	3	2	1	1	2	1	2	1	772	33%	2,318

S45: Age at diagnosis in 2,314 patients

Percentages: white: 0-50%, pink 51%-74%, dark pink: 75%-100%

12 PIDs	Number of patients	Patients with genetic defect	Average of genetic diagnostic delay [years]	Median of genetic diagnostic delay [years]
**CVID**	527	12	11.08	9
**HIES**	43	37	7.84	7
**Unclassified antibody deficiency**	168	2	7.00	7
**Combined ID**	51	21	6.90	5
**CGD**	89	75	4.55	2
**Agammaglobulinemia**	87	68	3.90	1
**A-T**	49	42	3.26	3
**SCID**	40	24	1.42	0.5
**DGS**	38	33	0.48	0
**Unclassified IDs**	50	0		
**Isolated IgG subclass deficiency**	64	0		
**Selective IgA deficiency**	37	0		
**Total**	**1,243**	**314**	**4.38**	**2**

S46: Average and median of genetic diagnostic delay

Main cause(s) leading to death																						Underlying morbid setting(s) associated with mortality				
PID	Gene	Number of patients	Age [year]	HSCT	Septic shock	Heart failure	Respiratory failure	Liver failure	Renal failure	Multiple organ failure	Haemorrhage	Thrombosis	Neurological complications	Surgical complications	Drug toxicity	Relapse of malignancy	Veno occlusive disorder	Graft versus host disease	Post-transplant lymphoproli-ferative disorder	Rejection/poor graft transplant	Other	Infection	Malignancy	Immune dysregulation	Transplantation related	Other
Ataxia telangiectasia (A-T)	*ATM*	6	11-38		2		2	1		1	1		1									3	2	1		
Atypical severe combined immuno-deficiency (Atypical SCID)	*RAG1*	1	23	1	1		1			1	1														1	
Cartilage hair hypoplasia	*RMRP*	1	18			1																			1	
Chronic granulomatous disease (CGD)		7		2			2		1	3	1			2				3				3		2	2	
	GP91-phox(CYBB)	4	6, 10,19, 20	2			2		1	2	1			1				3				2		1	2	
	P47-phox (NCF1)	1	62																							
	N/A	2	1							1				1								1		1		
Combined immuno-deficiency (CID)		5		2	1		2			3												4		1	1	
	Orai1 (TMEM142A)	1	1	1			1															1				
	PRKDC	2	7, 23							2												2				
	N/A	2	3,18	1	1		1			1												1		1	1	

Main cause(s) leading to death																						Underlying morbid setting(s) associated with mortality				
PID	Gene	Number of patients	Age [year]	HSCT	Septic shock	Heart failure	Respiratory failure	Liver failure	Renal failure	Multiple organ failure	Haemorrhage	Thrombosis	Neurological complications	Surgical complications	Drug toxicity	Relapse of malignancy	Veno occlusive disorder	Graft versus host disease	Post-transplant lymphoproliferative disorder	Rejection/poor graft transplant	Other	Infection	Malignancy	Immune dysregulation	Transplantation related	Other
Common variable immuno-deficiency disorders (CVID)	N/A	12	18-88	1	3	3		1		1	1										1 *	1	5	3	1	1 ***
Complement component 1 deficiency	*C1Q-beta*	1	14																		1 **			1		
Familial hemopha-gocytic lymphohi-stiocytosis syndromes (FHLH)	*PRF1*	1	10 month	1			1															1				
HLA class II deficiency	*RFXAP*	1	16	1						1												1				
Isolated IgG subclass deficiency	N/A	1	N/A																							
Nijmegen breakage syndrome (NBS1)	*NBS1*	1	15																							
Omenn syndrome	*RAG1*	1	5 month	1			1															1				
Properdin P factor complement deficiency (PFC)	N/A	1	18		1																	1				

Main cause(s) leading to death																						Underlying morbid setting(s) associated with mortality				
PID	Gene	Number of patients	Age [year]	HSCT	Septic shock	Heart failure	Respiratory failure	Liver failure	Renal failure	Multiple organ failure	Haemorrhage	Thrombosis	Neurological complications	Surgical complications	Drug toxicity	Relapse of malignancy	Veno occlusive disorder	Graft versus host disease	Post-transplant lymphoproliferative disorder	Rejection/poor graft transplant	Other	Infection	Malignancy	Immune dysregulation	Transplantation related	Other
Severe combined immuno-deficiency (SCID)		3		2	1		3			1												2				
	RAG1	1	1	1			1															1				
	N/A	2	4 month,	1	1		2			1												1				
Steinert myotonica dystrophia	*ZNF9*	1	70																							
Unclassified antibody deficiency		4		1			3			1						1						3	1			
	LRBA	1	12	1			1															1				
	N/A	3	14,17, 79				2			1						1						2	1			
Unclassified autoinflamma-tory diseases	N/A	1	14	1		1	1																	1		
Unclassified disorders of immune dysregulation	N/A	1	N/A																							

Main cause(s) leading to death																						Underlying morbid setting(s) associated with mortality				
PID	Gene	Number of patients	Age [year]	HSCT	Septic shock	Heart failure	Respiratory failure	Liver failure	Renal failure	Multiple organ failure	Haemorrhage	Thrombosis	Neurological complications	Surgical complications	Drug toxicity	Relapse of malignancy	Veno occlusive disorder	Graft versus host disease	Post-transplant lymphoproliferative disorder	Rejection/poor graft transplant	Other	Infection	Malignancy	Immune dysregulation	Transplantation related	Other
X-linked lympho-proliferative syndrome (XLP)		2		1						1															1	
	BIRC4/XIAP (XLP2)	1	19	1						1															1	
	SH2D1A (XLP1)	1	24																							
**Total**		**51**		**14**	**9**	**5**	**16**	**2**	**1**	**13**	**4**		**1**	**2**		**1**		**3**	**0**	**0**	**2**	**20**	**8**	**9**	**7**	**1**

S47: Cause of death for 51 patients

* Hemolysis due to T-LGL lymphoma, ** chronic pancreatitis, *** Heart disease, myocardial infection

PID	Number of patients (total)	IgG substitution unknown	%	No IgG substitution	%
**Selective IgA deficiency**	69	24	35%	45	65%
**Congenital neutropenia**	32	1	3%	31	97%
**FMF**	24	3	12%	21	88%
**HAE (C1Inh)**	23	3	13%	20	87%
**TRAPS**	18	3	17%	15	83%
**Shwachman-Diamond-syndrome**	11	0	0%	11	100%

S48: PID with no IgG substitution (PIDs with minimum 10 patients of 270 patients)

PID / IgG substitution delay [Years]	-20	-11	-10	-6	-5	-4	-3	-2	-1	0	1	2	3	4	5	6	7	8	9	10	11	12	13	14	15	16	17	18	19	20	21	22	23	24	26	27	29	30	31	34	36	40	41	53	Number of patients with information available date
CVID	1		1		1	2	3	7	9	346	94	28	8	14	6	8	8	9		8	4	2	4	3	3	1	3	1	6	2	2	1	2	1	1		1	1	1	1	1	1	2	1	**598**
Unclassified antibody def.			1				1		4	83	37	5	5	6	5	3	3	1		1	1				1		1		1	1															**160**
CGD											2		2						2						1																				**7**
Agamma-globulinemia								1	1	71	10		1	1	1	1	1		1	1						2			1								1								**94**
Isolated IgG subclass deficiency										33	10	5	4		3	1	1	1	1		1																								**60**
SCID										18		1			1																														**20**
Combined ID		1							2	25	9	1					2						1			1										1									**43**
Selective IgA deficiency											1																																		**1**
DGS								1	1		1								1																										**4**
A-T							1			6	2	2	1	3		1	2	1	1				1	1																					**22**
Unclassified IDs									2	11	2	1			1				1	1																		1	1						**21**
HIES								1		5	1	1		1	1			1		3			1					1																	**16**
ALPS												1																																	**1**
CSR/HIGM (Hyper-IgM)								1	1	19	3																											1							**25**
XLP									1	4	1													1																					**7**
Congenital neutropenia																																													**0**
WAS									1	3	2																							1											**7**
IgA with IgG subclass def.									1	8	2			1				1		1																									**14**
FMF																																													**0**
HAE (C1Inh)																																													**0**

PID / IgG substitution delay [Years]	-20	-11	-10	-6	-5	-4	-3	-2	-1	0	1	2	3	4	5	6	7	8	9	10	11	12	13	14	15	16	17	18	19	20	21	22	23	24	26	27	29	30	31	34	36	40	41	53	Number of patients with information available date
Transient hypogamma-globulinemia of infancy										5																																			**5**
FHLH										2																																			**2**
Unclassified autoinflamma-tory																																													**0**
CMC										1		1							1				1											1											**5**
Unclassified disorders of immune dysregulation										3								1																											**4**
TRAPS																																													**0**
Specific IgG def. (SPAD)						1				2	1	1		2	1																														**8**
Syndromic PID, unclassified				1						3	2		1		2																														**9**
Innate ID, unclassified										2	1	1																																	**4**
Shwachman-Diamond-syndrome																																													**0**
NBS1						1				1	1		1	1	1							1																							**7**
Unclassified phagocytic disorders												1																																	**1**
TLR/NF kappa-B								1		1	3	1																																	**6**
Nothing entered										2																																			**2**
MSMD											1				1																														**2**
Atypical SCID										1	1																																		**2**

PID / IgG substitution delay [Years]	-20	-11	-10	-6	-5	-4	-3	-2	-1	0	1	2	3	4	5	6	7	8	9	10	11	12	13	14	15	16	17	18	19	20	21	22	23	24	26	27	29	30	31	34	36	40	41	53	Number of patients with information available date
Dyskeratosis congenita									1	1																																			**2**
Muckle-Wells syndrome																																													**0**
XLT (WASP)																																													**0**
C2 deficiency										1	1																																		**2**
CINCA syndrome																																													**0**
Netherton syndrome										1	1		1																																**3**
HLA class II deficiency									1	1																																			**2**
Hyper IgD syndrome (MVK)											1																																		**1**
Omenn syndrome																						1																							**1**
WHIM											1																																		**1**
Early onset of IBD																																													**0**
IPEX																																													**0**
Cartilage hair hypoplasia									1						1																														**2**
IgM deficiency											1	1																																	**2**
APECED																																													**0**
Chediak Higashi syndrome																																													**0**
Complement ID, unclassified																																													**0**
Cyclic neutropenia																																													**0**
Ivemark syndrome																																													**0**
APDS										2												1																							**3**

PID / IgG substitution delay [Years]	-20	-11	-10	-6	-5	-4	-3	-2	-1	0	1	2	3	4	5	6	7	8	9	10	11	12	13	14	15	16	17	18	19	20	21	22	23	24	26	27	29	30	31	34	36	40	41	53	Number of patients with information available date
MonoMAC				1						1																																		**2**
Steinert myotonica dystrophia											1																																	**1**
C8 deficiency																																												**0**
Griscelli, type 2																																												**0**
Early-onset multi-organ AI							1				1																																	**2**
Thymoma with immunode-ficiency										2																																		**2**
ICF										1																																		**1**
CD4 deficiency																																												**0**
FCAS																																												**0**
Isolated congenital asplenia																																												**0**
LAD																																												**0**
PFC																																												**0**
Schimke disease																																												**0**
Bloom syndrome								1																																				**1**
C1 deficiency															1																													**1**
CD27 deficiency												1																																**1**
PMS2 deficiency										1																																		**1**
Type 1 interferono-pathies														1																														**1**
C7 deficiency																																												**0**
CD25 deficiency																																												**0**

PID / IgG substitution delay [Years]	-20	-11	-10	-6	-5	-4	-3	-2	-1	0	1	2	3	4	5	6	7	8	9	10	11	12	13	14	15	16	17	18	19	20	21	22	23	24	26	27	29	30	31	34	36	40	41	53	Number of patients with information available date
Factor I deficiency																																													**0**
Fc receptor deficiencies																																													**0**
G6PD																																													**0**
GSD 1b																																													**0**
Hermansky-Pudlak syndrome																																													**0**
IBD-like																																													**0**
MBL																																													**0**
RALD																																													**0**
Reticular Dysgenesis - AK2 (SCID)																																													**0**
**Total**	**1**	**1**	**2**	**2**	**1**	**4**	**6**	**13**	**26**	**666**	**194**	**52**	**24**	**30**	**25**	**14**	**17**	**15**	**8**	**15**	**6**	**5**	**8**	**5**	**5**	**4**	**4**	**2**	**8**	**3**	**2**	**1**	**2**	**3**	**1**	**1**	**2**	**3**	**2**	**1**	**1**	**1**	**2**	**1**	**1,189**

S49: IgG substitution delay

Relative IgG-dose [mg per kg body weight per month]	SIde effects sc	Side effects iv	sc	iv	Side effects total	No side effects sc	No side effects iv	No side effects total	Side effects sc of sc [%]	Side effects iv of iv [%]	No side effects total of total [%]	Total
**0-50**	2	0	2	5	2	0	3	3	100%	0%	43%	7
**50-100**	2	0	10	3	2	5	3	8	20%	0%	62%	13
**100-150**	2	0	13	6	2	11	5	16	15%	0%	84%	19
**150-200**	3	1	18	12	4	14	10	24	17%	8%	80%	30
**200-250**	3	0	27	7	3	22	6	28	11%	0%	82%	34
**250-300**	2	1	47	8	3	42	4	46	4%	13%	84%	55
**300-350**	5	6	61	32	11	50	21	71	8%	19%	76%	93
**350-400**	7	5	63	30	12	50	20	70	11%	17%	75%	93
**400-450**	9	5	115	28	14	94	18	112	8%	18%	78%	143
**450-500**	11	3	88	21	14	71	15	86	13%	14%	79%	109
**500-550**	7	2	70	23	9	58	21	79	10%	9%	85%	93
**550-600**	12	1	61	30	13	42	24	66	20%	3%	73%	91
**600-650**	8	0	46	19	8	36	17	53	17%	0%	82%	65
**650-700**	3	1	30	14	4	23	11	34	10%	7%	77%	44
**700-750**	2	1	32	13	3	27	12	39	6%	8%	87%	45
**750-800**	4	1	21	6	5	17	3	20	19%	17%	74%	27
**800-850**	2	0	16	5	2	13	5	18	13%	0%	86%	21
**850-900**	0	0	5	1	0	4	0	4	0%	0%	67%	6
**900-950**	2	1	7	4	3	5	3	8	29%	25%	73%	11
**950-1000**	2	0	8	2	2	5	2	7	25%	0%	70%	10
**1000-1050**	0	0	5	1	0	5	0	5	0%	0%	83%	6
**1050-1100**	1	1	7	2	2	6	0	6	14%	50%	67%	9
**1100-1150**	0	0	2	2	0	2	2	4	0%	0%	100%	4
**1150-1200**	0	0	5	1	0	5	1	6	0%	0%	100%	6
**1200-1250**	0	0	1	0	0	1	0	1	0%		100%	1
**1250-1300**	1	0	3	4	1	2	3	5	33%	0%	71%	7
**1300-1350**	0	0	1	0	0	1	0	1	0%		100%	1
**1400-1450**	0	0	4	1	0	3	1	4	0%	0%	80%	5

Relative IgG-dose [mg per kg body weight per month]	SIde effects sc	Side effects iv	sc	iv	Side effects total	No side effects sc	No side effects iv	No side effects total	Side effects sc of sc [%]	Side effects iv of iv [%]	No side effects total of total [%]	Total
**1550-1600**	0	0	1	0	0	1	0	1	0%		100%	1
**1600-1650**	0	0	1	0	0	1	0	1	0%		100%	1
**1700-1750**	0	1	2	1	1	2	0	2	0%	100%	67%	3
**1800-1850**	0	0	1	0	0	1	0	1	0%		100%	1
**1950-2000**	0	0	1	0	0	1	0	1	0%		100%	1
**2000-2050**	0	0	1	0	0	1	0	1	0%		100%	1
**2100-2150**	0	0	1	1	0	1	1	2	0%	0%	100%	2
**2200-2250**	0	0	1	0	0	1	0	1	0%		100%	1
**2250-2300**	0	0	1	0	0	1	0	1	0%		100%	1
**2750-2800**	0	0	1	0	0	1	0	1	0%		100%	1
	**90**	**30**	**779**	**282**	**120**	**625**	**211**	**836**	**12%**	**11%**	**79%**	**1,061**

S50: Relative IgG-dose [mg per kg body weight per month] and side effects (1,061 patients)

PID	Total	HSCT	HSCT (%)
**SCID**	83	72	87%
**CGD**	129	61	47%
**Combined ID**	74	28	38%
**WAS**	29	20	69%
**FHLH**	20	16	80%
**CSR/HIGM (Hyper-IgM)**	38	11	29%
**XLP**	29	9	31%
**HIES**	55	8	15%
**Congenital neutropenia**	32	7	22%
**IPEX**	5	5	100%
**Atypical SCID**	7	5	71%
**Dyskeratosis congenita**	7	5	71%
**MSMD**	9	4	44%
**Omenn syndrome**	5	4	80%
**HLA class II deficiency**	5	4	80%
**Griscelli, type 2**	3	3	100%
**MonoMAC**	3	3	100%
**Unclassified IDs**	76	3	4%
**Chediak Higashi syndrome**	4	3	75%
**NBS1**	10	3	30%
**Early onset of IBD**	5	3	60%
**Unclassified phagocytic disorders**	10	3	30%
**Unclassified antibody def.**	267	2	1%
**LAD**	2	2	100%
**Agammaglobulinemia**	112	2	2%
**Cartilage hair hypoplasia**	4	2	50%
**XLT (WASP)**	7	2	29%
**CVID**	728	2	0,2%
**Immune dysregulation, unclassified**	20	2	10%
**Syndromic PID, unclassified**	16	1	6%
**Unclassified autoinflammatory**	22	1	5%
**TLR/NFkappa-B**	9	1	11%
**Reticular Dysgenesis - AK2 (SCID)**	1	1	100%
**Shwachman-Diamond-syndrome**	11	1	9%
**A-T**	66	1	2%
**Isolated IgG subclass deficiency**	101		0%
**Selective IgA deficiency**	69		0%
**DGS**	65		0%
**ALPS**	38		0%
**IgA with IgG subclass deficiency**	26		0%
**FMF**	24		0%
**HAE (C1Inh)**	23		0%
**Transient hypogamma globulinemia of infancy**	21		0%
**No PID entered**	19		0%
**CMC**	19		0%
**Specific IgG deficiency (SPAD)**	18		0%
**TRAPS**	18		0%
**Innate ID, unclassified**	12		0%
**Muckle-Wells syndrome**	7		0%

PID	Total	HSCT	HSCT (%)
C2 deficiency	6		0%
CINCA syndrome	6		0%
Netherton syndrome	5		0%
Complement ID, unclassified	5		0%
IgM deficiency	5		0%
WHIM	5		0%
Hyper IgD syndrome (MVK)	5		0%
Ivemark syndrome	4		0%
APECED	4		0%
Cyclic neutropenia	4		0%
APDS	3		0%
C8 deficiency	3		0%
Steinert myotonica dystrophia	3		0%
CD4 deficiency	2		0%
FCAS	2		0%
ICF	2		0%
PFC	2		0%
Schimke disease	2		0%
Isolated congenital asplenia	2		0%
Thymoma with immunodeficiency	2		0%
Early-onset multi-organ AI	2		0%
Factor I deficiency	1		0%
C1 deficiency	1		0%
CD25 deficiency	1		0%
GSD 1b	1		0%
Hermansky-Pudlak syndrome	1		0%
PMS2 deficiency	1		0%
C7 deficiency	1		0%
CD27 deficiency	1		0%
IBD-like	1		0%
Bloom syndrome	1		0%
G6PD	1		0%
RALD	1		0%
Fc receptor deficiencies	1		0%
Type 1 interferonopathies	1		0%
MBL	1		0%
Acquired angioedema	1		0%
**Total**	**2,453**	**300**	**12%**

S51: Patients with HSCT

Main group / sub group	Number of patients
Autoinflammatory disorders	1
Unclassified autoinflammatory diseases	1
Combined immunodeficiencies	21
Atypical severe combined immunodeficiency (Atypical SCID)	3
Combined immunodeficiency (CID)	5
Severe combined immunodeficiency (SCID)	13
Diseases of immune dysregulation	5
Hemophagocytic lymphohistiocytosis (HLH)	4
Unclassified disorders of immune dysregulation	1
Other well defined PIDs	6
DNA-breakage disorder (*NBS1*)	1
Hyper IgE syndromes *(DOCK8)*	1
MonoMAC (*GATA2*)	2
Wiskott-Aldrich syndrome *(WASP* (X-linked)*)*	2
Phagocytic disorders	5
Chronic granulomatous disease *(GP91-phox(CYBB))*	4
Unclassified phagocytic disorders	1
Predominantly antibody disorders	6
Agammaglobulinemias (*BTK*, X-linked)	1
Class switch recombination defects (CSR) / HIGM syndromes *CD40L (CD154)*	1
Unclassified antibody deficiency *(LRBA)*	1
**Total**	**42**

S52: Patients with HSCT and Ig treatment after HSCT

Age distribution by year of birth

When analysing all 2,453 registered patients (and also the 12 most frequent PIDs), they exhibited a bimodal distribution according to their year of birth. The first peak contained patients currently aged 8 to 23, hence born between 1995 and 2007. The second peak contained patients aged 50 to 60, born between 1967 and 1959 (S55 and S56).

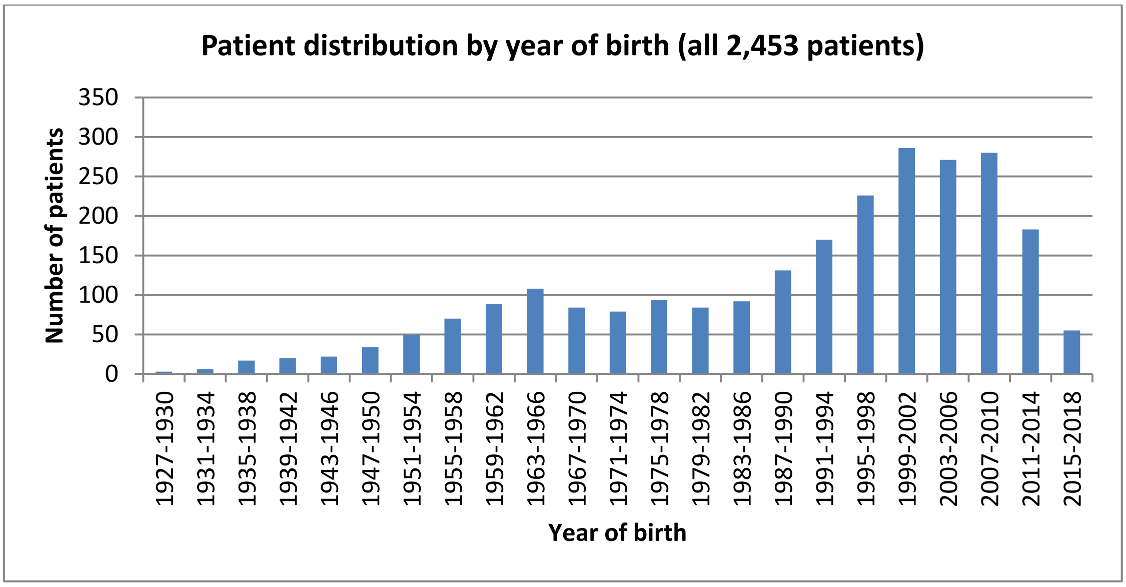

S53: Age distribution by year of birth of 2,453 patients

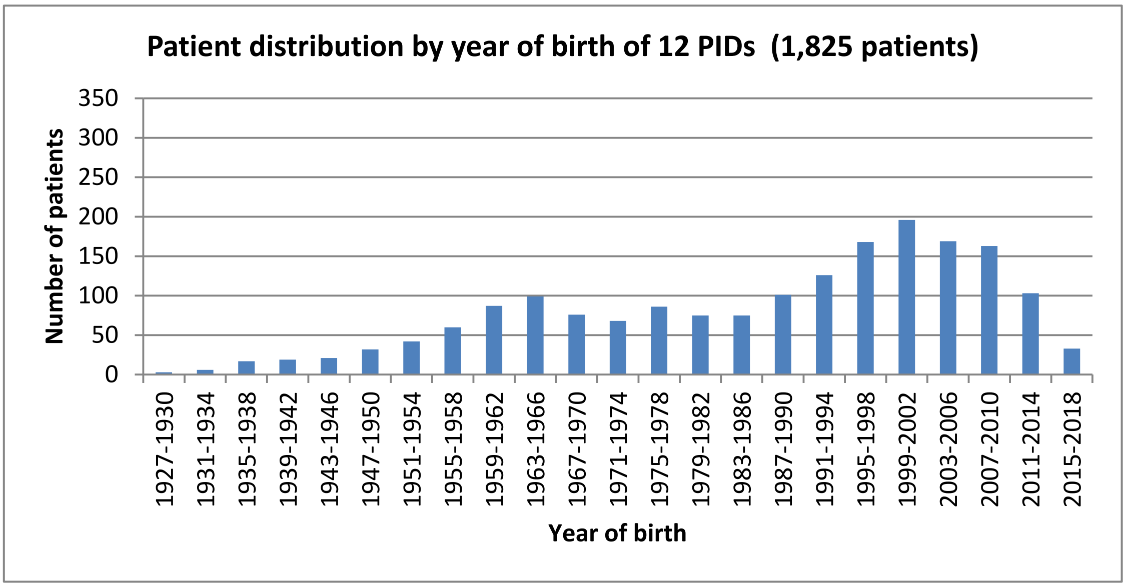

S54: Age distribution by year of birth of 12 PIDs (1,825 patients)

When we compared the individual PIDs with the highest numbers of registered patients (12 PIDs), quite different age curves appeared (S57, S58): For SCID and A-T, the majority of patients were aged 20 and younger, whereas most patients with CVID were aged 20 to 60. The oldest CVID patient was born in 1928 and the youngest registered one in 2011. Selective IgA deficiency and isolated IgG subclass deficiency exhibited a similar distribution to CVID, with a slight elevation between 1995 and 2010. A similar curve was also observed for patients with unclassified IDs.

The curves of SCID, A-T, and DGS patients were similar: The first patient with DGS was born in 1975, the first A-T patient in 1977 and the first patient with SCID in 1988. An increase of patients was recorded between 2003 and 2014. The oldest registered HIES patient was born in 1973 and the youngest in 2016. Here, we found that most patients were aged (11 patients) (S59).

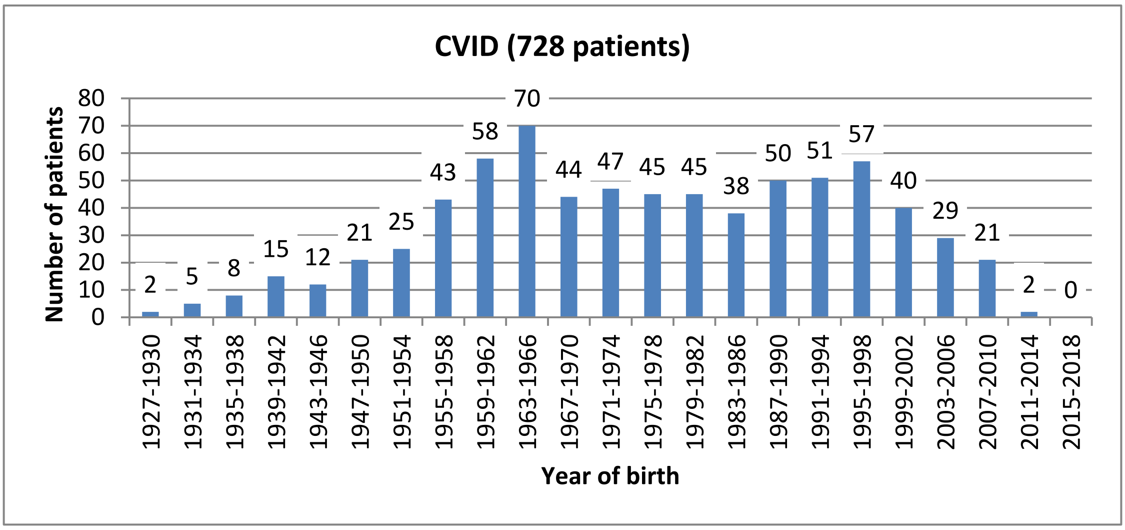

S55: Age distribution by year of birth of 728 CVID patients

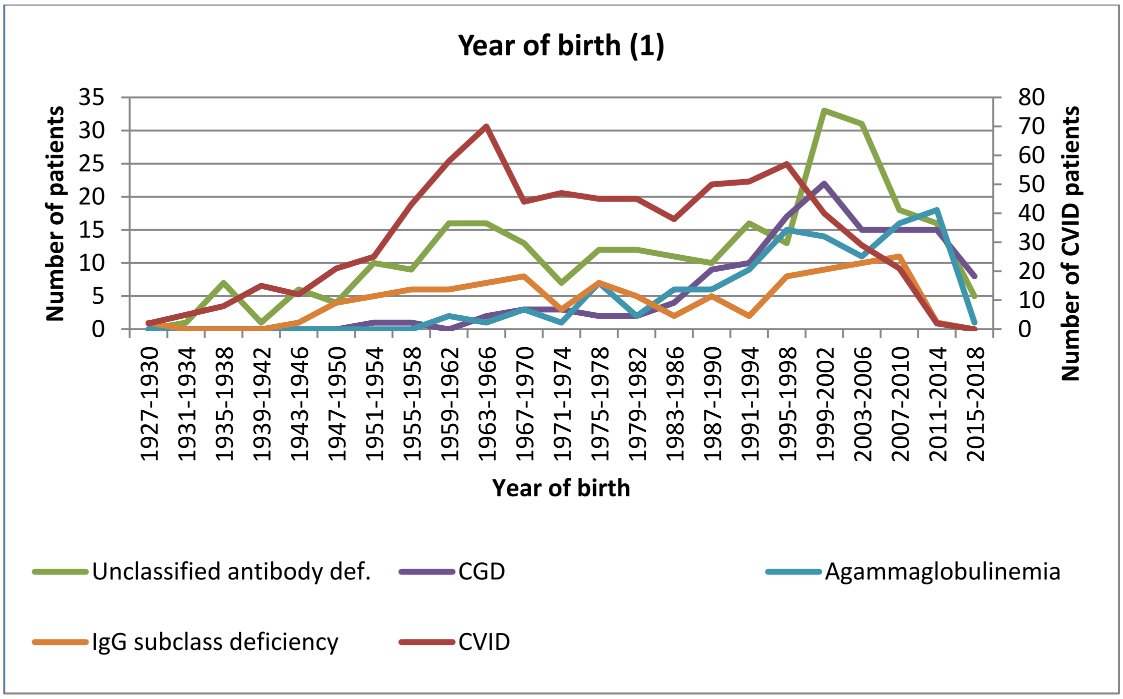

S56: Age distribution by year of birth of the 5 PIDs with the most patients (all registered patients)

Please note the different scaling of the number of CVID patients. Only one male patient born in 2007 had a HSCT treatment. We grouped the year of birth of all registered patients in 4-year span similar to Gathmann B et al. [3].

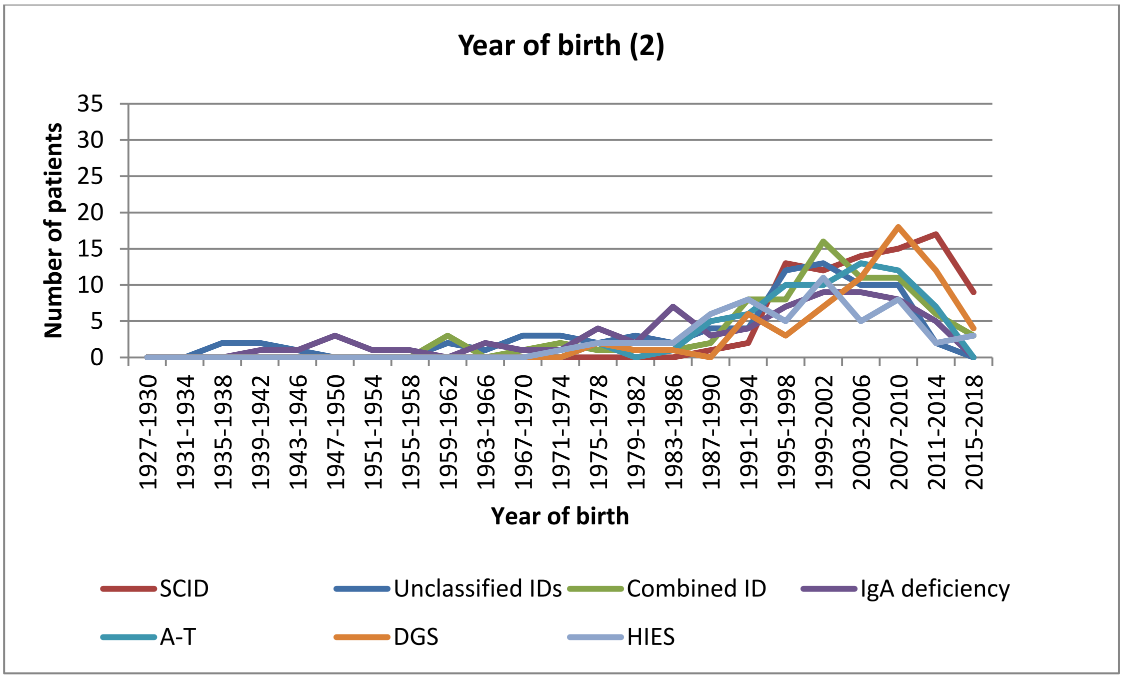

S57: Age distribution by year of birth of the first 7-12 PIDs with the most patients (all registered patients)

We grouped the year of birth of all registered patients in 4-year span similar to Gathmann B et al. [3].
